# Inorganic and hybrid nanomaterials for NIR-II fluorescence imaging-guided therapy of Glioblastoma and perspectives

**DOI:** 10.7150/thno.112204

**Published:** 2025-04-21

**Authors:** Zhigang Li, Lixin Du, Binghua Du, Zia Ullah, Yinghe Zhang, Yanyang Tu, Ying Zhou, Bing Guo

**Affiliations:** 1 Department of Medical Imaging, Shenzhen Longhua District Central Hospital, Shenzhen Longhua District Key Laboratory of Neuroimaging, Shenzhen 518110, China.; 2 School of Science, Shenzhen Key Laboratory of Advanced Functional Carbon Materials Research and Comprehensive Application, Harbin Institute of Technology, Shenzhen 518055, China.; 3 Research Center, Huizhou Central People's Hospital, Guangdong Medical University, Huizhou City, Guangdong Province, China.; 4 Department of Pharmacy, Peking University First Hospital, Beijing, China.

**Keywords:** Glioblastoma, NIR-II fluorescence imaging, imaging-guided therapy, inorganic and hybrid nanomaterials, targeting

## Abstract

Glioblastoma (GBM) is the most invasive and lethal brain tumor, with limited therapeutic options due to its highly infiltrative nature, resistance to conventional therapies, and blood-brain barriers. Recent advancements in near-infrared II (NIR-II) fluorescence imaging have facilitated greater tissue penetration, improved resolution, and real-time visualization of GBM, providing a promising approach for precise diagnosis and treatment. The inorganic and hybrid NIR-II fluorescent materials have developed rapidly for NIR-II fluorescence imaging-guided diagnosis and therapy of many diseases, including GBM. Herein, we offer a timely update to explore the contribution of inorganic/hybrid NIR-II fluorescent nanomaterials, such as quantum dots, rare-earth-doped nanoparticles, carbon-based nanomaterials, and metal nanoclusters in imaging-guided treatment for GBM. These nanomaterials provide high photostability, strong fluorescence intensity, and tunable optical properties, allowing for multimodal imaging and enhanced therapeutic efficacy. Additionally, their integration with modern therapeutic strategies, such as photothermal therapy, chemodynamic therapy, photodynamic therapy, sonodynamic therapy, and immunotherapy, has shown significant potential in overcoming the limitations of traditional treatments. Looking forward, future advancements including safe body clearance, long-term biocompatibility, efficient BBB penetration, and extended emission wavelengths beyond 1500 nm could enhance the theranostic outcomes. The integration of dual imaging with immunotherapy and AI-driven strategies will further enhance precision and accelerate the clinical translation of smart theranostic platforms for GBM treatment.

## 1. Introduction

Glioblastoma multiforme (GBM) is classified as a grade IV brain tumor by the World Health Organization (WHO) and is among the deadliest and most aggressive intracranial tumors in humans 1. Accounting for approximately 14% of all primary brain tumors, GBM can develop at any age but is most frequently observed in older adults between 65 and 74 years. The average age of diagnosis is 65, with over 12,000 new reported cases annually in the United States of America 2. While the exact cause of GBM remains unclear, most cases occur without any family history or known reasons. However, individuals with conditions like neurofibromatosis, Li-Fraumeni syndrome, Lynch syndrome, Turcot syndrome, or constitutional mismatch repair deficiency syndrome are at greater risk of high-grade brain tumors, including GBM 3. Additionally, ionizing radiation exposure, such as from childhood radiotherapy for brain cancer or leukemia, is also a recognized reason 4,5. Genetic analyses and mouse model studies suggested that GBM originates from neural stem cells, neural stem cells derived astrocytes, and oligodendrocyte precursor cells 6. Causing the cancer because of the above-mentioned origins exhibited distinct features in animal tumor models. However, traditional 2D culture models presented challenges in studying GBM mechanisms due to the lack of a human-like microenvironment. Consequently, 3D and organoid models have been developed worldwide to better understand the pathology of GBM to efficiently control and treat it for better healthcare 7.

Conventionally, a combination of surgery, chemotherapy with Temozolomide (TMZ), and radiotherapy has been a primary strategy for treating GBM patients clinically 8. However, these treatment modalities have limited efficacy due to several factors, including the high recurrence rate resulting from the infiltrative nature of GBM, radiation-induced severe side effects, glioma stem cells attributed to multidrug resistance, and blood-brain barriers (BBB) 9. Consequently, full recovery for GBM patients remains extremely rare, with current diagnostic and therapeutic modalities yielding less than 6.9% of the 5-year survival rate 10,11. Effective treatment of GBM requires immediate advancements in accurate diagnosis and efficient therapeutic strategies. For diagnosing GBM, conventional diagnostic techniques include computed tomography (CT), magnetic resonance imaging (MRI), positron emission tomography (PET), and ultrasound imaging (USI) 12. However, these techniques face limitations such as insufficient tissue selectivity, high costs, harmful ionization effects, long acquisition times, and challenges in real-time visualization. Therefore, there is an urgent need to develop non-invasive imaging techniques capable of deep penetration, high-resolution, and real-time tumor visualization. Specifically, fluorescence (FL) imaging offers rapid feedback, low toxicity, high-resolution, and real-time tumor monitoring, making it an attractive option for researchers to diagnose GBM 13. Additionally, patient- and operator-friendly imaging setups enable precise differentiation between tumor lesions and normal brain tissues.

**Figure 1:**
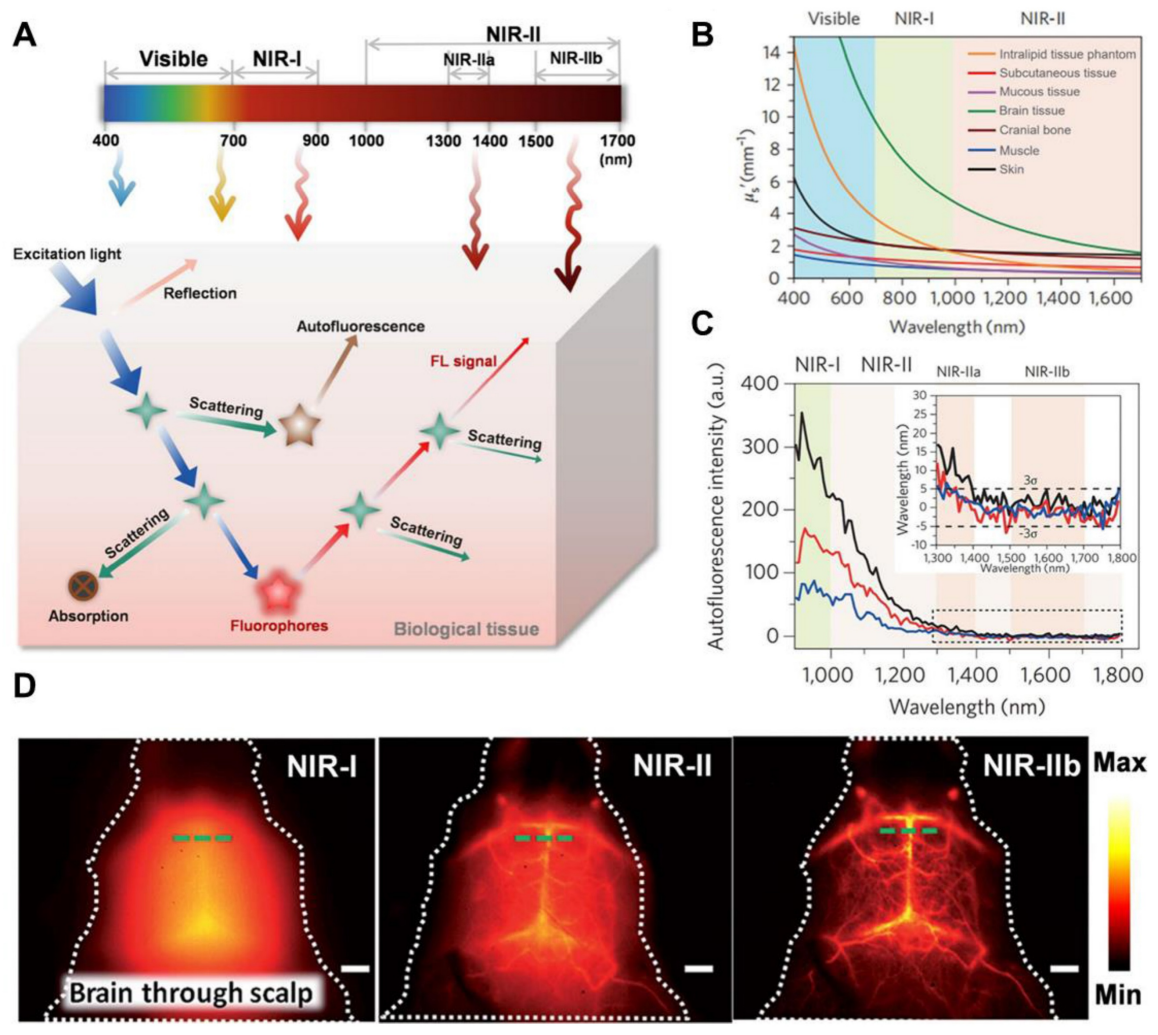
** (A)** The comparison of the interaction of light with body tissues from visible to NIR-IIb range. Adopted with permission from 20, Copyrights 2024 ACS PUBLICATIONS. **(B)** Demonstration of lower scattering coefficients in various tissues across the 400-1700 nm range. **(C)** *Ex vivo* autofluorescence spectra of different body organs of mice like liver (black), spleen (red), and heart tissue (blue) upon 808 nm irradiation demonstrating almost zero autofluorescence in the NIR-IIb range. Adopted with permission from 21, Copyrights 2017 NATURE. **(D)** Cerebrovascular Fluorescence imaging of mice demonstrating the resolution comparison among NIR-I, NIR-II, and NIR-IIb (Scale bars: 2mm). Adopted with permission from 22, Copyrights 2015 WILEY.

GBM can be visualized and distinguished using visible or digitally processed near-infrared (NIR) FL imaging generated by the radiative shift from excited states (S_1_) to ground states (S_0_) of fluorophores through competitive energy dissipation upon light irradiation 14. NIR light is preferred over visible light for efficient diagnosis and tumor visualization because excitation and emission in biologically transparent NIR windows exhibit reduced light attenuation, scattering, and autofluorescence by tissues. This leads to enhanced tissue penetration and improved signal-to-background ratios (SBR) 15. In particular, imaging in the NIR-II region (1000-1700 nm), using an indium gallium arsenide (InGaAs) camera, provided deeper infiltration up to 3 mm, higher SBR, better spatiotemporal resolution, and a safer power limit compared to the NIR-I region (780-900 nm), which offered a penetration depth of up to 0.2 mm 16. This reduced signal interference from biological tissues has made NIR-II imaging the ultimate diagnostic tool for GBM. Consequently, a growing number of NIR-II fluorescent inorganic hybrid materials and organic dyes have recently gained attention in brain tumor imaging research 199. For example, Welsher *et al.* in 2009 demonstrated NIR-II preclinical FL imaging of the mice by employing hydrophilic polymer-coated single-walled carbon nanotubes (SWCNTs) for the first time *via* the InGaAs camera 17. In 2022, the *in vivo* imaging in the 1700-2000 nm range was performed and the NIR-II definition was further refined to the 1000-3000 nm range, greatly overlapped with the short-wave infrared range (900-3000) 18. Subsequently, imaging spatial resolution, imaging depth, and SBR were greatly improved and tissue autofluorescence was greatly reduced by employing NIR-II over NIR-I FL imaging **(Figure 1)** 19.

The inorganic/hybrid fluorescent nanoprobes in the NIR-II window, like quantum dots (QDs) 23, rare earth-doped nanoparticles (RENPs) 24, carbon-based nanomaterials, and metal nanoclusters (MNCs) have also flourished in this field for noninvasive *in vivo* imaging of GBM 25,26. In comparison to other NIR-II fluorophores, inorganic/hybrid nanoprobes possess several distinct characteristics: (1) Inorganic fluorescent materials are chemically, thermally, and photostable, maintaining their structure and function under prolonged NIR-II irradiation or during storage ensuring reliable performance for both imaging and therapeutic applications in GBM treatment 27. (2) Inorganic materials exhibit strong absorption, sharp emission, and high quantum yield (Φ) in the NIR-II window, enabling deeper tissue penetration and high-resolution imaging. (3) In addition, inorganic materials possess excellent photothermal conversion efficiency and reactive oxygen species (ROS) generation capacity, making them highly effective for therapeutic applications 28. (4) Moreover, doping or modifying the composition of inorganic materials allows fine-tuning of their optical, magnetic, and therapeutic properties making them highly multifunctional and serving as MRI contrast agents, targeted drug delivery systems, and NIR-II imaging agents for optimized GBM treatment. (5) Finally, inorganic materials are resilient to the hypoxic, acidic, or oxidative conditions often present in the GBM microenvironment, ensuring consistent efficacy. Furthermore, alternative therapies must integrate features such as low toxicity, efficient BBB/blood tumor barrier (BBT) crossing, optimal biocompatibility, and mild exogenous/endogenous stimulation 29. To address this challenge, researchers have designed FL imaging-guided therapies in which inorganic material-based FL imaging diagnostics are equipped with advanced therapeutic modalities such as chemotherapy (CT) 30, photothermal therapy (PTT) 31, photodynamic therapy (PDT) 32, chemodynamic therapy (CDT) 33, sonodynamic therapy (SDT) 34, and immunotherapy for the early diagnosis and treatment of GBM 23,35.

To excel in the efficient diagnostic ability of NIR-II light and the therapeutic capability of advanced therapies, scientists have engineered NIR-II-guided theranostics capable of executing NIR-II FL imaging-guided therapy of the GBM. For example, Ren *et al.* in 2021 reviewed the recent advancements in inorganic- and organic-based NIR-II fluorescent nanoprobes and their applications in cerebrovascular angiography and brain diseases, such as traumatic brain injury, ischemic stroke, and brain tumors, employing different imaging modalities, including macroscopy, microscopy, and mesoscopy 36. Li* et al.* in 2022 summarized the latest advancements in different organic/inorganic nanohybrids for biomedical applications in the NIR-II region, including tumor imaging, inflammation tracking, and biomolecule detection 28. Mondal *et al.* in 2023 reviewed the latest progress in dyes and biomarker-mediated GBM detection along with their sensing mechanisms helping to develop next-generation smart fluorescent probes to be applied in FL-guided surgery 37. Moreover, Bian *et al.* in 2023 have comprehensively summarized the recent advancements in precise diagnostics and efficient therapeutics of GBM and analyzed the designing methodologies for imaging the BBB-crossing of the therapeutic nanoplatforms 38. The significant achievements of different imaging probes and various nanocarrier delivery systems in GBM diagnosis and treatment have also been highlighted. Furthermore, Schmidt *et al.* in 2024 have also summarized preclinical NIR-II FL imaging in animal models and the clinical translatability of emerging small-molecule NIR-II fluorescent nanoprobes for human imaging 39. The authors have emphasized the ability of small-molecule NIR-II fluorescent nanoprobes to provide high-resolution deep tissue imaging, together with enhanced precision in tumor targeting and specificity while maintaining rapid clearance from the body. The latest techniques for experimentation and translation of NIR-II FL imaging have been studied for extensive applications in the biomedical realm 40,200. The reviewers have extensively discussed the significance and applications of organic NIR-II fluorescent probes in NIR-II FL imaging-guided therapies of GBM. However, very few reviews about inorganic and hybrid NIR-II fluorescent probes have suggested a pressing need for a detailed discussion about the recent progress in this context for NIR-II FL imaging-guided therapies of GBM.

So far, inorganic/hybrid NIR-II fluorescent nanoprobes have demonstrated a significant role in the diagnostics and therapeutics of GBM. For example, Su *et al.* in 2024 engineered a hybrid X-ray-mediated nanomedicine for precise visualization of structural features of the microvasculature of glioma to define the tumor boundary for enhanced synergistic chemo-radiotherapy 41. The nanomedicine consisted of downconversion nanoparticles (DCNPs) covered with an X-ray-responsive **poly(Se-Se/DOX-co-acrylic acid)** layer and functionalized with Angiopep-2 peptide, forming **DCNP@P(Se-DOX)@ANG** targeting nanoprobe. The ultrasmall size and incorporation of chemodrug allowed it to cross the BBB effectively, enabling precise monitoring and localization of GBM *via* brain NIR-II FL imaging. The nanomedicine facilitated synergistic anti-GBM treatment by combining X-ray-induced DOX release with radio-sensitization. The adopted approach presented a novel and efficient technique for GBM imaging and combined chemo-radiotherapy. Moreover, Ge *et al.* in 2024 also designed **Bi-Ag_2_S** nanoclusters (NCs) with a high absolute FL efficiency of approximately 13.3%, due to its 1.03 Å ionic radius closely matching that of Ag^+^ 42. The Bi-Ag_2_S NCs demonstrated superior FL and deeper tissue penetration of about 5-6 nm compared to the clinical standard indocyanine green. When conjugated with lactoferrin, the NCs gained the capability of crossing the BBB and specifically targeting gliomas. Time-dependent NIR-II FL imaging revealed their efficient accumulation in gliomas after *i.v.* injection, even with the skull and scalp intact. Additionally, the NCs, free of toxic metals, exhibited minimal toxicity and excellent biocompatibility. Therefore, the recent research should also be reviewed to provide the progress in designing novel inorganic and hybrid nanomaterial-based NIR-II fluorescent nanoprobes and their applications in the diagnosis and treatment of GBM.

**Scheme 1:**
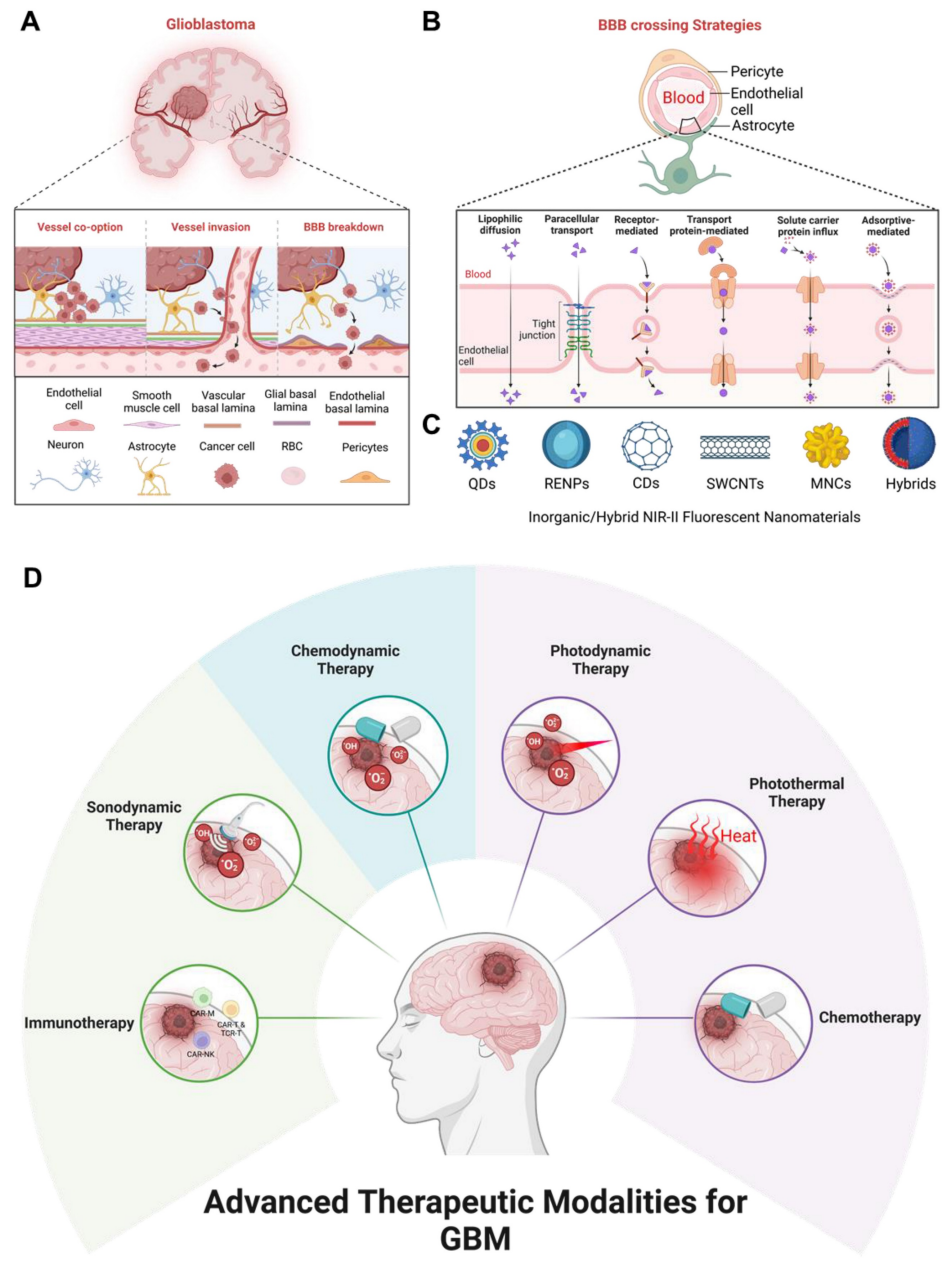
Schematic illustration of: **(A)** Physiopathology of GBM **(B)** BBB crossing strategies for GBM. **(C)** Inorganic/hybrid NIR-II fluorescent materials for the treatment of GBM. **(D)** Advanced therapeutic modalities for imaging-guided therapy of GBM.

Since inorganic and hybrid nanomaterial-based NIR-II fluorescent nanoprobes for GBM treatment have been developed rapidly, herein, we offer a timely update to summarize this field **(Scheme 1)**. In this review, the pathological and physiological characteristics of GBM have been summarized. Moreover, inorganic and hybrid nanomaterial-based NIR-II FL-guided modern therapies for GBM have been reviewed to summarize the advancements in this nanotechnology. Different therapeutic paradigms with NIR-II fluorescent inorganic and hybrid nanomaterials, including NIR-II FL imaging-guided chemotherapy, PTT, PDT, CDT, SDT, and immunotherapy have been discussed. Looking ahead, future advancements should focus on optimizing safe body clearance, ensuring long-term biocompatibility, enhancing BBB penetration, extending emission wavelengths beyond 1500 nm, and integrating dual imaging with immunotherapy to enhance therapeutic outcomes. Additionally, AI-driven strategies hold great potential to refine precision and accelerate the clinical translation of these inorganic/hybrid smart theranostic platforms for GBM treatment.

## 2. Pathological characteristics of GBM

The intricate and invasive characteristics of GBM make it nearly impossible to eliminate at the cellular level. Additionally, the presence of extensive hypoxic regions creates perivascular niches that support glioma-initiating cells. These regenerative cells can lead to the development of more aggressive recurrent tumors that exhibit resistance to both radiation and chemotherapy after surgical removal 43. Therefore, it is necessary to understand the pathological characteristics of GBM for efficient treatment and better therapeutic outcomes.

### 2.1. Signaling pathways

The increasing evidence underscored the critical role of different signaling pathways in the progression and pathogenesis of GBM, including the epidermal growth factor receptor, WNT/β-catenin, fibroblast growth factor receptor, and the PI3K/AKT/mTOR pathways 44. For instance, Boso *et al.* in 2019 identified the role of the HIF-1α/WNT pathway in the neuronal differentiation of GBM stem cells 45. Similarly, Portela *et al.* in 2019 reported that the WNT pathway triggers the JNK/MMP loop pathway, further driving GBM malignance 46. Additionally, another study emphasized the therapeutic potential of targeting the PI3K/AKT/mTOR pathway, showing that stimulation of the POU2F2-PDPK1 axis promotes oncogenesis *via* glycolysis and PI3K/AKT/mTOR pathway 47. Therefore, different signaling pathways have been discussed in this section to demonstrate their role in GBM progression.

**Figure 2:**
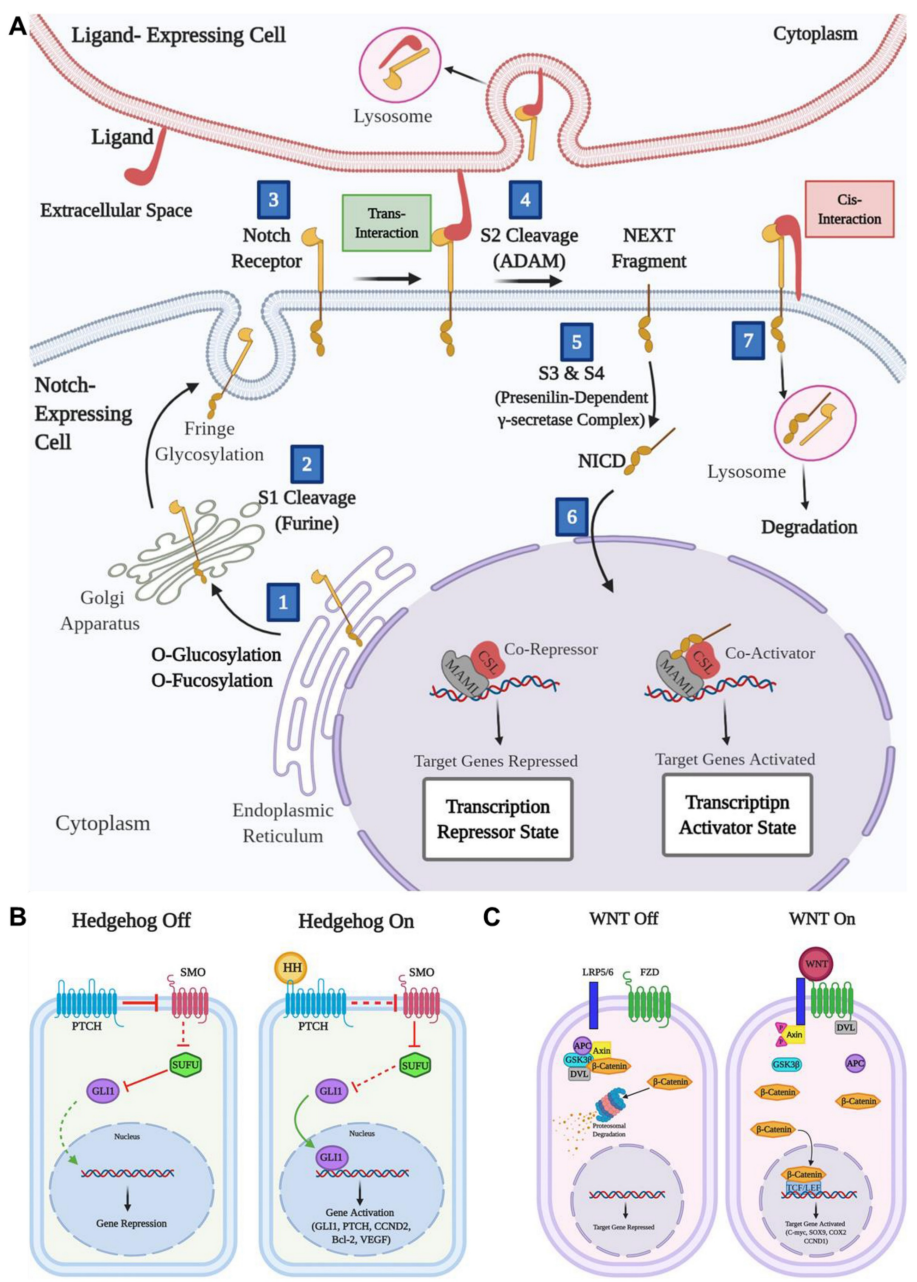
** (A)** Graphical representation of normal functioning of Notch signaling pathway and its importance in controlling cellular and developmental processes. **(B)** Schematic illustration of Hedgehog pathway: The left side demonstrates inactivation of the HH signaling pathway without HH ligand. The right side demonstrates the active HH signaling pathway after binding with the HH ligand. **(C)** Schematic representation of WNT pathway: The left side demonstrates WNT pathway deactivation in the absence of WNT ligands. The right side demonstrates activation of the WNT signaling pathway when the WNT ligand binds to the FZD receptor. Adopted with permission from 59, Copyrights 2022 ELSEVIER.

***The Notch signaling pathway*** is linked to various tumors, including GBM, where its dysregulation promotes tumor aggressiveness **(Figure 2A)** 48-50. High expression of Notch receptors (Notch 1 and 4) and components like HES-related protein (Hey1) and Delta-like ligand (DII1) are associated with worse GBM outcomes, such as reduced survival and higher tumor grades 51,52. However, some studies reported low Notch 1 and 2 expressions in certain cases, highlighting variability across GBM subtypes. Epigenetic roles of Notch signaling in GBM are underexplored, though Hey1 methylation has been identified as a potential prognostic marker. Histone deacetylase (HDAC) inhibitors like sodium butyrate reduce Hey1 expression, induce GBM cell apoptosis, and enhance DNA (cytosine-5)-methyltransferase 1 levels. Hey1 knockdown further reduces tumor aggressiveness 53. Additionally, the Delta/Notch-like epidermal growth factor-related receptor limits GBM neurosphere growth through HDAC inhibition 54. Despite these insights, further research is necessary to completely understand the epigenetic role and therapeutic abilities of Notch signaling in GBM.

The upregulation of the ***hedgehog (HH) signaling pathway*** is closely linked to gliomas, responsible for their proliferation and progression by promoting cancer stem cell activity **(Figure 2B)** 55. The sonic hedgehog (SHH) ligand is greatly expressed in brain tumors and the surrounding tissues. Additionally, a truncated isoform of tGLI1 has been strongly correlated with the aggressiveness of GBM, being expressed specifically by GBM cells only 56. Inhibition of the HH signaling pathway has been shown to reduce glioma cell migration and invasion. Bromodomain-containing protein 4 (BRD4) serves as a key regulator of GLI1 transcription by binding straight to its promoter, while lysine acetyltransferase levels are associated with the expression of HH target genes 57. The HH signaling pathway is crucial for the growth and malignance of GBM, particularly through its role in maintaining cancer stem cells, making it a significant target for GBM treatment. Research by Malatesta *et al.* emphasized the role of epigenetic modulators in increasing tumor progression by disrupting the HH signaling pathway. Their focus has been on epigenetic decoder proteins, particularly the histone acetyltransferase P300/CBP-associated factor, which directly affects the HH pathway to regulate cell propagation and tumor initiation and progression 58. Consequently, aiming the HH signaling pathway could offer a promising therapeutic strategy for GBM.

The anomalous activation of the ***wingless (WNT) signaling pathway*** is critical for cancer stem cells across various tumors, including breast, skin, bladder, colon, and blood cancers **(Figure 2C)**. This pathway regulates processes such as stem cell maintenance and therapy resistance 60. However, unlike many other cancers, constitutive initiation of the WNT signaling pathway is rare in gliomas. Despite this, Sandberg *et al.* have identified the WNT pathway as playing an essential role in the dysregulated signaling cascades of glioma stem cells 61. Alterations in the WNT signaling pathway distinguish cancer cells from healthy cells, with β-catenin and its related transcription factor TCF4 being highly translated in tumor cells with respect to surrounding brain cells 62. Some WNT signaling pathway stimulators, such as TCF4 and SOX, are significantly enhanced in high-grade gliomas. The WNT/β-catenin signaling pathway is connected to oncogenic processes in GBM, including cell propagation, inhibition of malignance, and suppression of apoptosis 63. Other components of the WNT pathway, such as DKK1, FZD1, and LEF1, are highly translated in gliomas and are associated with inefficient clinical results 64.

Transformed WNT/β-catenin signaling is linked to the destructive behavior of glioma cell lines and is associated with resistance to chemotherapy and radiotherapy 65. Studies have also demonstrated that WNT translation correlates with enhanced GBM malignance and reduced prognosis. Notably, WNT factors such as LEF1 and HOXA13 enhance cell migration and glioma development, particularly in high-grade gliomas 66,67. Moreover, WNT inhibitory factor 1 (WIF-1), which blocks WNT ligand binding to the FZD/LRP receptor, is downregulated in approximately 40% of GBM patients 68. Loss of WIF-1 has been shown to increase tumor invasion by mediating the activity of the metastasis-associated lung adenocarcinoma transcript-1 (MALAT1) 69. Other WNT pathway inhibitors, such as DKK1 and secreted FZD-related proteins, are also poorly expressed in GBM, further contributing to tumor progression 70.

### 2.2. Immunosuppressive microenvironment

The immunosuppressive mechanisms of GBM involve both intrinsic and extrinsic factors. Intrinsically, GBM tumors downregulate their antigens, contributing to their classification as “cold tumors.” These tumors are characterized by reduced levels of active T cell infiltration due to factors such as insufficient tumor antigens, ineffective presentation of "non-self" antigens by antigen-presenting cells, and the failure to activate T cells 71. Extrinsically, GBM induces immunosuppression through mechanisms such as enhanced immune checkpoints on penetrating lymphocytes and myeloid cells, secretion of suppressive factors by GBM cells, and the penetration of numerous immunosuppressive cells within the tumor microenvironment (TME) 72. These immunosuppressive agents are primary inhibitors of T cell function. GBM cells release large amounts of inhibitory molecules, including gangliosides, kynurenine, TGF-β, and VEGF, which suppress functional T cells and enhance tumor growth. Additionally, immunosuppressive cells such as Tregs and myeloid-derived suppressor cells, recruited within TME, release further inhibitory factors like TGF-β and IL-10, enhancing the suppressive conditions 73. Other contributing factors include unconventional lymphatic drainage, the relatively enclosed brain environment, and the presence of immune response antagonists, which exacerbate GBM-associated immunosuppression. Moreover, the immune-privileged status of the central nervous system (CNS), due to the BBB, the suppression of GBM antigens, and the restricted entrance of tumor-inhibiting immune cells within the tumor site, combined with the widespread presence of inhibitory factors, all support this suppressive environment 74. The immunosuppressive microenvironment of GBM remains a significant obstacle in developing effective immunotherapies, making it a critical area of ongoing research.

### 2.3. Intertumoral and intratumoral heterogeneity

The significant intertumoral and intratumoral heterogeneity of GBM has posed challenges to the progress of targeted therapeutic modalities. The Cancer Genome Atlas (TCGA) formerly classified GBM into 4 molecular subtypes based on genetic and epigenetic markers. The GBM types include mesenchymal, classical, proneural, and neural 75,76. The mesenchymal subtype is marked by transmutations in the NF1 tumor-inhibiting gene and recurrent transmutations in PTEN and TP53 tumor-inhibiting genes. The classical type is extremely malignant and differentiated by EGFR augmentation but notably lacks TP53 mutations 77. Proneural type of GBM often involves TP53 mutations and is uniquely associated with IDH1 and PDGFRA transmutations. In contrast, the neural type expresses several genes commonly found in noncancerous neurons of the brain. However, a subsequent transcriptome analysis proposed a three-subtype model-proneural, classical, and mesenchymal suggesting the neural subtype may result from the adulteration of samples with non-tumor cells 78. Among these subtypes, mesenchymal and classical GBMs are typically more invasive, while proneural tumors are less invasive and more frequently observed in young individuals. Resistance to therapy has been linked to a proneural-to-mesenchymal transition. Although the TCGA classification divides GBMs into four distinct subgroups, recent research indicates that spatial and temporal variability exists within individual tumors 79. Single-cell RNA sequencing studies by Patel *et al.* revealed that a single tumor has the ability to harbor a diverse population of defective cells encompassing all GBM types, highlighting the complexity and heterogeneity of these tumors 80.

### 2.4. Blood Brain Barrier (BBB)

The BBB acts as a defensive interface between the cardiovascular system and the extracellular space of the CNS 81. It is primarily composed of endothelial cells forming a tightly regulated barrier along blood vessel walls, selectively restricting the entry of compounds into the brain parenchyma. A major challenge in GBM treatment is the delivery of chemotherapeutic drugs across the BBB 82. Tight junctions, measuring less than 1 nm, prevent the penetration of over 98% of small molecules 83. In GBM, the BBB exhibits increased permeability due to the presence of poorly formed, leaky blood vessels, upregulated transporter proteins, and reduced expression of tight junction proteins. However, this interruption is inconsistent across the tumor, with some regions having highly permeable blood vessels and others maintaining more intact vasculature 84. Even when a chemodrug crosses into the tumor cells, achieving therapeutic levels within tumor cells is often hindered by the upregulation of efflux pumps by GBM cells. As a result, significant portions of GBM retain areas with an intact BBB and elevated efflux pump activity, further complicating effective drug delivery 85. Therefore, researchers have designed the materials capable of crossing the BBB to reach the tumor area for accurate diagnosis and enhanced therapeutic outcomes by critically modulating the surface chemistry and size of the nanoparticles. The inorganic and hybrid nanomaterials capable of crossing BBB are mentioned in the next section highlighting the importance of inorganic/hybrid NIR-II fluorescent nanoprobes in GBM treatment *via* modern therapies to achieve higher survival rates and better patient healthcare.

## 3. NIR-II emissive inorganic/hybrid nanomaterials

NIR-II fluorescent inorganic and hybrid nanomaterials include a diverse range of materials that exhibit strong and tunable FL in the NIR-II region 40. QDs, RENPs, and MNCs are commonly used for their deep tissue penetration, high quantum efficiency, stability, precise optical properties, and rapid clearance 23-25. Carbon-based nanomaterials, such as SWCNTs and CDs, also show promising NIR-II emissions, combining biocompatibility with excellent optical characteristics 86,87. Hybrid materials, incorporating organic and inorganic components, can enhance biocompatibility, improve functionalization for specific targeting, and increase the efficiency of imaging and therapeutic applications. In this section, the formation, structure, and FL properties of inorganic and hybrid fluorescent materials have been discussed to demonstrate their importance in imaging-guided modern therapies of GBM.

### 3.1. QDs-based NIR-II fluorescent probes

**Figure 3:**
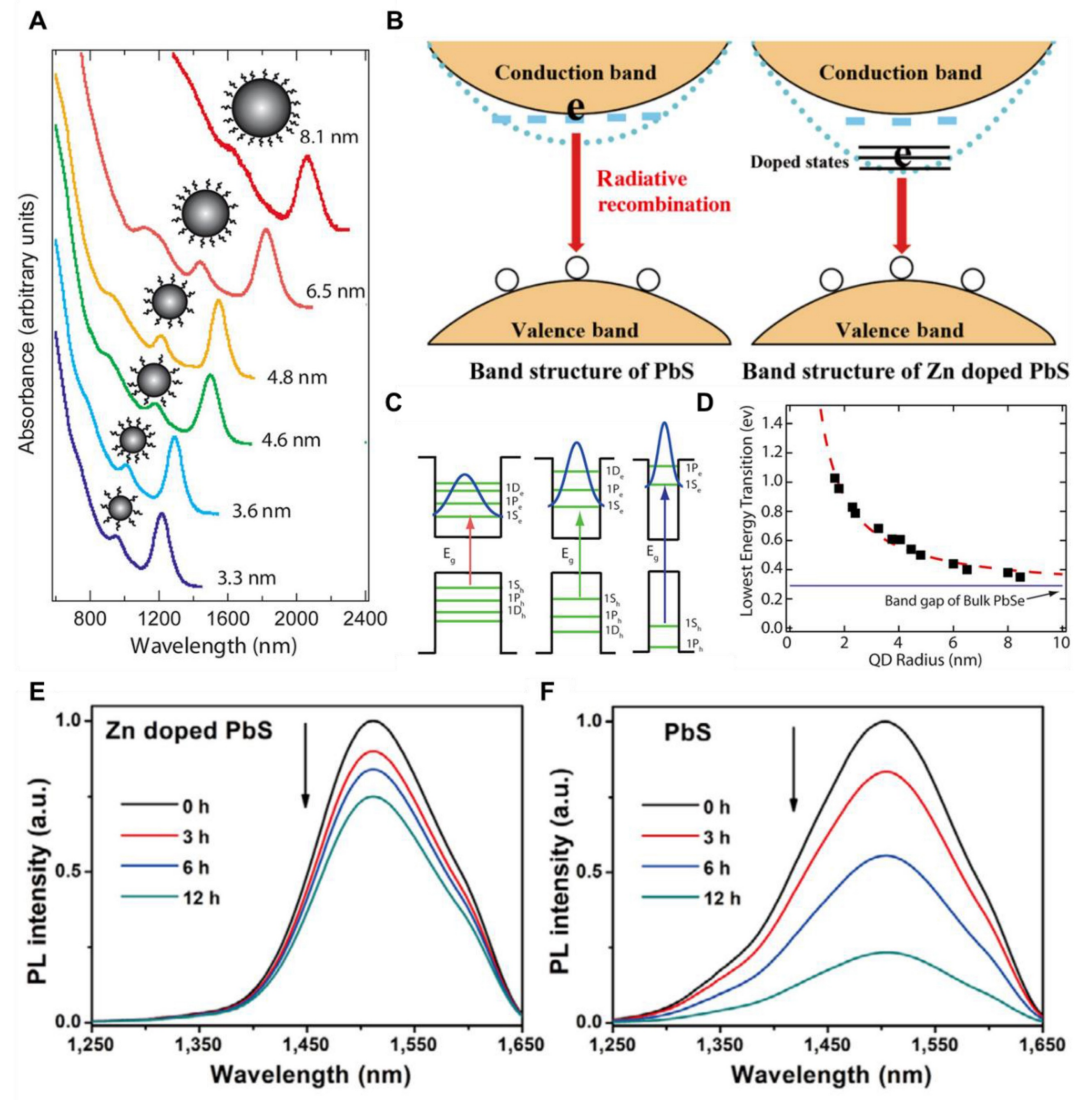
** (A)** Size-dependent absorption spectra of QDs demonstrating quantum confined 1^st^ exciton-mediated absorption shift. **(B)** Demonstration of composition-dependent change in the bandgap of the QDs. **(C)** Demonstration of discrete transitions representing the discrete exciton transitions.** (D)** Representation of increased quantum confinement of the 1^st^ exciton by decreasing the size of the particle. **(E-F)** FL spectra of the QDs after exposure to air at 80 °C, representing the composition-dependent FL stability of the QDs. (B), (E), and (F) are adopted with permission from 90, Copyrights 2020 SPRINGER NATURE. (A), (C), and (D) are reused under Creative Commons Attribution License 91.

QDs, typically ranging from 2 to 10 nm in size, are semiconductor nanoparticles composed of **CdSe, CdTe, Ag_2_S, or InP** possessing spherical core-shell configuration surrounded by a protective shell made up of **ZnS** 88,89. The shell enhances the fluorescence intensity and photostability of the QDs, enabling their long-term use in various environments. Compared to traditional organic dyes, QDs exhibit superior optical properties, including narrow emission spectra and broad absorption spectra for high fluorescence intensity. The defining feature of their nanoscale behavior is the band gap representing the energy gap between the VB and CB in QDs which lies at the core of their unique optical properties **(Figure 3B)** 90,91, 181. When QDs are illuminated with photons possessing energy higher than this bandgap, an electron in the VB transitions to the CB, leaving behind a positively charged hole creating an electron-hole pair, which represents a temporary exciton generation. The exciton formation and its subsequent relaxation pathways underpin the fundamental working mechanism of QDs. The relaxation of the exciton occurs *via* two principal pathways: radiative and non-radiative 92. In radiative relaxation, the electron recombines with the hole, releasing energy in the form of a photon depending on the size and configuration of the QD, with smaller QDs exhibiting a larger bandgap and emitting shorter wavelengths while larger QDs have a smaller bandgap and emitting longer wavelengths **(Figure 3C)** 93. This size-dependent tunability of FL is a hallmark property of QDs, making them highly valuable for applications such as multiplexed imaging, biosensing, and FL-guided diagnostics **(Figure 3A)** 94. In contrast, non-radiative relaxation involves the dissipation of the excess energy of electrons as heat rather than light, less favorable for FL-based applications. The heat generated can be harnessed in PPT, where localized heating is used to selectively destroy cancer cells. This duality in relaxation pathways, with the possibility of either light emission or heat generation, significantly broadens the utility of QDs across diverse fields. The quantum confinement, which arises when the dimensions of the QD are lesser than the exciton Bohr radius, plays a pivotal role in these processes **(Figure 3D)**. This effect directly impacts the bandgap energy and enables precise control over the optical properties of QDs through adjustments in their size, shape, and composition **(Figure 3E and F)**. The interaction of QDs with light, governed by this mechanism, enables their application in a wide range of scientific and biomedical domains, including diagnostics and targeted therapies 95.

**Figure 4:**
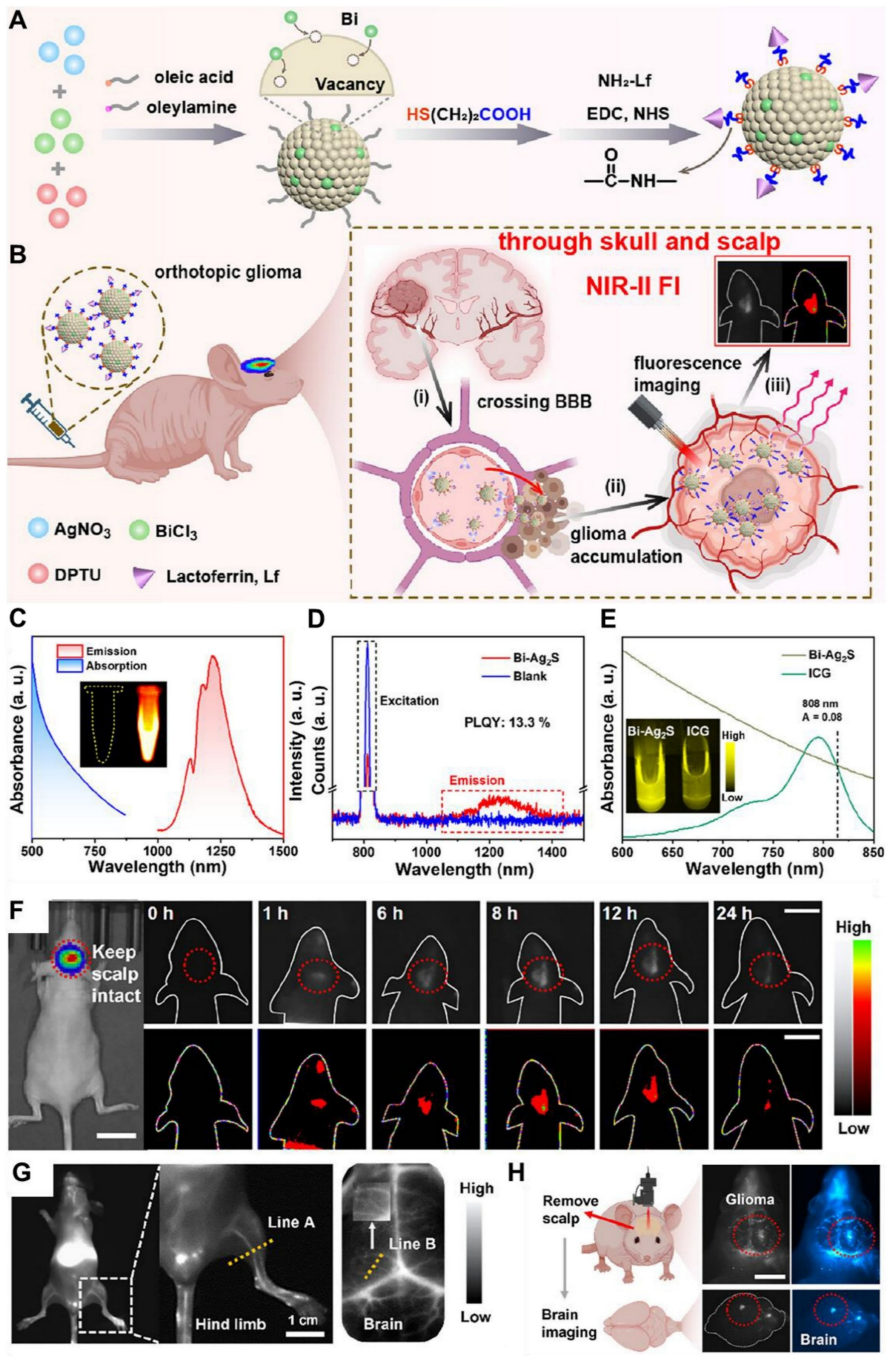
Schematic demonstration of **(A)** Lactoferrin functionalized Bi-Doped Ag_2_S NCs and their **(B)** application in NIR-II imaging of orthotopic glioma. **(C)** Absorption and FL spectra of the NCs in chloroform and the inset showing the FL image captured *via* a near-infrared camera upon 808 nm laser excitation and 100 ms exposure time with LP1000 filter. **(D)** Measurement of the absolute Φ of the NCs in chloroform. **(E)** Absorption spectra and NIR-II imaging comparison of the NCs in chloroform and ICG in DMSO under 808 nm laser excitation. **(F)** NIR-II imaging of orthotopic glioma-bearing mice following the injection of sample NCs upon 200 ms exposure time with LP1000 filter. **(G)** NIR-II imaging of hind-limb and cerebral blood vessels following *i.v.* administration of sample NCs (scale bar: 1 cm). **(H)** Schematic representation of FL images of the exposed brain and *ex vivo* brain tissue 24 hours post-injection upon 400 ms exposure time with LP1000 filter (scale bar: 0.5 cm). Adopted with permission from 203, Copyrights 2024 ACS PUBLICATIONS.

NIR-II QDs are especially valuable for *in vivo* imaging and phototherapy because of their tunable optical properties, smaller size, and high FL stability, which allow for precision targeting of diseased areas and higher cell-penetrating ability. For example, Ge *et al.* engineered bismuth (Bi) doped Ag_2_S nanocrystals (NCs) because of the non-toxic nature of Bi and its optimal ionic radius (1.03 Å), which closely matches that of Ag⁺ among commonly available non-toxic alternatives. The Bi-doped Ag_2_S NCs were synthesized through a simple process, exhibiting an emission peak around 1230 nm and an absolute Φ of approximately 13.3% **(Figure 4C-E)**. The designed NCs demonstrated higher brightness and deeper tissue penetration of 5-6 mm in an intralipid-based tissue model as compared to ICG. To enhance glioma targeting and facilitate BBB crossing *via* receptor-mediated transport (RMT), lactoferrin was conjugated onto the NCs **(Figure 4A and B)**. Remarkably, after intravenous administration, the Lf-functionalized NCs showed stable accumulation in orthotopic glioma for NIR-II imaging, even with the skull and scalp intact, and demonstrated penetration up to 3 mm **(Figure 4F-H)**. Moreover, both *in vitro* and *in vivo* evaluations confirmed their low toxicity and excellent biocompatibility, highlighting their potential for clinical glioma diagnosis.

Therefore, QDs are uniquely capable of crossing the BBB, a significant obstacle for most conventional therapies of GBM, and delivering anticancer drugs directly to the brain. Their ability to specifically target cancer cells with minimal toxicity to surrounding healthy tissue underscores their potential in precision medicine 96. Moreover, their biocompatible surfaces can be functionalized with biomolecules through covalent coupling or electrostatic interactions, further enhancing their targeting capabilities in molecular imaging and drug delivery systems. For example, Li *et al.* designed an ultrasmall Nd^3+^-coordinated black phosphorus DQs, exhibiting enhanced NIR-II FL imaging capabilities and X-ray-induced PDT. The sample was specifically designed to target glioma cells, enabling NIR-II FL emission for monitoring GBM intracranial diffusion while simultaneously delivering synergistic X-ray-induced chemo/PDT to hinder GBM progression. The prepared QDs efficiently absorbed NIR photons and transferred the energy to Nd^3+^ ions, facilitating electronic transitions and FL emission. Simultaneously, the high atomic number of Nd also enhanced X-ray attenuation and redirected low-energy photons to the QDs, boosting ROS generation. Additionally, the chemotherapeutic drug doxorubicin (DOX) and ROS-sensitive polymer-grafted cyclic RGD peptides were co-anchored on the sample surface to improve GBM targeting and enhance phototheranostics efficacy 201. However, many QDs contain heavy metals that can pose toxicity risks and may require biomimetic protective coatings or alternative compositions to improve biocompatibility. Their long-term stability in physiological environments can also be compromised by surface oxidation and ligand detachment, affecting imaging performance. Therefore, there is a need to develop non-toxic, heavy metal-free QDs with stable FL properties for addressing the mentioned challenges.

### 3.2. RENP-based NIR-II fluorescent probes

RENPs for NIR-II-based phototheranostics can be prepared from inorganic host matrix doped with lanthanide ions (Ln^3+^) as sensitizers (Nd^3+^, Yb^3+^, and Er^3+^) or activators (Ho^3+^, Pr^3+^, Tm^3+,^ and Er^3+^) 97. Typically, RENPs consist of a nanoscale inorganic host and a specific concentration of luminescent ions, either as activators or sensitizers. Each RENP has a unique set of energy levels, leading to characteristic sharp emission bands. The intensity and spectral profile of these emissions are significantly influenced by the host material and the type of metal dopants. To minimize non-radiative energy loss, a transparent host matrix with low phonon energy is essential. By carefully selecting metal dopants, NIR-II emission bands can be selectively generated 98. Nd^3+^, Yb^3+^, Er^3+^, Ho^3+^, Tm^3+^, and Pr^3+^ have been identified as capable of producing NIR-II emission bands through three key mechanisms. The first involves Stokes-type emissions from Nd^3+^ or Er^3+^ under 808 nm excitation. The second mechanism is energy transfer from Yb^3+^ to an Ln^3+^ activator (Er, Ho, Tm, or Pr), forming a Yb^3+^-Ln^3+^ pair, typically excited at 980 nm. In the last mechanism, Nd^3+^ acts as the first sensitizer, transferring energy to Yb^3+^ as the second sensitizer, which then transfers energy to Ln^3+^ activators (Er, Ho, Tm, or Pr) under 808 nm excitation^99^.

**Figure 5:**
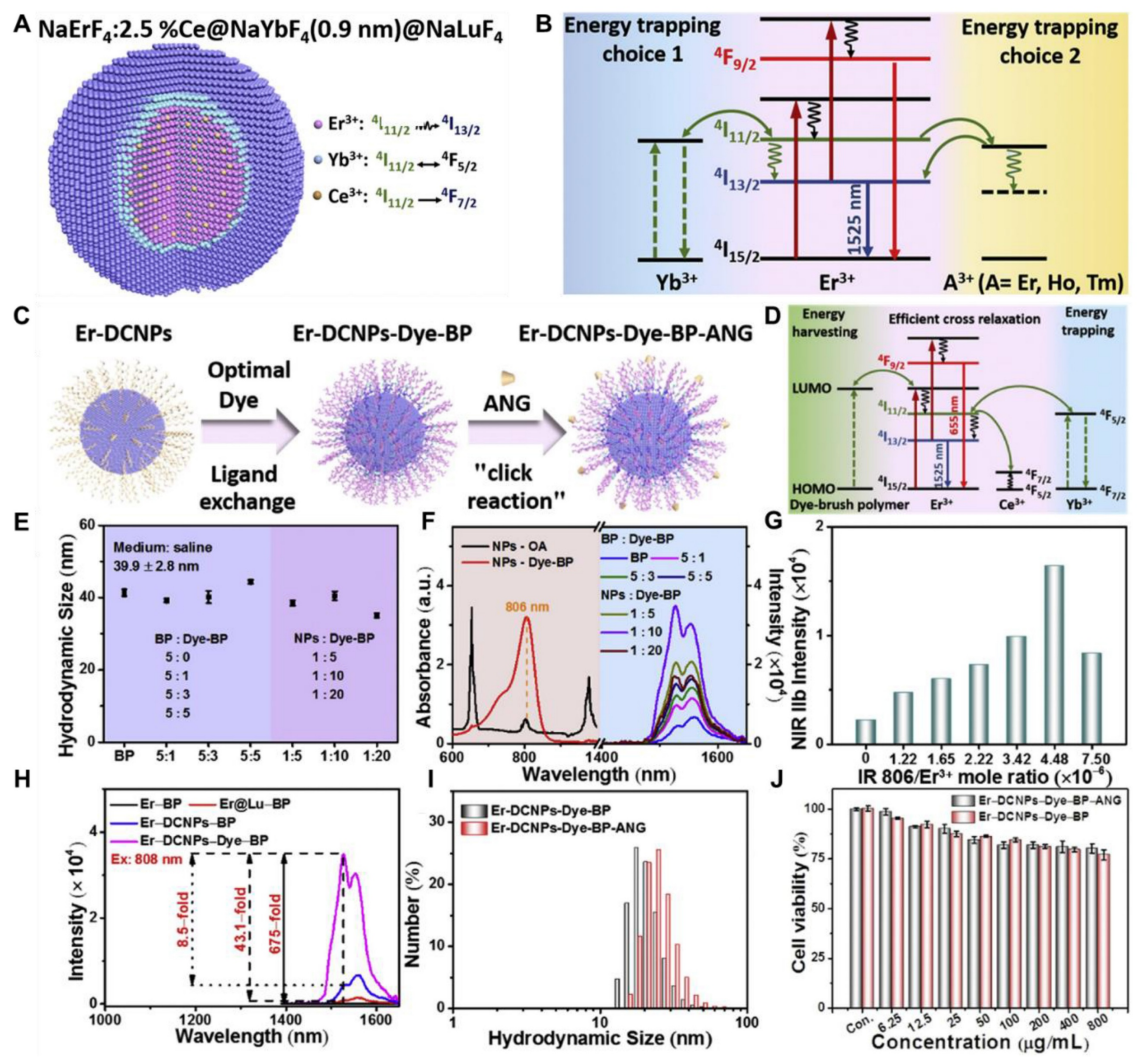
** (A)** Graphical representation of core-shell structured RENPs for NIR-II FL imaging.** (B)** The demonstration of the energy diagram of the nanoparticle representing the upconversion (655 nm) and downconversion (1525 nm) process upon 808 nm light irradiation. **(C)** Graphical representation of the synthetic mechanism of brush polymer-based-dye conjugated angiopep-2 functionalized RENPs. **(D)** The energy diagram demonstrates the transfer of energy transfer between dye and Er^3+^ ions and photon changeover among the Er^3+^, Ce^3+^, and Yb^3+^ causing 1525 nm FL. **(E)** The size of the RENPs depends on the amount of brush polymer and dye. **(F)** Composition-dependent absorption and NIR-IIb emission spectra of the RENPs upon 808 nm light irradiation. **(G)** The amounts of dye and Er^3+^ ion-dependent NIR-IIb FL intensity of the RENPs. **(H)** The NIR-IIb FL spectra of different compositions of RENPs. **(I)** The angiopep-2 dependent hydrodynamic size variations of RENPs. **(J)** Demonstration of angiopep-2 dependent biocompatibility of the RENPs for U87 cells after 24 hours of incubation. Adopted with permission from 103, Copyrights 2020 ELSEVIER.

Traditionally recognized sensitizers, Nd^3+^ and Er^3+^ are also effective activators capable of producing NIR-II luminescence through Stokes-shifting. For Nd^3+^, excitation at 740/808 nm promotes transitions from the ground ^4^I_13/2_ state to the excited ^4^F_7/2_ or ^4^S_3/2_ states, followed by multiphonon relaxation to the ^4^F_3/2_ state. Radiative decays from the ^4^F_3/2_ state result in emissions at 1060 nm (^4^F_3/2_ → ^4^I_11/2_) and 1330 nm (^4^F_3/2_ → ^4^I_13/2_) 100. Similarly, Er^3+^ undergoes upward transitions from the ^4^I_15/2_ state to the ^4^I_9/2_ or ^4^I_11/2_ states under 808/980 nm excitation, followed by multiphonon relaxation to the ^4^I_13/2_ state, resulting in a 1525 nm emission through radiative decay to the ^4^I_15/2_ ground state. Yb^3+^ exhibits energy levels ^2^F_5/2_ and ^2^F_7/2_, separated by an energy gap that corresponds to 980 nm photons. Its high absorption cross-section (1.3 × 10^-21^ cm^2^) makes it an ideal sensitizer for other Ln^3+^ ions. After absorbing energy and transitioning to the ^2^F_5/2_ state, Yb^3+^ transfers energy to Er^3+^, populating its ^4^I_11/2_ state. Relaxation to the ^4^I_13/2_ state and subsequent radiative decay to the ground state yield a 1525 nm emission **(Figure 4B)**. A similar process occurs for Pr^3+^ (1289 nm, ^1^G_4_ → ^3^H_5_) and Ho^3+^ (1155 nm, ^5^I_6_ → ^5^I_8_). The Yb^3+^-Tm^3+^ pair demonstrates two-photon upconversion, with sequential energy transfers populating the ^3^F_2_ and ^3^F_3_ states of Tm^3+^ 101. Nonradiative relaxation to the ^4^H_4_ state leads to 1475 nm emission *via* radiative decay (^3^H_4_ → ^3^F_4_). Nd^3+^ also functions as a sensitizer for Yb^3+^. Upon 808 nm excitation, Nd^3+^ transitions to the ^3^F_3/2_ state and transfers energy to Yb^3+^ through ^4^F_3/2_ (Nd^3+^) + ^2^F_7/2_ (Yb^3+^) → ^4^I_9/2_ (Nd^3+^) + ^2^F_5/2_ (Yb^3+^). This process enables the excitation wavelength to shift from 980 nm to 808 nm, facilitating Nd^3+^-Yb^3+^-Ln^3+^ energy transfers for NIR-II emissions. Er^3+^, beyond its role as an activator, can also act as a sensitizer for Er^3+^-Ln^3+^ pairs, generating upconverted NIR-II emissions under 1525 nm excitation (^4^I_15/2_ → ^4^I_13/2_ transition). Through two-photon processes, Er^3+^ ions reach the ^4^I_9/2_ state and transfer energy to other Ln^3+^ ions, such as Nd^3+^ (^4^F_5/2_), Ho^3+^ (^5^F_5_), or Tm^3+^ (^3^F_2_). Radiative decay from these states produces emissions at 1060 nm (Nd^3+^, ^4^F_3/2_ → ^4^F_5/2_), 1330 nm (Nd^3+^, ^4^F_3/2_ → ^4^_13/2_), 1155 nm (Ho^3+^, ^5^I_6_ → ^5^I_8_), and 1475 nm (Tm^3+^, ^3^H_4_ → ^4^F_4_).^102^ Based on energy-cascaded downconversion from Yb^3+^ to Er^3+^ upon excitation at 980 nm, Li *et al*. performed engineering research to construct efficient NIR-IIb Er^3+^-Ce^3+^-Yb^3+^ lanthanide nanoparticles (**NaErF_4_:Ce@NaYbF_4_@NaLuF_4_**, Er-DCNPs) for the targeted imaging and resection of orthotopic GBM **(Figure 5A)** 103. The Er-DCNPs were modified with IR-806-decorated brush polymer to realize impressively enhanced NIR-IIb emission at 1525 nm and excellent biocompatibility in water **(Figure 5J)**. Upon synergistic function of active targeting by angiopep-2 peptide (ANG) and focused ultrasound (FUS) treatment, high accumulation of the nanoprobe in small-size orthotopic glioma was achieved with an SBR up to 12.5 through intact skull and scalp and even up to 150 after cardiac perfusion and craniotomy **(Figure 5C)**. Therefore, RENPs have emerged as promising tools for various biomedical applications, particularly in imaging and therapy.

In the field of bioimaging, RENPs can be designed to emit light in the NIR-II window (1000-1700 nm), which allows for even deeper tissue penetration and higher-resolution imaging 104. This is particularly useful for visualizing deep-seated tumors and other internal organs. Moreover, RENPs serve as versatile nanoplatforms for carrying diverse functional molecules, such as photothermal agents, photosensitizers, and precursor compounds, on their surfaces. Their nanoscale size enhances their ability to accumulate in lesions. Additionally, RENPs possess exceptional optical properties that enable them to convert deeply penetrating NIR light into visible or UV light, which can then activate functional molecules for therapeutic applications 105. Furthermore, these can be functionalized with various targeting ligands to specifically deliver them to diseased tissues. Over the last decade, several RENPs-based therapeutic strategies including PTT and PDT have been developed for the treatment of GBM. Despite their strong NIR-II FL and stability, potential toxicity and long-term accumulation have raised biocompatibility concerns and require careful surface modifications for clinical applications. Similarly, their large size can hinder efficient renal clearance and can lead to prolonged retention in organs causing organ damage. Further research is needed to optimize surface coatings and explore biodegradable alternatives for improved safety and better healthcare.

### 3.3. Metal nanocluster-based NIR-II fluorescent probes

MNCs, containing of few to hundreds of metal atoms, are multiple-core aggregation particles with stable structures and are categorized as ultra-small nanoparticles (1-3 nm) with precise atomic composition and structure 106. They represent a unique material structure, bridging the gap of atoms, metal complexes, and plasmonic metal nanoparticles. MNCs are multifunctional nanomaterials, with some exhibiting FL in the NIR-II window 107. Their ultra-small size is responsible for strong electron energy quantization, transforming the continuous CB seen in metal nanoparticles into discrete energy levels characteristic of nanoclusters. This energy level spacing is crucial for the FL properties of MNCs, which can be viewed as semiconductors with notable band gaps. Generally, as the size of Au nanoclusters increases, the emission wavelength becomes longer 108. To understand the relationship between structure and optical properties, researchers have used time-dependent DFT to analyze the electronic band structure and absorption spectra. The sp atomic orbitals dominate the HOMO and the lowest three LUMO, forming the sp-band. Meanwhile, HOMO-1 to HOMO-5 primarily consists of 5d^10^ atomic orbitals of Au, forming the d-band. Different absorption peaks correspond to transitions such as intraband (sp → sp), interband (d → sp), and mixed transformation. Moreover, further FL studies revealed that the visible and NIR FL of Au nanoclusters with core-shell formation are because of the core and surface states (-SR-Au-SR-) of the metal 109. Surface ligands significantly influence FL properties through mechanisms such as (i) charge transfer between ligand and Au core *via* the Au-S bond and (ii) direct donation of delocalized electrons from ligand groups to the core 110. Therefore, selecting ligands with strong electron-donating capabilities effectively enhances the FL intensity of metal clusters. Ligand-metal charge transfer further amplifies this effect, with stronger electron-donating ligands producing more intense FL. For larger nanoparticles, FL is typically enhanced through plasmon excitation, whereas QDs derive their FL from electron transitions between the VBs and CBs. In contrast, the FL of metal clusters is governed by multiple factors influencing their band structure, making it more complex.

However, synthesizing MNCs with atomic precision remains a significant challenge due to their structural complexity. The process needs strict control over reaction conditions, including pH, temperature, time, and reducing agents 111. Two key methods for achieving precise synthesis are the size-focusing method and ligand exchange-induced size/structure transformation (LEIST). The size-focusing method relies on kinetic control, with factors such as temperature, solvent, reductants, and reactant ratios determining the outcome. Through thermodynamic selection, mixed nanoclusters are refined into monodisperse products, following a "survival of the fittest" principle. The LEIST method builds upon size-focusing, enabling the transformation of one nanocluster size into another (e.g., **Au_25_(SR)_18_** to** Au_28_(SR′)_20_** or** Au_144_(SR)_60_** to** Au_133_(SR′)_52_**) and expanding the variety of nanoclusters with improved performance and applications 112. However, challenges remain in achieving atomic precision due to the diversity of ligands and the complexity of shell structures. As NIR-II fluorescent materials, MNCs have found extensive use in modern technologies, including sensing, bioimaging, and theragnostic applications. The absorption and scattering properties of MNCs are primarily influenced by localized surface plasmon resonance and depend on factors such as size, shape, composition, and environmental conditions 113. Additionally, the FL characteristics of MNCs are affected by their size, surface structure, and ligands. Au nanoclusters, in particular, have been extensively investigated because of their ultrasmall size, longer Stokes shift, excellent biocompatibility, and low cytotoxicity. Atomic-precision Au nanoclusters, having molecular formulas of **Au_n_SR_m_**, have been successfully synthesized demonstrating no relation of FL with the number of Au atoms. For instance, as one Au atom is added, the absorption peak of Au_23_ shifts to a shorter wavelength to form Au_24_ 114. However, from Au_24_ to Au_25_, the absorption peak undergoes a red shift instead of a blue shift. As the cluster grows from Au_25_ to Au_28_, the absorption peak changes back to a shorter wavelength. These variations are closely tied to the structural arrangement of Au atoms, with the optical properties of the nanoclusters being determined by their atomic configuration.

**Figure 6:**
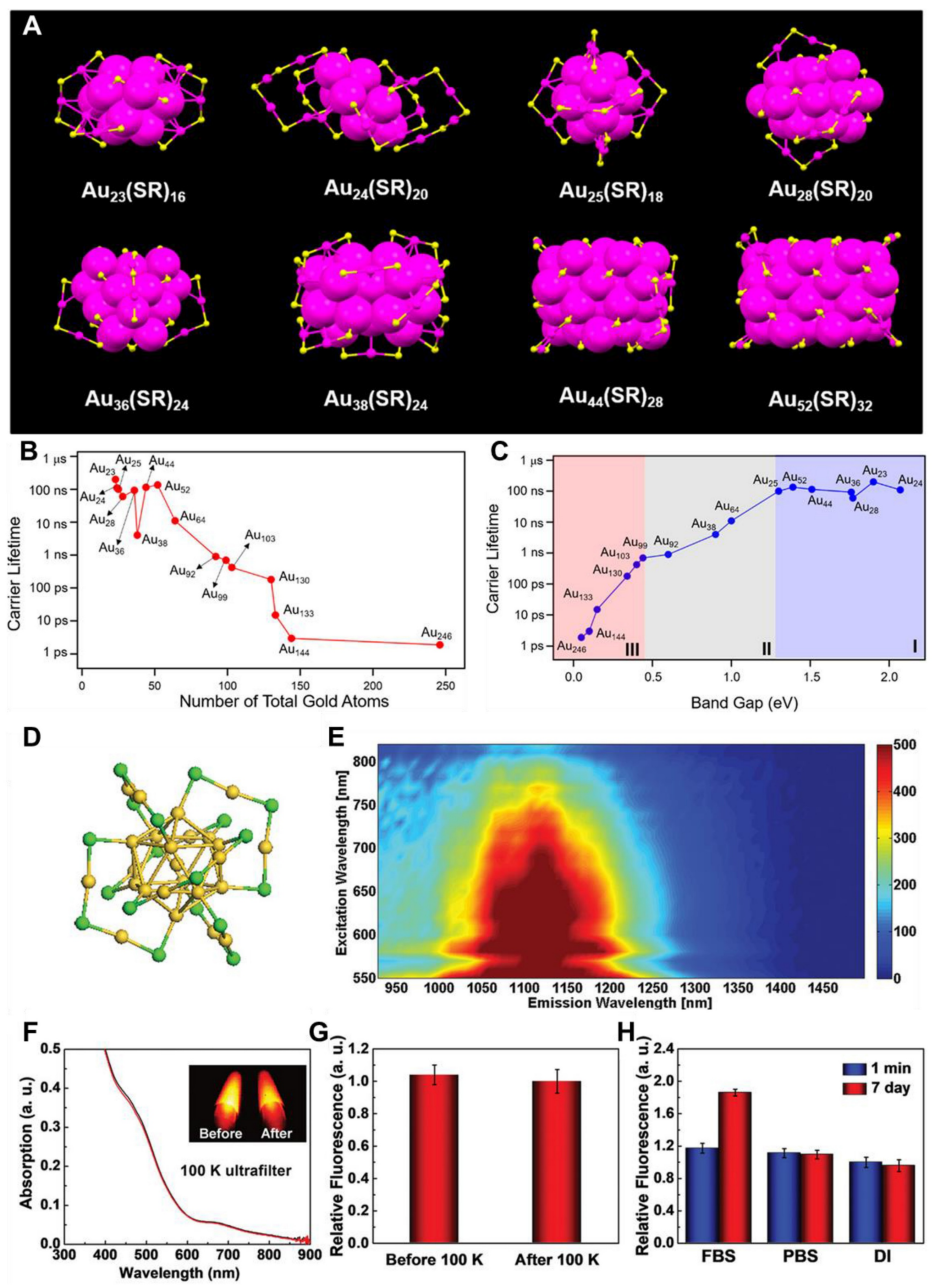
** (A)** Schematic illustration of crystal geometry of **Au_n_(SR)_m_** nanoclusters. Color coding: magenta for Au and yellow for S. **(B and C)** Graphical representation of carrier lifetime *vs* no. of Au atoms and carrier lifetime *vs* bandgap for -S^-^ protected Au nanoclusters. **(D)** The representation of the crystal geometry of Au_25_ nanocluster as core functionalized with 18 Sulfur atoms. **(E)** FL *vs* excitation spectra of Au nanoclusters demonstrating emission at 1120 nm. **(F)** UV-vis absorption and NIR-II FL imaging comparison for Au nanoclusters in water upon 808 nm excitation at power of 25 mW cm^-2^ pre- and post-filtration with the ultrafiltration tube of 100 K. **(G)** NIR-II FL intensity demonstration for Au nanoclusters pre- and post-filtration with the ultrafiltration tube of 100 K. **(H)** Demonstration of stability of Au nanoclusters in water, PBS, and FBS solutions in different time frames. Adopted with permission from 113, Copyrights 2019 ACS PUBLICATIONS. Adopted with permission from 114, Copyrights 2019 WILEY.

Moreover, for Au nanoclusters with more than 100 atoms, such as **Au_103_S_2_(SR)_41_**, **Au_130_(SR)_50_**, **Au_133_(SR)_52_**, **Au_144_(SR)_60_**, and **Au_246_(SR)_80_**, the UV-vis spectra become broader and weaker compared to nanoclusters with fewer than 100 atoms **(Figure 6)** 113. This is attributed to differences in the surface configuration of Au atoms and the SR ligands. These findings demonstrated that the absorption and scattering of Au nanoclusters are governed by their size, shape, composition, and adjacent environment. By carefully controlling these parameters, efficient NIR-II fluorescent Au nanoclusters can be designed to boost modern theranostics for better patient management. Because of their exceptional biocompatibility and photostability, MNCs could be part of clinical theranostics in the near future 112.

Silver (Ag) nanoclusters, sharing the same chemical and physical properties as Au nanoclusters, also exhibit NIR-II FL and have been utilized for biomedical imaging because of their biocompatibility and FL stability.^109^ A novel Ag-S nanocluster, **Ag_34_S_3_SBB_20_(CF_3_COO)_6_^2+^**, has been reported to discharge NIR-II FL at 1100 nm. This nanocluster was synthesized through a reaction involving a hydride- and phosphine-protected Ag nanocluster and Ag-chalcogenolate nanoclusters. The **Ag_34_S_3_SBB_20_(CF_3_COO)_6_^2+^** structure was further modified using Ag_18_, differing from formerly reported clusters. Its two characteristic optical absorption peaks were at 496 nm and 618 nm, while its FL emissions, in both liquid and solid states, are at 1100 nm under 497 nm illumination 115,116. Typically, most fluorescent Ag nanoclusters are stabilized with thiolate ligands, as silver-alkynyl clusters usually do not display fluorescent properties. Recently, it has been demonstrated that a silver-alkynyl nanocluster based on the NbO matrix can exhibit triple emission from the visible range to the NIR-II region, a significant challenge in this field 117. The FL stability and Φ of these nanoclusters can be enhanced by optimizing their spatial arrangement and orientation. The FL characteristics of SD/Ag_18_ nanoclusters were analyzed under temperature variations from 290 K to 90 K, revealing a 6-fold increase in emission intensity as the temperature decreased. Interestingly, a red shift in the emission peak was observed, moving from 630 nm to 647 nm with falling temperature. Additionally, a new peak emerged at 524 nm at 170 K, enabling triple emissions at 524 nm, 647 nm, and 1036 nm, covering the visible to NIR-II range 109.

To better understand the FL mechanism in the NIR-II region, DFT calculations using the VASP program were employed. The DOS analysis revealed a 0.5 eV band gap between the 5s states of Ag and the 2p states of C, which correlates precisely with the emission peak at 1036 nm. These findings indicated that the NIR-II emission primarily originated from transitions between the 2p states of C and the 5s states of Ag 118. Therefore, Ag nanoclusters could be potential fluorescent materials for NIR-II FL brain imaging because of their exceptional biocompatibility, and FL stability and could also be used as a theranostic agent for PTT of the GBM-like Au nanoclusters. Though MNCs enable renal clearance and strong NIR-II emission, their biocompatibility can be affected by surface ligand instability causing potential cytotoxicity and immune response risks. Moreover, their small size can lead to rapid clearance, limiting their circulation time and reducing imaging duration. Therefore, strategies like ligand engineering and controlled aggregation should be explored to enhance their stability and retention *in vivo*.

### 3.4. Single-walled carbon nanotube-based NIR-II fluorescent probes

**Figure 7:**
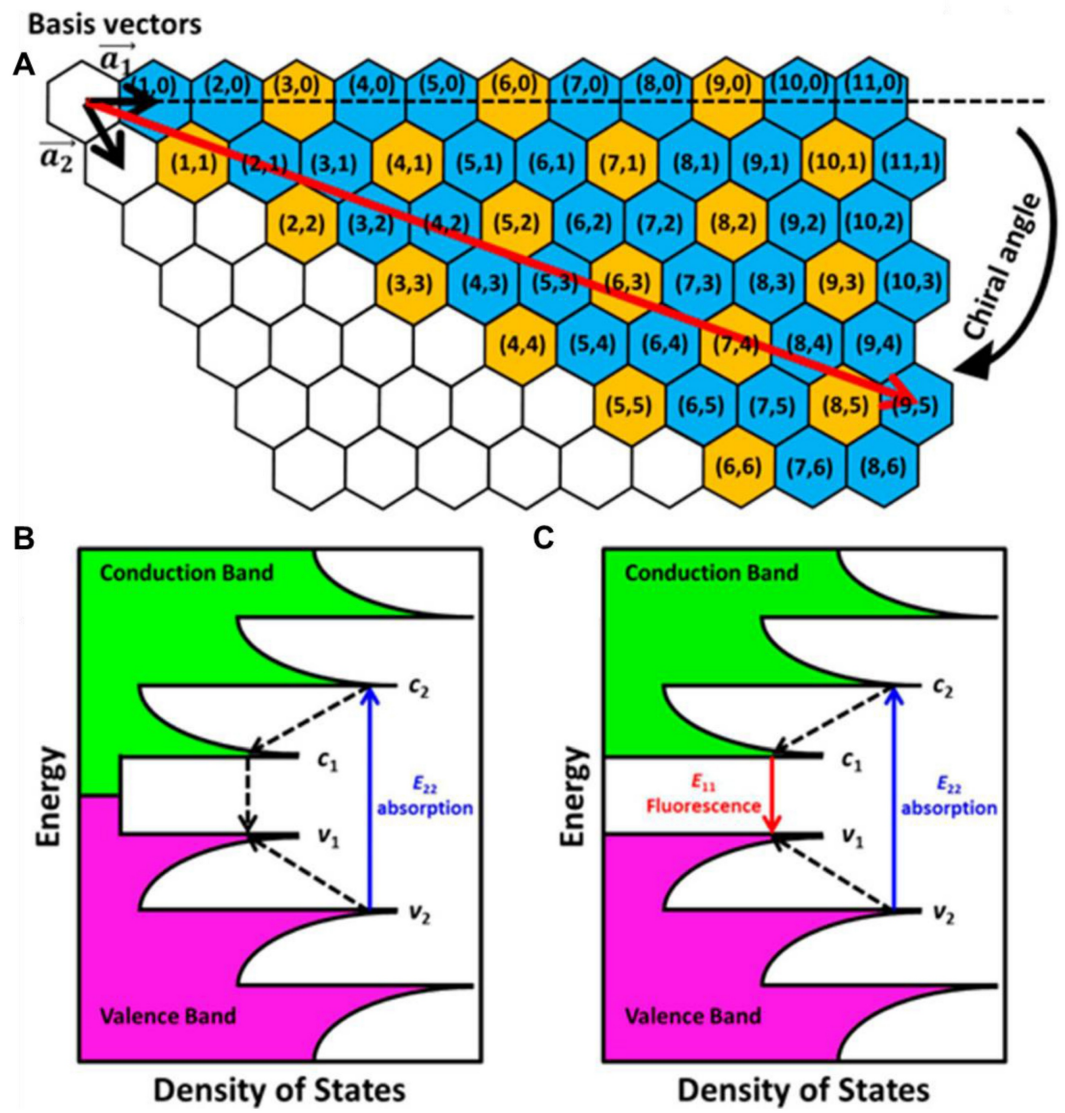
Schematic illustration of physical and electronic band constructions of SWCNTs for NIR-II FL imaging.** (A)** The hexagonal honeycomb-like assembly of graphene demonstrating various roll-up vectors resulted in different indices and chiralities. **(B)** The bandgap structure of metallic carbon nanotubes exhibits no FL emission because of the continuous DOS near Fermi levels. **(C)** The bandgap structure of semiconducting carbon nanotubes exhibits fluorescence emission upon excitation. Adopted with permission from 125, Copyrights 2015 ACS PUBLICATIONS.

SWCNTs are cylindrical nanostructures formed by rolling a single graphite sheet into a tube, with nm scale diameter and lengths extending to the µm range 119. The arc discharge technique is a conventional approach for synthesizing SWCNTs, where an electric arc is created by two carbon electrodes, vaporizing the electrodes and resulting in the formation of SWCNTs 120. Due to their high surface area and exceptional sensitivity to environmental changes, SWCNTs have been an ideal choice for biosensing and molecular recognition applications. Their unique band gap photoluminescence has also made them widely utilized in bioimaging 121. The discovery of SWCNTs with appropriate band gaps for FL in 2002 reported a breakthrough in nanomaterials for *in vitro* and *in vivo* imaging in the medical realm. SWCNTs have extensively been studied as optical biosensors due to their exceptional photostability, FL emission in the NIR region, longer Stokes shifts, and minimal absorption or autofluorescence in tissues and blood 122. Their electronic structure has been characterized by van Hove singularities, or regions of high electronic density of states (DOS) **(Figure 7B and C)**, determined by the size and the chirality angle of the nanotubes **(Figure 7A)**. These chirality angles classify SWCNTs as either semiconducting or metallic, with only semiconducting SWCNTs capable of generating photoluminescence. The emission wavelength of semiconducting SWCNTs can be precisely tuned into the NIR-II window by adjusting their diameters. In 2009, Welsher *et al.* elaborated on the use of modified SWCNTs for NIR-II imaging, heralding a new era for *in vivo* FL imaging in the NIR-II range 17. NIR-II FL from SWCNTs offers significant advantages, such as limited photon absorption, lower tissue scattering, and enhanced tissue penetration, making them highly effective for imaging purposes. Nevertheless, their relatively low photon-conversion efficiency and hydrophobic nature continue to pose challenges for their application in demanding fluorescence imaging scenarios. However, concerns have been raised regarding the potential cytotoxicity of SWCNTs, especially their long-lasting effects on patient health, due to the synthesis process and challenges associated with their elimination from the body. To enable biological applications, careful purification and surface modifications are essential to enhance their water solubility and reduce toxicity 123. Surface modifications can be achieved through both covalent and noncovalent approaches.

NIR-II fluorescent SWCNTs have demonstrated significant potential nanoprobes for numerous *in vitro* and *in vivo* FL imaging applications in the biomedical domain. For instance, to highlight the advantages of the NIR-IIa range, brain imaging *via* intact scalp skin and cranial bones was achieved on mice using three wavelength ranges: NIR-I, NIR-II, and NIR-IIa 39. Mice were *i.v.* injected with SWCNT conjugated with **IRDye-800**, and the brain images captured in the NIR-IIa range revealed the sharpest and most detailed brain vascular structures with superior contrast and resolution. This was attributed to reduced photon scattering at longer wavelengths. Consequently, various major cortical vessels, including the inferior cerebral vein, superior sagittal sinus, transverse sinus, and numerous smaller cerebral branches, were distinguished at imaging depths of 1-2 mm in the NIR-IIa range, all without the need for a craniotomy 124,125.

Although, SWCNTs provided excellent NIR-II FL and deep tissue penetration, their imaging resolution is often compromised by FL variability, aggregation, and photobleaching effects. Moreover, the hydrophobic nature has made their dispersion challenging in biological fluids and requires surface functionalization to improve solubility and reduce toxicity. These limitations have hindered their widespread clinical translation and there is a need to emphasize better synthesis and functionalization strategies.

### 3.5. Carbon dots-based NIR-II fluorescent probes

**Figure 8:**
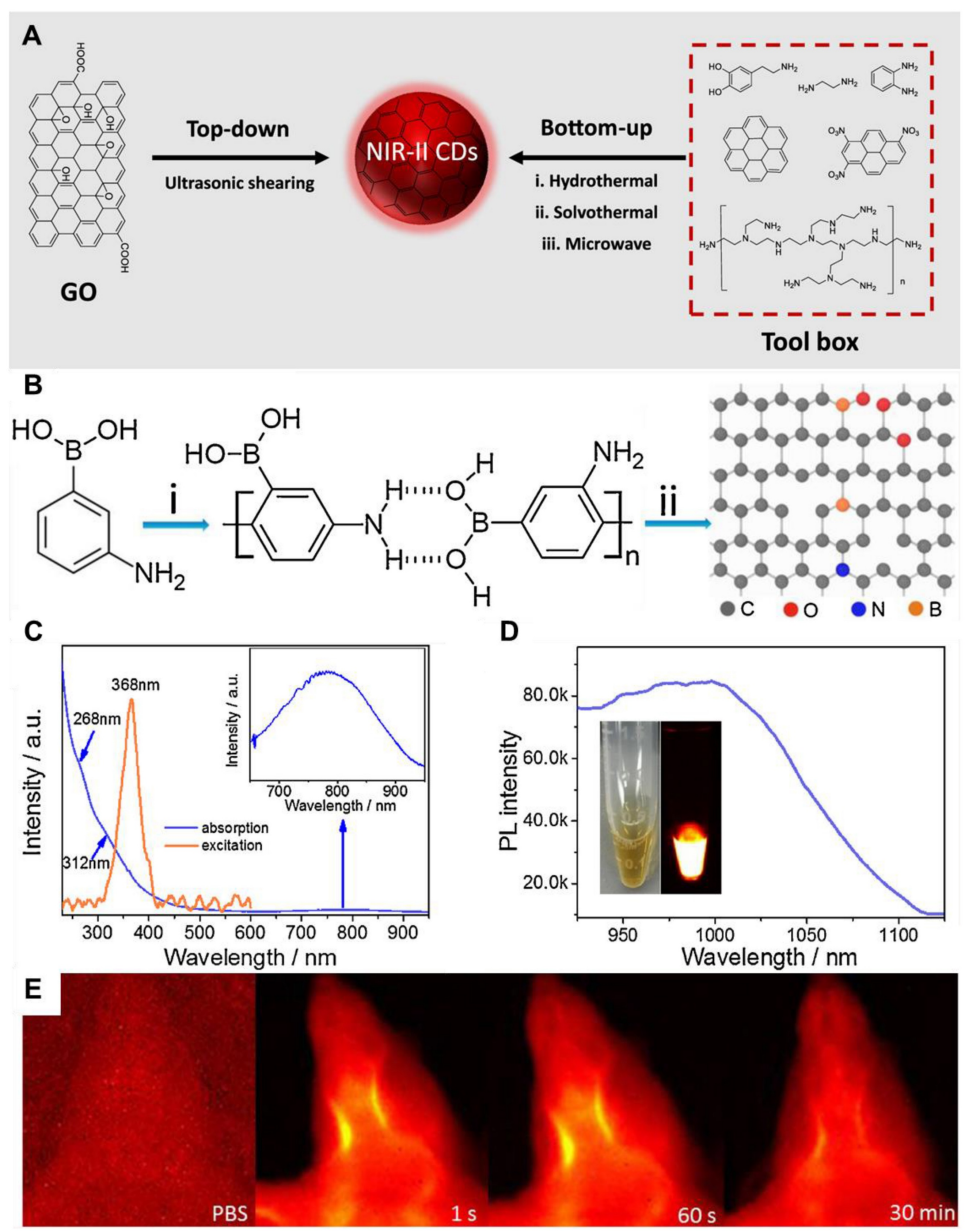
** (A)** Schematic representation of both top-down and bottom-up methodologies for the preparations of NIR-II CDs.** (B)** Schematic representation of the synthetic scheme of the sample: (i) hydrogen bonding mediated synthesis of APBA macromolecules; (ii) macromolecules deposition into the sample.** (C)** UV-vis and NIR absorption spectra of the sample.** (D)** The FL of the sample in the NIR-II range when irradiated with an 808 nm light. The inset demonstrates the optical and NIR-II FL images of the CDs in water.** (E)** NIR-II FL imaging of nude mice demonstrating high magnifications images of the brain blood vessels at different time frames after injecting PBS and sample. Adopted with permission from 126, Copyrights 2024 ACS PUBLICATIONS. Adopted with permission from 132, Copyrights 2019 ELSEVIER.

CDs, known as carbon quantum dots (CQDs), are characterized by their crystalline structures that exhibit the typical lattice of graphitic carbon. Various NIR-II fluorescent CDs have been developed using different precursors and synthetic strategies, which are broadly classified into two main approaches: top-down and bottom-up synthesis **(Figure 8A)** 126. The top-down synthesis involves crushing down bulk carbon materials into smaller fragments, yielding CDs. Common techniques include arc discharge, laser ablation, electrochemical synthesis, chemical etching, and hydrothermal/solvothermal/oxidation cleavage. However, these methods often require harsh conditions, such as strong acids or alkalis, and typically produce CDs with low Φ, broad size distribution, and low purity. In contrast, bottom-up synthesis relies on assembling CDs from molecular precursors using methods such as hydrothermal, solvothermal, and microwave-assisted synthesis, with hydrothermal and solvothermal methods being the most widely utilized. Additionally, a solvent-free synthesis method has also been explored for preparing NIR-II CDs. Bottom-up synthesis provides greater flexibility in selecting precursors, which are often rich in functional groups like -COOH, -NH_2_, and -OH. Due to its simpler process and the broader availability of carbon precursors, bottom-up synthesis has become the preferred approach for NIR-II CD production 127.

Chemical modification is a powerful strategy to enhance the FL emission, colloidal stability, and photothermal performance of NIR-II CDs, making them suitable for cancer diagnosis and therapy. Techniques such as surface modification, element doping, and material hybridization are commonly employed to engineer these nanomaterials 128. Functional groups on the surface and edges of CDs confer excellent solubility in both organic and aqueous solvents. Extensive research has been devoted to developing NIR-II CDs due to their improved tissue penetration, enabling real-time monitoring of biological and physiological processes 129. The ability of NIR-II CDs to absorb and emit light within the same spectral window makes them versatile for noninvasive optical diagnostics, as well as PDT and PTT 130. Their remarkable biocompatibility further ensures safety and reliability in biomedical applications, which is critical for clinical translation. Moreover, the properties of NIR-II CDs can be tailored by doping with elements such as nitrogen, sulfur, boron, or phosphorus or by selecting specific carbon sources 131,132. This enables fine-tuning of their emission properties, achieving redshifted emission spectra, higher Φ, and enhanced photostability. The abundance of chemically modifiable sites also allows for hybridization with other functional materials, enabling multimodal imaging and combination therapies. These features position NIR-II CDs as ideal candidates for cancer theranostics 133.

For example, Wang *et al.* developed a **N- and B-doped graphene quantum dot** (**N-B-GQD**) as an NIR-II fluorescent agent **(Figure 8B)** 132. These **N-B-GQDs**, with ultrasmall size of approximately 5 nm, exhibited excellent serum stability, a FL emission peak at 1000 nm, and higher photostability **(Figure 8C)**. **N-B-GQDs** were administered intravenously, and FL imaging conducted 24 hours later using an 808 nm excitation wavelength confirmed their ability to label tumors. Along with their NIR-II imaging capabilities, the **N-B-GQDs** effectively absorbed NIR light and converted it into heat upon irradiation, enabling a PTT effect. Pure water exhibited a temperature increase of only 3 °C, while water containing **N-B-GQDs** at concentrations of 50, 100, and 200 µg/mL showed temperature increases of 11.1, 19.2, and 26.6 °C, respectively. This efficient photothermal conversion allowed **N-B-GQDs** to effectively destroy tumor cells *in vitro*. *In vivo*, tumor progression was totally halted in mice treated with **N-B-GQDs** and treated using an 808 nm light source. The PTT performance of **N-B-GQDs** was attributed to N and B doping, which induced notable local distortions in electronic energy and created extra energy gaps, as well as created defects causing a redshift in their emission peak. Furthermore, the photophysical characteristics of **N-B-GQDs** could be combined with chemotherapy synergistically, enhancing the overall therapeutic efficacy. The **N-B-GQDs** also demonstrated an excellent safety profile, prolonged blood circulation, and rapid clearance from the body in mice, making them highly suitable for *in vivo* biomedical applications **(Figure 8E)** 132. The above-mentioned works have demonstrated the photostability and biocompatibility of the NIR-II CDs in the biomedical realm. Therefore, more research should be focused on designing NIR-II fluorescent materials possessing higher biocompatibility for advanced medical diagnostics and treatment. However, CDs generally suffer from lower Φ, broad emission spectra, and limited photostability, reducing imaging precision in NIR-II FL-guided therapies. Moreover, their FL intensity may also be highly dependent on synthesis conditions, leading to batch-to-batch variability and inconsistent imaging results. To overcome these drawbacks, optimizing synthesis methods and surface modifications is crucial for achieving stable and high-performance NIR-II FL imaging.

### 3.6. Inorganic/hybrid NIR-II fluorescent probes

**Figure 9:**
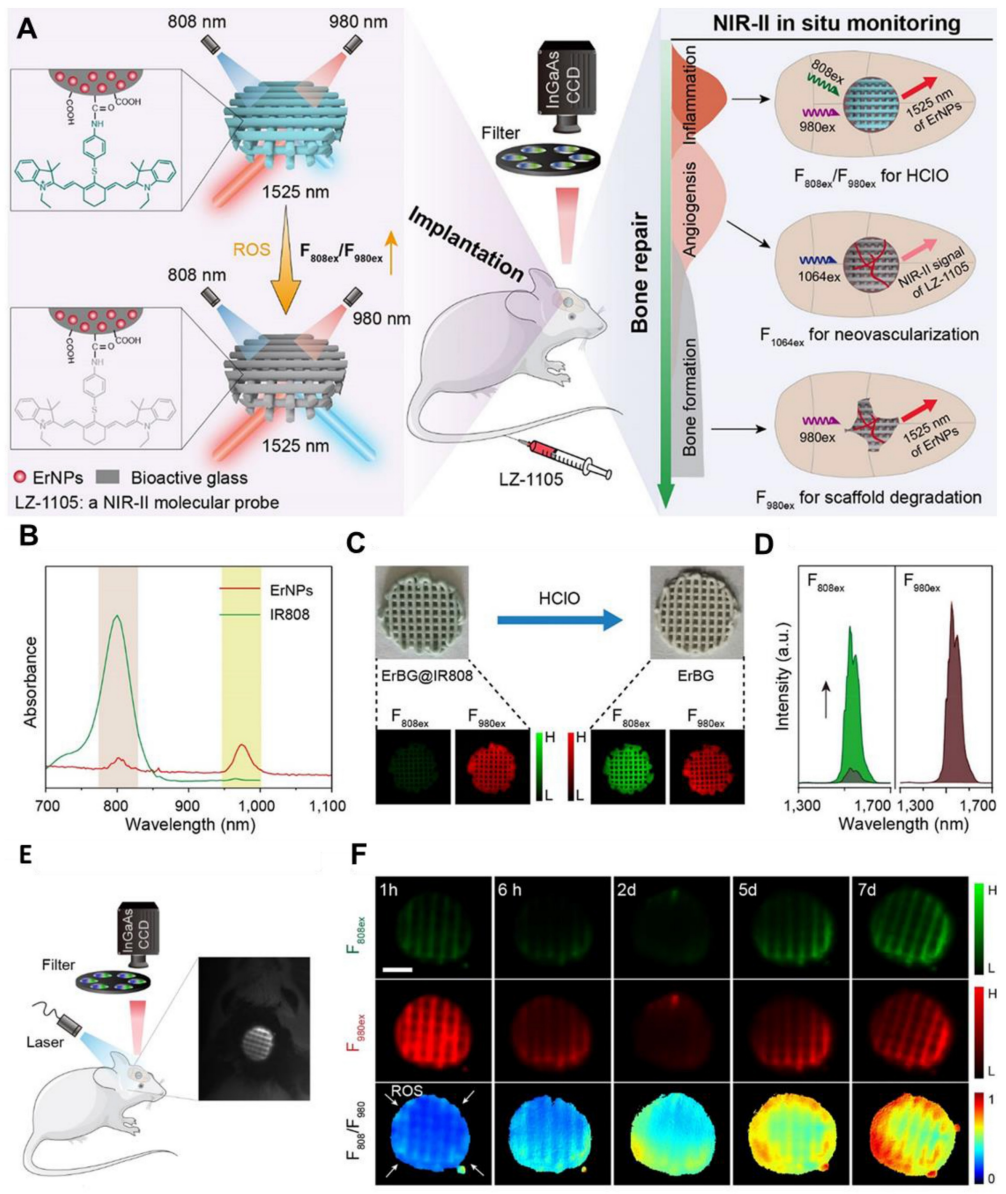
** (A)** Graphical representation of *in situ* bone regeneration visualization *via* NIR-II *in vivo* FL imaging by employing **ErBG@IR808** NIR-II fluorescent scaffolds.** (B)** Demonstration of absorption spectra of different concentrations of IR808 and Er nanoparticles in chloroform.** (C and D)** Demonstration of change NIR-II FL imaging and FL spectra of **ErBG@IR808** NIR-II fluorescent scaffold concerning the presence of HClO upon 808 and 980 nm light irradiation. **(E)** Schematic representation of NIR-II FL mediated monitoring of skull bone inflammation. **(F) ErBG@IR808** NIR-II fluorescent scaffold implant guided NIR-II FL images of mice skull bone defects upon 808 and 980 nm laser irradiation (Scale bar: 2 mm). Adopted with permission from 135, Copyrights 2022 ACS PUBLICATIONS.

The synergistic combination of organic and inorganic components in hybrid nanomaterials is unlocking new possibilities in cancer therapy, particularly for aggressive brain tumors like GBM. By combining the flexibility and biocompatibility of organic materials with the unique properties of inorganic components, these hybrid systems provide a versatile platform for developing targeted therapies with improved efficacy and reduced toxicity 27. One of the most exciting applications of these hybrid materials in GBM therapy is their ability to deliver multiple treatments at once. For example, hybrid nanoparticles can carry chemotherapeutic drugs alongside imaging agents, enabling precise targeting of tumors while also allowing for monitoring of the progress of treatment in real-time. Additionally, these nanoparticles can be designed to release their therapeutic payloads in a controlled manner, ensuring maximum impact on the tumor while minimizing harm to healthy tissues. These materials are also valuable in diagnosing GBM 28. By integrating imaging agents, hybrid nanomaterials can make tumors more visible and easier to detect. For instance, in tiny semiconductor nanocrystals, QDs can be modified to bind specifically to tumor cells, making it possible to identify GBM early and pinpoint its exact location. Hybrid nanomaterials offer significant advantages for PDT and PPT therapies. CDs, a type of nanomaterial with unique optical properties, have been widely studied for these applications. When combined with photosensitizers, they can amplify the effects of light-based therapies by efficiently producing reactive oxygen species that damage tumor cells. Their tunable optical properties further enhance their effectiveness by allowing precise control over how they absorb and emit light 134. Moreover, Pei *et al.* developed an innovative bone regeneration system by designing Er-doped nanoparticles (Er NPs) integrated into a bioactive glass (BG) scaffold, with its surface functionalized using HClO-responsive** IR808** fluorophores **(Figure 9A)** 135. Following implantation into a skull defect, these functional scaffolds significantly enhanced bone regeneration due to their exceptional vascular and osteoblast regeneration properties. Additionally, the Er-based scaffold functioned as an efficient NIR-II FL sensor to track early inflammation by detecting HClO released by inflammatory cells **(Figure 9C and D)**. This detection mechanism relied on an absorption competition-induced emission process between dye and Er-based RENPs **(Figure 9B)**. This ratiometric approach minimized interference from tissue swelling, thereby improving the precision of detection of inflammation** (Figure 9E and F)**. To further investigate bone repair, the NIR-II fluorescent nanoprobe was intravenously injected, enabling visualization of new blood vessel formation and spatial localization in the bone defect area through accumulation of both LZ-1105 and Er-based RENPs. The long-term *in vivo* biodegradation of the Er-based scaffold was monitored using the 1525 nm FL of Er-based RENPs under 980 nm illumination. Both *in vitro* and *in vivo* tests confirmed the excellent biocompatibility and osteogenic potential of the Er-based scaffolds. These findings highlighted the promising potential of **ErBG@IR808** scaffolds for real-time monitoring and enhanced progression of skull bone repair. The mentioned nanohybrid strategy can also be applied for diagnosing the tumors within the body like GBM because of biocompatibility and deep penetration of the NIR-II light. The rear earth metals can also be combined with organic mioties to design efficient nanohybrids for efficient imaging applications 136.

Furthermore, Zhao *et al.* fabricated Au nanohybrids with bright NIR-II FL and tunable structures, enabling real-time visualization of vascular problems 137. The amphiphilic block copolymer-assisted self-assembly approach applied was straightforward, reproducible, and effective in preventing FL quenching during the assembly process. Pluronic F127 served as the organic template, directing the growth and assembly of ultrasmall Au nanoparticles in the core while introducing PEG chains on their surface. By altering hydrophobic multidentate thiol ligands, two distinct Au nanohybrid structures were synthesized: necklace-like Au nanohybrids with dense brush PEG coatings and spherical Au nanohybrids with brush PEG surfaces. Both types exhibited excellent photostability, deep tissue penetration, and good biocompatibility. However, the necklace-like Au nanohybrids demonstrated superior performance, including higher Φ in the NIR-II region, enhanced protein adsorption resistance, and prolonged blood circulation, because of their dense PEG brush coating. The mentioned approach successfully combined bright NIR-II FL with extended circulation properties. As a proof of concept, the necklace-like Au nanohybrids were employed for real-time imaging of thrombolysis, marking a novel application of fluorescent Au nanoparticles. Their exceptional performance highlights the potential for broader applications, such as GBM diagnosis, in the future 137. Additionally, Liu *et al.* developed an innovative nano-platform for NIR-II FL imaging-guided, activatable SO_2_ release for treating orthotopic GBM. The designed platform consisted of core-shell **NaYF_4_:Yb/Tm@NaYF_4_:Nd** nanoparticles exhibiting both upconversion and downshifting FL under NIR irradiation conjugated with the prodrug 5-Amino-1,3-dihydrobenzo[c]thiophene 2,2-dioxide (ATD), which can release SO_2_ when exposed to ultraviolet light emitted by the sample, and the organic dye **IR-808** significantly enhancing the upconversion and downshifting FL of sample nanoparticles due to its superior absorption cross-section and extinction coefficient compared to rare-earth ions. The sensitization of the sample with **IR-808** led to a 28-fold increase in upconversion FL at 348 nm and a fivefold enhancement of NIR-II FL at 1340 nm. The improved NIR-II FL enabled precise imaging and diagnosis of orthotopic GBM, while the intensified UV emission effectively triggered controlled SO_2_ release. This, in turn, enhanced pro-death autophagy, ultimately inducing GBM cell apoptosis. By integrating imaging and on-demand therapy, the designed nano-platforms offered a promising approach to minimizing drug side effects while maximizing therapeutic efficacy.

## 4. Therapeutic paradigms with NIR-II emissive inorganic/hybrid nanomaterials

NIR-II fluorescent inorganic and hybrid nanomaterials have gained significant attention in biomedical imaging and therapy due to their exceptional optical properties in NIR-II windows. This window offers deeper tissue penetration, reduced autofluorescence, and enhanced resolution for *in vivo* imaging applications. Hybridization with biocompatible coatings further improves their targeting, biodistribution, and therapeutic potential for advanced diagnostic and therapeutic modalities, particularly for GBM treatment. In this section, inorganic and hybrid fluorescent nanomaterials-based imaging-guided modern therapies for GBM have been discussed in detail.

### 4.1. NIR-II FL imaging-guided surgery of GBM

NIR-II FL imaging offers high spatiotemporal resolution and real-time tumor visualization, aiding oncological surgeons in maximizing tumor removal, minimizing damage to healthy tissues, and reducing surgical time.^138^ NIR-II FL imaging-guided surgery is the potential treatment modality for the efficient management of GBM because of the clear differentiation between tumor margins and healthy brain tissues minimizing the invasiveness of the surgical process 139. To model the common malignant glioma, the transgenic ND2:SmoA1 mouse model is frequently studied. For example, Liu *et al.* analyzed the cerebral vasculature characteristics of healthy and ND2:SmoA1 mice using NIR-II FL imaging and quantified tumor malignancy based on vascular morphology through a segmentation and quantification algorithm 140. Their findings revealed that medulloblastoma mice exhibited significantly higher total vessel length, more vessel branches, and greater vessel diameter entropy compared to the uniform and hierarchically structured normal vasculature. Moreover, Ren *et al.* demonstrated the potential of strong NIR-IIb FL by using Er-based RENPs for imaging-guided surgery of orthotopic gliomas 103. The authors developed an energy-cascaded Er^3+^-Ce^3+^-A^3+^ (A = Yb, Ho, Tm) system and synthesized Er-based RENPs, optimizing the effects of the NaAF_4_ interlayer and Ce^3+^ dopants. The optimal Er-based RENPs were modified with a Dye-brush polymer to enhance the ^4^I_13/2_ → ^4^I_15/2_ transition, achieving a remarkable 675-fold increase in 1525 nm FL in aqueous solution under 808 nm excitation compared to **NaErF_4_** RENPs, due to highly efficient energy-cascaded downconversion. To target gliomas, these bright nanoparticles were further functionalized with the angiopep-2 peptide and delivered to the tumor using focused ultrasound sonication (FUS) to temporarily open the BBB. The targeted NIR-IIb FL imaging achieved the highest SBR reported for small orthotopic gliomas through intact skull and scalp, further enhanced to ~150 after cardiac perfusion and craniotomy ensuring accurate tumor deletion. Notably, the glioma size measured from the FL profile closely matched that obtained from T_2_-weighted MRI images. The mentioned work provided valuable insights for engineering NIR-IIb FL in RENPs and highlighted their immense potential for NIR-IIb FL imaging-guided tumor surgery 140.

**Figure 10:**
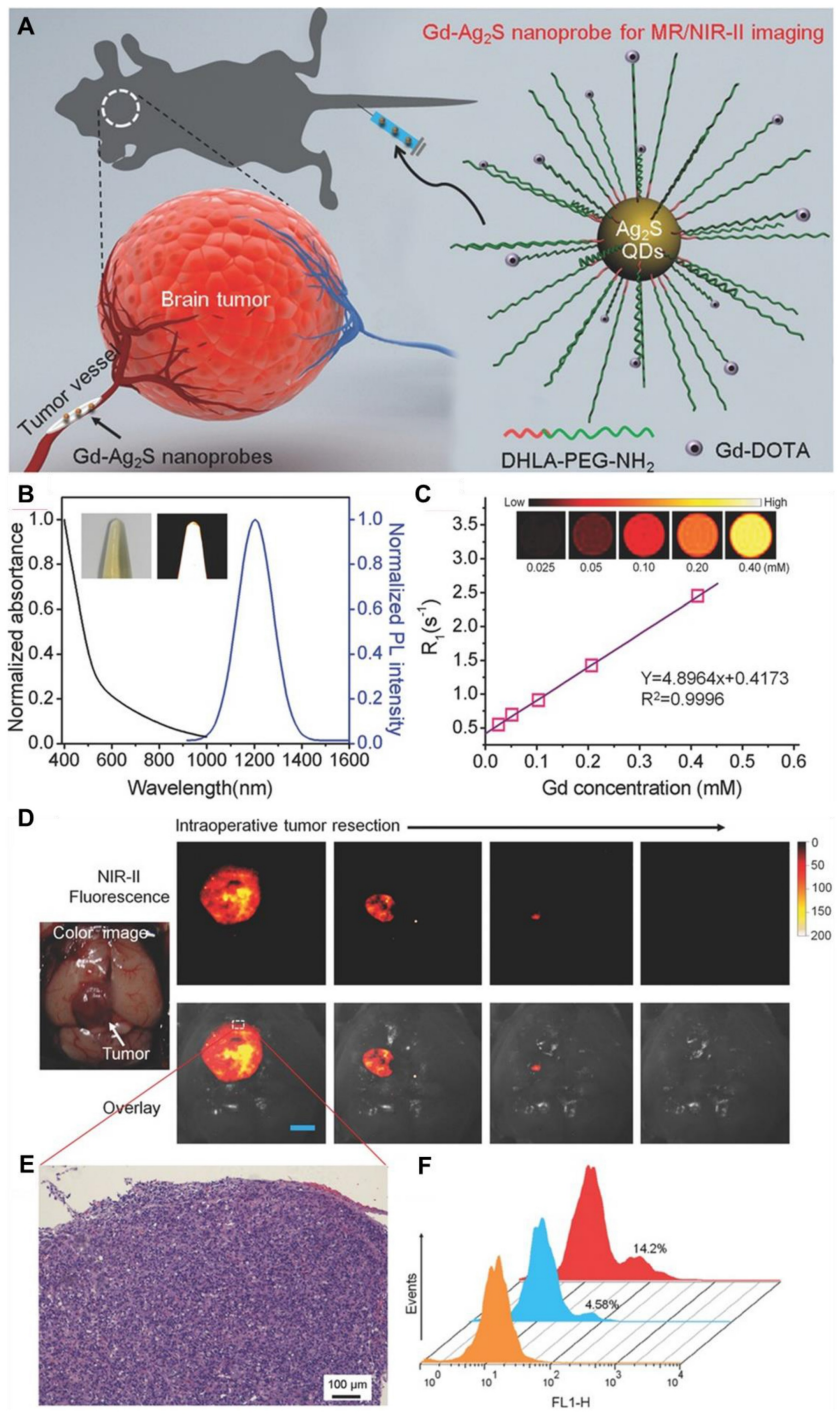
** (A)** Schematic illustration of the targeted brain tumor visualization by employing **Gd-Ag_2_S** NIR-II fluorescent nanoprobe. **(B)** Absorption and emission spectra of **Gd-Ag_2_S** NIR-II fluorescent nanoprobe (Insets represent the optical and FL images upon visible and 808 nm laser irradiation, respectively). **(C)** T_1_ relaxivity graph of the nanoprobe representing the MRI efficiency of the probe (the inset represents the T_1_ mapping of an MR imaging of the **Gd-Ag_2_S** NIR-II fluorescent nanoprobe at different molarity). **(D)** NIR-II FL imaging-guided surgery of U87MG tumor-bearing mice (Scale bar = 2.5 mm). **(E)** H&E-stained of the surgically removed tumor tissue representing the tumor margins. **(F)** Flow cytometric graphs of the residual tumor tissues representing the tumor removal efficiency of the surgical process. Adopted with permission from 142, Copyrights 2015 WILEY.

The amalgamation of NIR-II FL imaging for real-time intraoperative tumor visualization with clinical techniques like MRI and PET for precise preoperative diagnosis has gained significant consideration. Recently, following FUS and the orthotopic glioma resection guided by T_2_-weighted MRI and NIR-IIb FL imaging with RENPs, no significant false FL signals were detected in the tissue surrounding the resected tumors 141. Furthermore, Li *et al.* developed the first dual modal "detection and operation" fluorescent nanoprobe, **Gd-Ag_2_S**, to delineate U87MG in a mice model at various time frames **(Figure 10A)** 142. Three hours after nanoprobe administration, brain tumors were accurately localized using prepositioned T_1_-weighted MRI. The high SBR imaging of **Ag_2_S** QDs at 1200 nm enabled clear visualization of tumor margins **(Figure 10D)**. Histological analysis confirmed the complete resection of the brain tumor **(Figure 10E)**, and flow cytometry showed that tumor excision guided by **Gd-Ag_2_S** was significantly more effective than excision by visual observation alone **(Figure 10F)** 142.

Alongside MRI, PET is also utilized as a preoperative imaging technique to guide the removal of cancerous cells. For example, Shi *et al.* introduced PET/NIR-II dual-modality imaging nanoprobes, which specifically target the folate receptor overexpressed in GBM, offering significant potential for image-guided GBM surgery 143. However, the complete removal of all the tumor cells during the surgical process is nearly impossible. There is always a risk of tumor recurrences further complicating the repeated surgery. Therefore, there should be a post-surgery treatment modality to suppress the tumor recurrence for better patient healthcare and enhanced survival rate.

### 4.2. NIR-II FL imaging-guided chemotherapy of GBM

**Figure 11:**
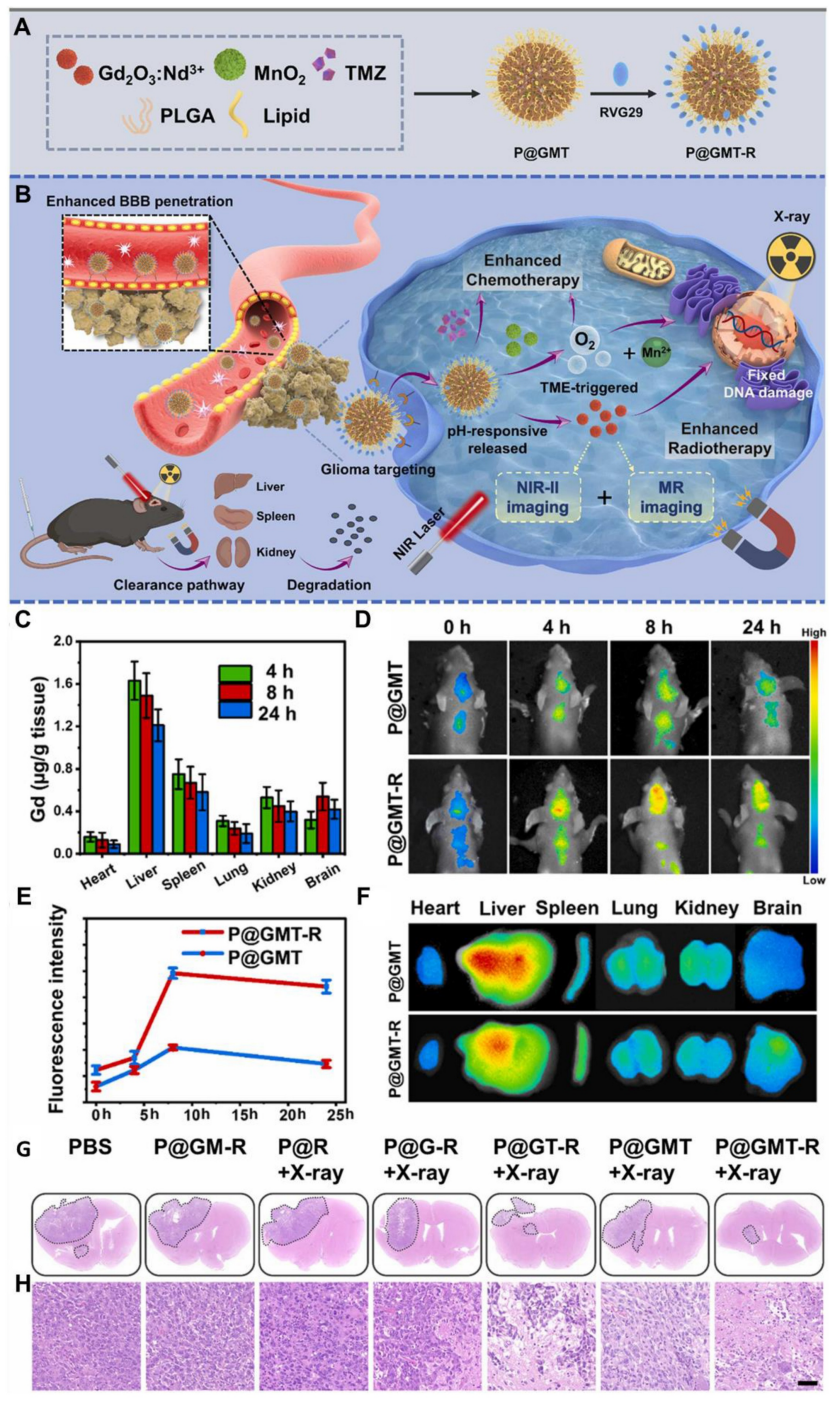
** (A)** A pictorial illustration of the synthetic methodology of the sample nanocapsules.** (B)** Graphical illustration of therapeutic and diagnostic mechanisms of the sample nanocapsules.** (C)** The distribution of Gd content within the body after the *i.v.* injection of different samples at different time frames.** (D)** Demonstration of *in vivo* NIR-II FL imaging of the intracranial glioma within the mice treated with prepared samples upon 808 nm laser illumination at designed time frames.** (E)** The measured FL intensity of different samples after injection at various time frames.** (F)** Representation of *ex vivo* NIR-II FL images of the major body organs and tumors at 8 hours of injection with different samples.** (G)** Demonstration of histological comparison of GBM-bearing mice when subjected to different treatments indicated. **(H)** The respective H&E-staining of the GBM slices of different groups after different treatments. Adopted with permission from 147, Copyrights 2022 ELSEVIER.

Chemotherapy, employing numerous chemotherapeutic medicines, has been essentially significant for clinical tumor therapeutic modalities over the past few decades. Chemotherapy works by affecting the cell cycle, preventing cancer cells from replicating and spreading. Different chemotherapeutic drugs function through distinct mechanisms, such as interfering with DNA synthesis, damaging cellular structures, or inhibiting protein production 30. While chemotherapy can be effective in treating glioma cells, it often causes significant side effects, including hair loss, nausea, and vomiting, due to its impact on healthy cells. One promising technique to enhance the efficacy of chemotherapy for gliomas is the use of NIR-II guided therapy. NIR-II technology allows for the precise delivery of chemotherapeutic drugs to the tumor site 144. This technique can be combined with various nanomaterials, such as QDs, RENPs, MNCs, SWCNTs, and CDs which absorb NIR-II light and convert it into heat or other forms of energy. This energy can then be employed to directly eradicate cancer cells or to trigger the release of chemotherapy drugs at the tumor site. However, the effectiveness of current chemotherapy is often limited by side effects resulting from nonspecific drug distribution and the potential for drug overdose. TMZ is a first-line chemotherapy drug for GBM multiforme, but its clinical efficacy remains suboptimal due to issues such as poor targeting, drug resistance, and systemic toxicity. To address these challenges, various smart drug delivery systems (DDSs) have been developed, including polymeric nanoparticles, silica nanoparticles, and Au nanoparticles. These systems are designed to respond to the unique TME, which is characterized by factors such as low pH, high glutathione (GSH) levels, and enzymes 145. Additionally, external stimuli like light or ultrasound can trigger the release of drugs, enabling targeted delivery and reducing systemic toxicity. For effective brain tumor treatment, these DDSs often need surface functionalization and optimal particle sizes to ensure efficient targeting and drug delivery. QDs have emerged as promising NIR-II FL imaging nanoprobes and drug carriers because of their excellent biocompatibility, reduced cytotoxicity, superior FL properties, and enhanced drug-loading capabilities. However, many formerly reported CDs suffer from nonspecific contact with both healthy and cancer cells. A key solution is the overexpression of large neutral amino acid transporter 1 (LAT1) in various tumors and BBB, which facilitates the transport of large neutral amino acids. This characteristic can be leveraged for the targeting of brain tumors *via* theranostic nanomaterials 146. For instance, Yin *et al.* developed innovative theranostic nanocapsules used as a carrier to encapsulate ultrasmall rare earth metal-based **Gd_2_O_3_:Nd^3+^** nanodots, MnO_2_ nanoparticles, and the commonly used anti-GBM drug TMZ 147. To enhance targeting capability and enable the crossing of the BBB, the surface of the nanocapsules was modified with the glycoprotein of rabies virus **(Figure 11A)**. The paramagnetic properties and efficient NIR-II FL of **Gd_2_O_3_:Nd^3+^** nanodots provided the nanocapsules with excellent performance as nanoprobes for MRI and NIR-II FL imaging. The carriers enhanced the biological availability of TMZ within the brain, reduced general side effects by prolonging the blood circulation time, and enabled controlled secretion at the tumor region, activated by the tumor TME, thereby improving the efficacy of targeted chemotherapy **(Figure 11C and D)**. Additionally, MnO_2_ nanoparticles generated oxygen by decomposing H₂O₂ at the tumor site, alleviating hypoxia and enhancing the effects of targeted chemo/radiotherapy for GBM **(Figure 11B)**. The release of Mn^2+^ also facilitated self-enhanced MRI. Notably, the TME-responsive biodegradability of MnO_2_ nanoparticles, the biocompatibility of PLGA, and the renal clearance ability of **Gd_2_O_3_:Nd^3+^** nanodots ensured the nanocapsules were highly biocompatible, supporting their clinical potential. The mentioned discussion collectively demonstrated that the nanocapsules were a promising platform for the effective diagnosis and treatment of GBM 147.

Moreover, Hu *et al.* engineered innovative core-satellite down/upconversion nanoparticles (D/UCNPs) featuring significant one-excitation triple-emission FL for synergistic GSH-activated ratiometric NIR-II FL imaging and chemo/PDT 148. The nanoprobes, termed **D/UCNP@cgAuNCs**, were created by functionally assembling cyclodextrin (CD) and GSH stabilized Au nanoclusters onto the surface of D/UCNPs using coupling chemistry. When excited by a laser 808 nm, the nanoprobes exhibited three distinct FL bands: two in the NIR-II region at 1060 and 1550 nm and one in the visible region at 660 nm. The dual-ligand strategy significantly enhanced the FL response of Au nanoclusters to endogenous GSH, enabling NIR-II FL signals for tumor diagnosis. Furthermore, the therapeutic ability of the nanoprobes was strategically engineered by leveraging the visible FL to stimulate the methylene blue photosensitizer and utilizing the CD cavity to load doxorubicin (DOX). This spatially divided functionalization confirmed precise NIR light-activated PDT combined with pH-sensitive chemotherapy. As a result, these nanoprobes achieved TME-enhanced NIR-II FL imaging alongside chemo-/PDT, providing a powerful approach for more precise tumor diagnostics and therapeutics 148.

Though, chemotherapy is a well-known therapeutic modality applied for the treatment of cancer after surgery to control tumor metastasis and enhance the patient survival rate. However, the toxicity, side effects, and drug resistance by the tumor cells are the major challenges to tackle in the case of chemotherapy-based cancer treatment. Therefore, the use of modern non-invasive treatment modalities could be an efficient step ahead in the field of cancer treatment. The use of modern non-invasive treatment modalities has the potential to revolutionize the medical field in the near future.

### 4.3. NIR-II FL imaging-guided photothermal therapy of GBM

**Figure 12:**
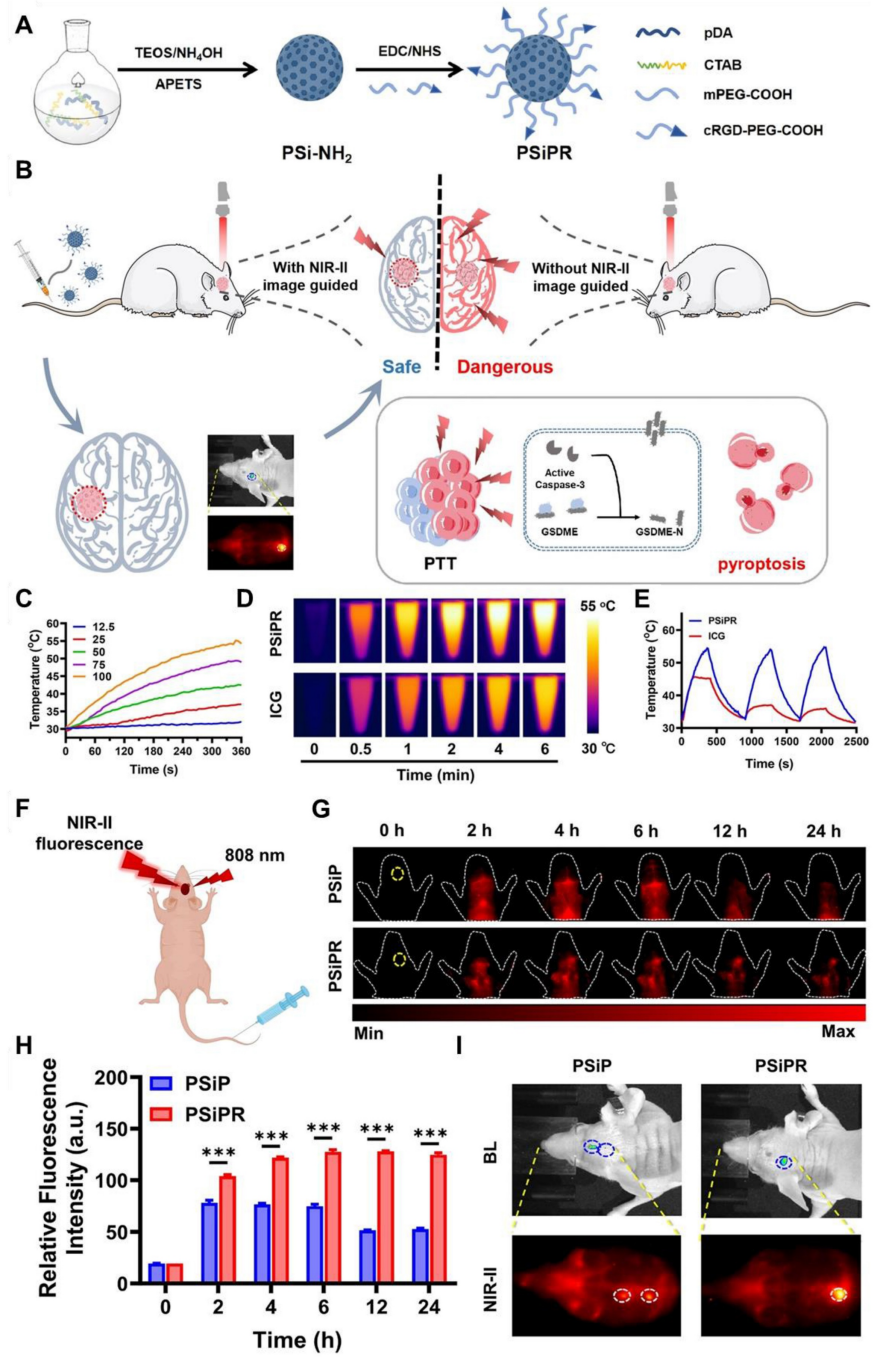
** (A)** Schematic demonstration of the synthetic scheme of the sample.** (B)** Schematic representation NIR-II FL imaging-guided PTT precisely inducing the pyroptosis activation. **(C)** Representation of photothermal curves of the sample at different concentrations upon 808 nm light illumination.** (D)** Comparison of the photothermal ability of the sample with ICG upon 808 nm light illumination.** (E)** Comparison of photothermal cycling curves of the sample with ICG for demonstration of the photothermal ability of the sample. **(F)** Schematic demonstration of NIR-II FL imaging of the mice bearing orthotopic U87MG. **(G)** NIR-II FL imaging of orthotopic brain tumor-bearing mice at different time frames by using functionalized and unfunctionalized samples. **(H)** Demonstration of relative FL intensity of the orthotopic brain tumor areas at suggested time frames for validating the targeting. **(I)** NIR-II FL imaging of the mice skull with comparison to the bioluminescence imaging. Adopted with permission from 150, Copyrights 2025 WILEY.

Photothermal therapy (PTT), an advanced therapeutic approach for treating GBM, is leveraging the unique properties of nanomaterials to transduce absorbed light into precisely localized thermal energy, thereby inducing targeted destruction of tumor cells with high efficacy. Inorganic QDs have emerged as promising photothermal agents (PTAs) due to their exceptional optical properties, including tunable size-dependent absorption and emission in the NIR-II region 149. The NIR-II window is particularly advantageous for GBM therapy, as it offers deeper tissue penetration and reduces light scattering and absorption by biological tissues. Advancements in QD nanotechnology have enhanced their potential in PTT applications. For example, **N and B-doped GQDs** exhibit high photothermal conversion efficiencies, reaching up to 62.53%, owing to the creation of additional energy gaps and the redshifted emission in the NIR-II region. These dopants also improve light absorption and stability, making them ideal for diagnosing and treating deep-seated tumors like GBM 132. Moreover, functionalization of QDs with targeting ligands allows for selective accumulation in GBM cells, minimizing damage to healthy tissues and better healthcare. For example, Chen *et al.* developed a PTT agent based on semiconducting polymers designed to enable precise pyroptosis activation under NIR-II FL imaging guidance in a glioma-bearing mouse model **(Figure 12A and B)** 150. Semiconducting polymers were utilized as the optical material, while mesoporous silica was employed as the shell to create nanoparticles with NIR-II FL properties and excellent PTT performance **(Figure 12C, D, and E)**. To extend blood circulation time, the nanoparticles were modified with PEG on their outer surface, resulting in PEG-stabilized nanoparticles referred to as **PSiP**. To achieve glioma-targeting capability, the cRGD targeting peptide was conjugated to **PSiP**, forming the glioma-targeting nanoparticle platform, **PSiPR**. Using **PSiPR**, the researchers successfully induced pyroptosis in U87MG cells *in vitro* and performed NIR-II FL imaging of both subcutaneous and orthotopic gliomas **(Figure 12G, H, and I)**. Moreover, they precisely activated pyroptosis *in vivo* under image guidance, demonstrating the effectiveness of the sample for glioma therapy 150.

Moreover, RENPs have demonstrated remarkable potential for imaging-guided tumor therapy, particularly in PTT, which leverages the conversion of photonic energy into localized thermal energy to eradicate tumors while sparing surrounding healthy tissues. Tunable NIR-II FL properties and the ability to combine therapy with real-time imaging make RENPs ideal candidates for PTT. For example, Jiang *et al.* introduced a versatile *in situ* growth strategy to develop a multifunctional **NaLnF_4_@Cu_2-x_S** core-satellite nanostructure as a theranostic nanoplatform 151. *In vitro* and *in vivo* NIR-II FL imaging demonstrated that these **NaLnF_4_@Cu_2-x_S** probes offered exceptional detection sensitivity and were effective for diagnosing small tumors. Additionally, the researchers successfully achieved through-skull, non-invasive brain vessel imaging without the need for craniotomy. The **NaLnF_4_@Cu_2-x_S** probes also exhibited a strong photothermal effect with minimal side effects. These characteristics make the **NaLnF_4_@Cu_2-x_S** nanocomposite a promising platform for highly sensitive bioimaging and efficient PTT. Another innovation was flower-like **NiS_2_-coated NaLuF_4_:Nd** nanoparticles, combining NIR-II FL imaging capabilities with excellent PTT performance. The flower-like structure enhanced photon scattering, boosting the photothermal conversion efficiency to 39.38% under 808 nm irradiation, with tumor surface temperatures reaching 55°C. Functionalization with epigallocatechin gallate, an HSP90 inhibitor, further improved the therapeutic outcomes by suppressing heat shock protein-mediated tumor tolerance 151. Furthermore, inorganic materials also offer promising avenues for PTT like **CuS** QDs grown on **NaYF_4_** nanorods formed satellite-like structures with PTT efficiencies of 32% while maintaining NIR-II FL imaging capability. These hybrid probes ablated small tumors (~5 mm) *in vivo* with high SBR 152. Similarly, Nd-doped core-shell nanoparticles coated with polydopamine (PDA) achieved multifunctionality. PDA provided superior biocompatibility, colloidal stability, and photothermal efficiency while enabling simultaneous NIR-II FL imaging and tumor therapy 153. These advancements underscore the potential of RENPs as multifunctional agents for PTT, offering real-time imaging guidance and enhanced therapeutic efficacy with minimal side effects 202. Further optimization in biocompatibility and clinical translation is crucial to realize their full potential in precision medicine.

### 4.4. NIR-II FL imaging-guided photodynamic therapy of GBM

**Figure 13:**
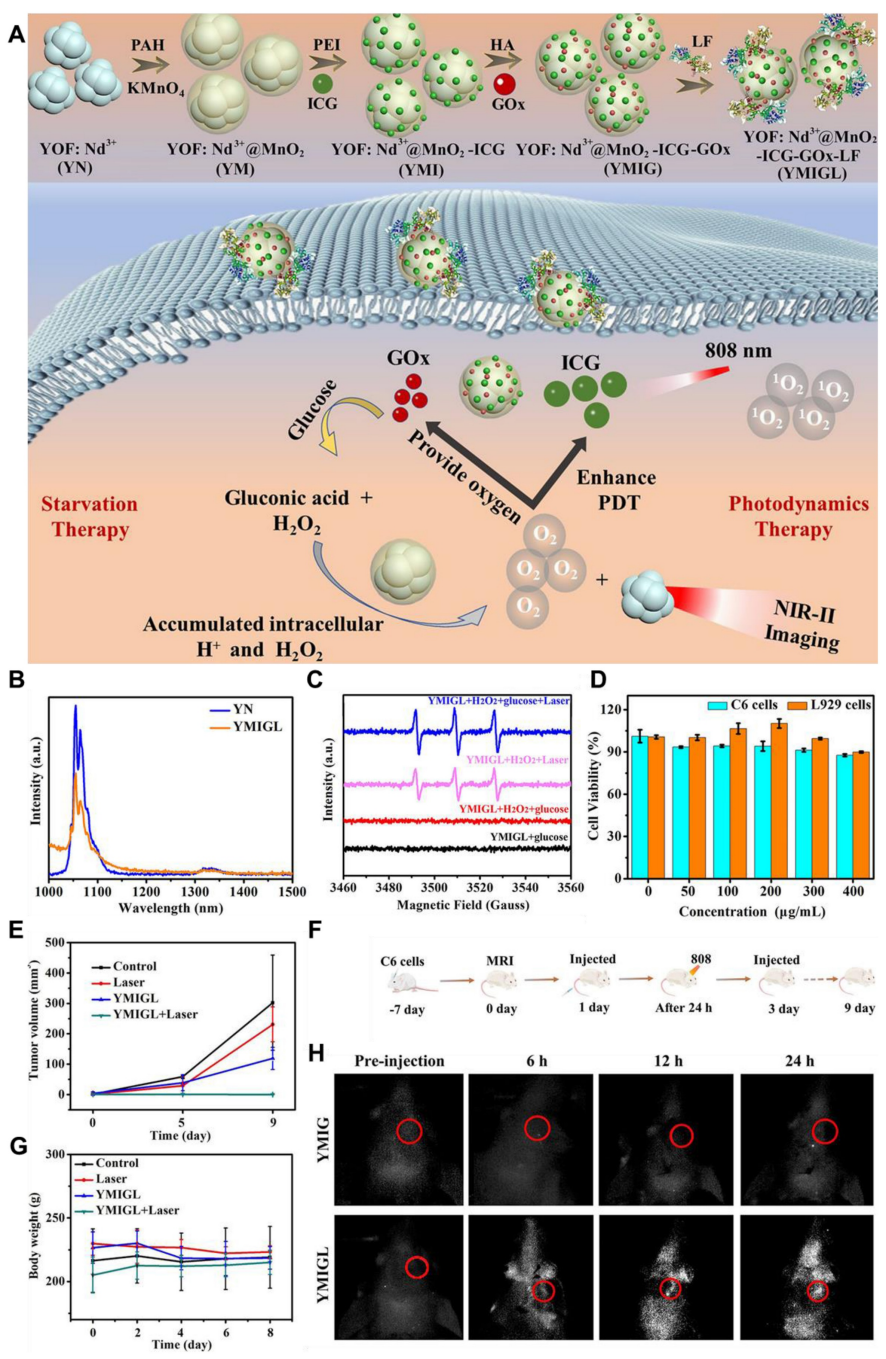
** (A)** Graphical illustration of the synthetic process, diagnosing ability, and treatment mechanism of YMIGL. **(B)** Representation of downconversion FL spectra of different samples upon 808 nm light irradiation with 0.4 W/cm^2^ in simulated TME. **(C)** Representation of ESR spectra of different samples to confirm the ROS production upon 808 nm light irradiation.** (D)** Cytotoxicity of the sample incubated with L929, C6 cell lines upon 808 nm laser excitation with 1 W/cm^2^ power for 7 minutes time duration.** (E)** The demonstration of variation in tumor volume after sample administration and therapy.** (F)** Schematic illustration of treatment and monitoring cycle plan. **(G)** Demonstration of change in body weight after sample administration and therapy. **(H)** NIR-II FL imaging of orthotopic glioma-bearing mice when injected with the sample 24 hours after injection. Adopted with permission from 155, Copyrights 2022 ACS PUBLICATIONS.

Photodynamic therapy (PDT), a light-driven cancer treatment, relies on the activation of photosensitizers (PS) to generate ROS-inducing tumor cell death. The NIR-II fluorescent probes have revolutionized PDT by serving as energy donors for PS, significantly enhancing the efficiency of ROS production 154. The photodynamic process involves the excitation of PS-loaded probes by NIR-II light, promoting electrons to a higher energy state. Through intersystem crossing, these excited electrons transition to a triplet state, where they interact with oxygen to produce ROS which can be used for the treatment of GBM. This can occur through two pathways: Type I reactions, involving free radical formation, and type II reactions, which directly generate singlet oxygen (^1^O_2_) 32. For example, Lv *et al.* developed novel theranostic agents, LF-modified YMIGL, which incorporate a **YOF:Nd^3+^** core, a MnO_2_ shell, and is loaded with a photosensitizer and GOx *via* electrostatic interaction **(Figure 13A)** 155. This agent is designed for NIR-II FL-guided synergistic starvation therapy and PDT for treating orthotopic glioma. The LF modification enables YMIGL to cross the BBB and specifically target glioma cell receptors. Once inside the glioma cells, hyaluronic acid-coated GOx is released, facilitating glucose consumption for ST and generating H_2_O_2_ and gluconic acid for subsequent cascade reactions. The accumulated H_2_O_2_ reacts with the MnO_2_ shell in the acidic TME, producing O_2_ to enhance PDT efficacy **(Figure 13C)**. Additionally, the **YOF:Nd^3+^** core emits NIR-II FL at 1064 nm, allowing precise orthotopic glioma diagnosis **(Figure 13B and H)**. The combination of ST and PDT demonstrates significant inhibition of glioma growth both *in vitro* and *in vivo*, positioning YMIGL as a promising nanoagents for glioma therapy **(Figure 13E and G)** 155.

Among the NIR-II fluorescent probes, RENPs also have emerged as promising agents for having the ability to emit tunable FL, including visible light, and NIR excitation, which enhances the penetration depth of PDT and broadens its therapeutic applications. For instance, for multimodal imaging-guided CDT/PDT combined therapy, Lin *et al.* presented Mn and Cu doped silicate nanosphere-covered lanthanide-based nanoparticles also known as **PEG/LDNPs@CMSNs** 156. CMSNs acted as PS, triggered by upconversion emission, to produce ^1^O_2_ for PDT when illuminated by a 980 nm laser. In the meantime, the high levels of GSH in the TME caused CMSNs to degrade by releasing Cu^+^ and Mn^2+^ through a redox process, which enhanced the CDT impact. Importantly, Ce doping improved the NIR-II emission of Er^3+^, and the presence of Mn^2+^ gave the system MRI capabilities. Moreover, Wang *et al.* developed a novel strategy for tumor treatment combining NIR-II imaging with NIR-I-activated PDT 157. A nanoprobe integrating red blood cells and RENPs for FL-guided PDT upon 980 nm laser illumination was developed. The designed nanocomposite, **UCNPs@RB@RGD@avidin**, incorporated rose Bengal (RB) PS and RGD peptide for tumor targeting. RBCs enhanced PDT efficiency by delivering oxygen to the tumor site. Upon 808 nm laser irradiation, the RBCs released ICG and oxygen, enabling both NIR-II imaging for tumor visualization and PDT-induced tumor destruction. This combined approach offered a promising strategy for cancer treatment 157.

The NIR-II fluorescent probes have shown significant promise in enhancing PDT. Due to co-doping with other metal ions or constructing core-shell architectures, the probes can achieve higher brightness and efficient energy transfer to photosensitizers, enabling ROS generation deep within tissues. The ability of probes to penetrate deep-seated tumors, aided by their NIR-II emissions, overcomes the challenges posed by visible or UV light in conventional PDT. Additionally, the probes serve as multifunctional platforms by being conjugated with targeting agents, photosensitizers, or therapeutic molecules, allowing for tumor-specific therapies with real-time imaging guidance. Despite their potential, challenges such as low Φ, cytotoxicity concerns, and limited clinical models must be addressed to fully exploit NIR-II probes in PDT for clinical applications.

### 4.5. NIR-II FL imaging-guided chemodynamic therapy of GBM

Chemodynamic therapy (CDT) is a developing cancer treatment modality that utilizes CDT agents to convert H_2_O_2_ into hydroxyl radicals (•OH), which are highly reactive and cytotoxic ROS generated through Fenton/Fenton-like reactions. These radicals induce apoptosis and necrosis in cancer cells.^33^ As a non-invasive and novel therapeutic approach, CDT has gained significant attention in the treatment of GBM. It involves introducing nanoagents, often based on transition metals, into tumor cells to release metal ions that catalyze the Fenton and Fenton-like reactions. The breakdown of H_2_O_2_ by these metal ions generates •OH radicals, which accumulate and trigger cell death, forming the basis of the therapeutic mechanism of CDT 158. Unlike traditional therapies, CDT has the advantage of generating ROS and oxygen in a spatiotemporally controlled manner, without being limited by tissue depth. This is made possible by the elevated levels of H_2_O_2_ within TME, facilitated by transition metal nanoparticles.

**Figure 14:**
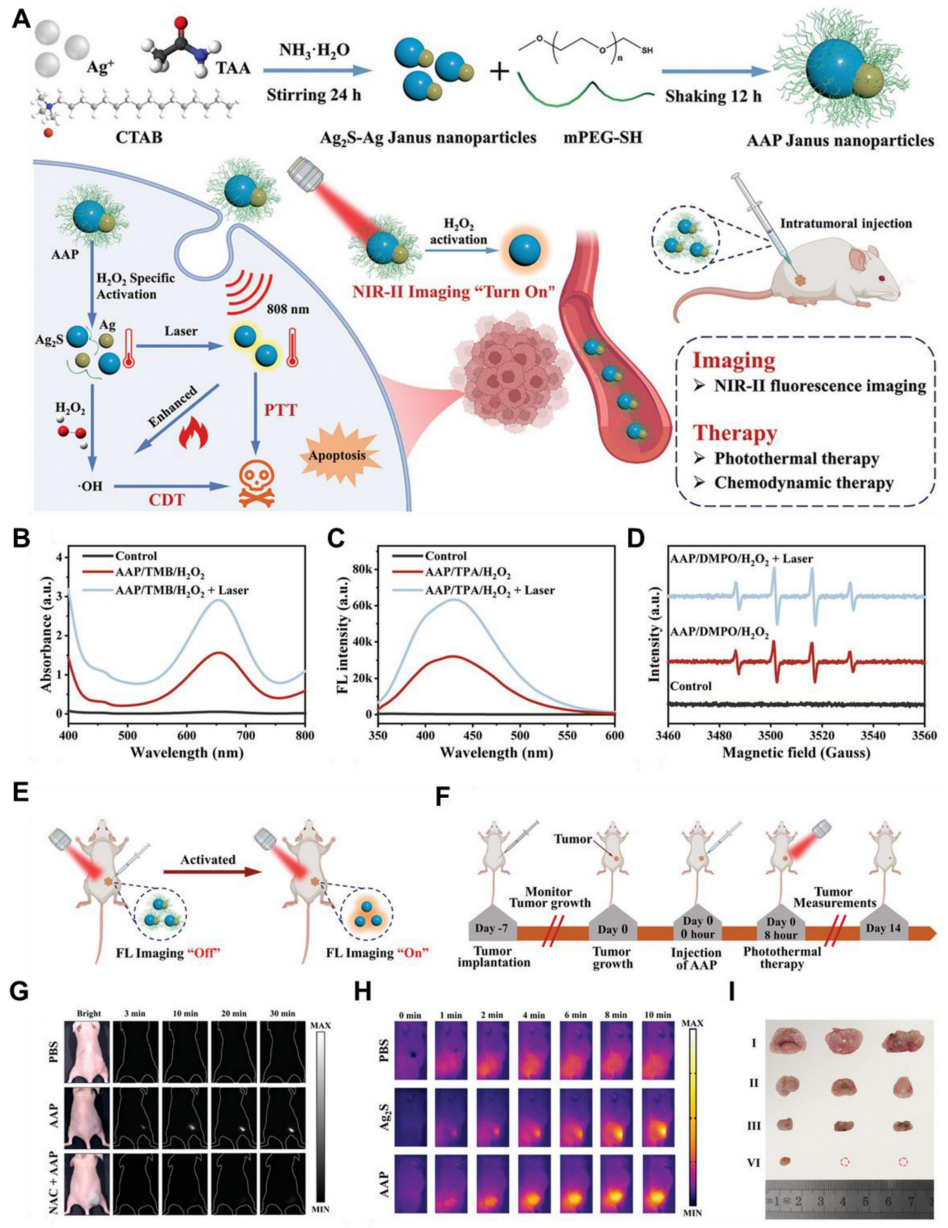
**(A)** Schematic demonstration of synthetic process of the sample and activatable NIR-II FL imaging-guided CDT and PTT combinatory therapy. **(B)** Demonstration of chemodynamic behavior of the sample in the presence of H_2_O_2_ and TMB with and without laser irradiation.** (C)** The demonstration of the photothermal behavior of the sample *via* FL spectra with and without laser irradiation.** (D)** Demonstration of ROS production efficiency of the sample in the presence of H_2_O_2_ and DMPO with and without laser irradiation. **(E)** Schematic representation of H_2_O_2_ activatable NIR-II FL imaging of the sample in tumor-bearing mice. **(F)** Schematic representation of treatment plan of the tumor-bearing mice after sample injection. **(G)** *In vivo* NIR-II FL imaging of the tumor-bearing mice after injection of the sample nanoparticles. **(H)** Demonstration of thermal imaging of the tumor area within the tumor-bearing mice after sample injection under 808 nm laser excitation having 1.0 W cm^-2^ power for 10 min. **(I)** Demonstration of treatment efficiency of the applied therapy by *ex vivo* tumor images of the tumor-bearing mice. Adopted with permission from 162, Copyrights 2024 WILEY.

However, despite its promising potential, the clinical application of CDT for GBM is challenged by several factors, including short blood circulation times, immune system alterations, BBB, and potential harm to healthy tissues due to the deficiency of efficient targeting 159. Typically, CDT agents gather in tumors through the enhanced EPR effect, but for effective treatment and diagnosis of GBM, nanoparticles need to have precise targeting, immune evasion, and BBB-crossing capabilities. Because of the delicacy of the brain, the NIR-II laser is employed for localized slight hyperthermia and is adopted as an activator of enhanced CDT, ensuring a negligible effect on the surrounding brain tissues 160. The combination of NIR-II with RENPs and QDs further improves CDT efficacy. These nanomaterials can absorb NIR light and convert it into heat or other forms of energy, which, in turn, can trigger the release of cytotoxic agents or enhance the catalytic activity of the nanomaterials, thereby boosting the therapeutic effect of CDT 161. For example, Bao *et al.* designed a straightforward method to synthesize **Ag_2_S-Ag** Janus nanoparticles coated with PEG, referred to as AAP for NIR-II FL imaging-guided tumor therapy **(Figure 14A)** 162. These nanoparticles were activated by endogenous H_2_O_2_, enabling precise NIR-II FL imaging and combined cancer therapy, and termed as activatable fluorescent probes for the treatment of subcutaneous glioma. AAP Janus nanoprobes demonstrated the ability to integrate and enhance the dual functionalities of semiconductor and noble metal components present in the sample. In addition to their capability for activatable NIR-II FL imaging **(Figure 14G)**, these probes facilitated localized hyperthermia by efficiently converting absorbed NIR light into heat for PTT applications when irradiated with an 808 nm laser **(Figure 14C and H)**. Furthermore, AAP Janus nanoparticles exhibited excellent enzyme-like catalytic activity, promoting the oxidation of H_2_O_2_ to generate •OH, which possessed strong oxidative properties because of the presence of Ag that inhibited tumor cell growth *via* CDT **(Figure 14B and D)**. The innovative design of AAP Janus probes served as versatile nanoplatforms for NIR-II FL imaging-guided combination cancer therapy of GBM, potentially offering enhanced treatment effectiveness while minimizing side effects 162.

The treatment efficiency and TME dependence of CDT suggest its robustness for tumor therapy and demonstrate its promising nature. However, CDT has several limitations that hinder its clinical application. Its effectiveness relies on the endogenous H_2_O_2_ levels in the tumor, which may be too low to generate sufficient •OH for effective treatment. Additionally, the acidic TME required for optimal Fenton or Fenton-like reactions is not uniform across all tumors, reducing the efficiency of CDT. Furthermore, some tumor cells have strong antioxidant defenses, such as GSH, that neutralize •OH and limit therapeutic outcomes. Lastly, the low catalytic efficiency of metal-based CDT agents often necessitates high doses, increasing the risk of toxicity. Therefore, for controlled ROS production and tumor treatment to avoid toxicity to the healthy tissues, there is a need to design an external stimulus-dependent therapeutic modality. The external stimulus can remotely control the therapeutic efficiency of the sample and can enhance the survival rate of the patient for better healthcare.

### 4.6. NIR-II FL imaging-guided sonodynamic therapy of GBM

Sonodynamic therapy (SDT) has been a potential therapeutic approach for glioma treatment in recent years, and studies have reported the robustness and efficiency of SDT regarding its use to treat different gliomas 163. SDT provides a non-invasive approach for precisely eliminating solid tumors by systemically delivering non-toxic chemical agents, known as sonosensitizers. These agents accumulate within tumor cells or their surrounding environment and are then activated by low-frequency ultrasound (US) to generate cytotoxic compounds, enabling targeted tumor destruction 164. The sonosensitizers can yield a large number of ROS under US excitation to eradicate cancer cells. It is also equally effective for deep-seated tumors or interior large tumors due to the enhanced penetration depth of US up to 3 cm within the body. Essentially, both sensitization and US exposure do not have anti-tumor effects themselves; instead, cytotoxic events occur when both are combined 165. For example, Jia *et al*. reported novel heterostructured nanoagents for SDT of brain glioma, integrating fluorescent downconversion nanoparticles with Fe-coordinated porphyrin MOF 166. This architecture enabled skull-penetrating NIR-IIb FL imaging for precise treatment guidance. The core-shell structure comprises the fluorescent nanoparticles encapsulated within a MOF shell, further loaded with the chemotherapeutic drug sorafenib (SRF), and functionalized with the lactoferrin (LF) ligand to facilitate BBB crossing.

These multifunctional nanoagents exhibited an average size of approximately 55 nm. Under 980 nm laser excitation, the fluorescent core emitted intense downshifting FL around 1525 nm, exploiting the superior tissue penetration of NIR-IIb light. NIR-IIb FL imaging allowed for real-time monitoring of nanoagents biodistribution, enabling precise timing for optimal treatment delivery. Upon US activation, the nanoagents triggered a cascade of ROS generation, including ^1^O_2_ and •OH. The MOF shell, containing porphyrin complexed with Fe^3+^, catalyzed the decomposition of H_2_O_2_ into O_2_ and water, mimicking the action of catalase. Furthermore, US irradiation facilitated the reduction of Fe^3+^ to Fe^2+^ within the MOF shell, promoting •OH generation *via* the Fenton reaction. The generated ROS enhances the intracellular release of SRF, an FDA-approved hepatocellular carcinoma drug. SRF disrupted GSH synthesis, depleting cellular antioxidant levels and amplifying ROS-mediated damage to tumor cells. Notably, Fe^3+^ reduction, driven by both GSH within the TME and the Fenton reaction, not only enhanced SDT efficacy but also led to the recovery of NIR-IIb FL, providing an indirect indicator of SDT-induced cellular damage. The amalgamation of SDT and CDT has produced a significant synergistic effect that has improved the therapeutic efficiency of GBM 166.

**Figure 15:**
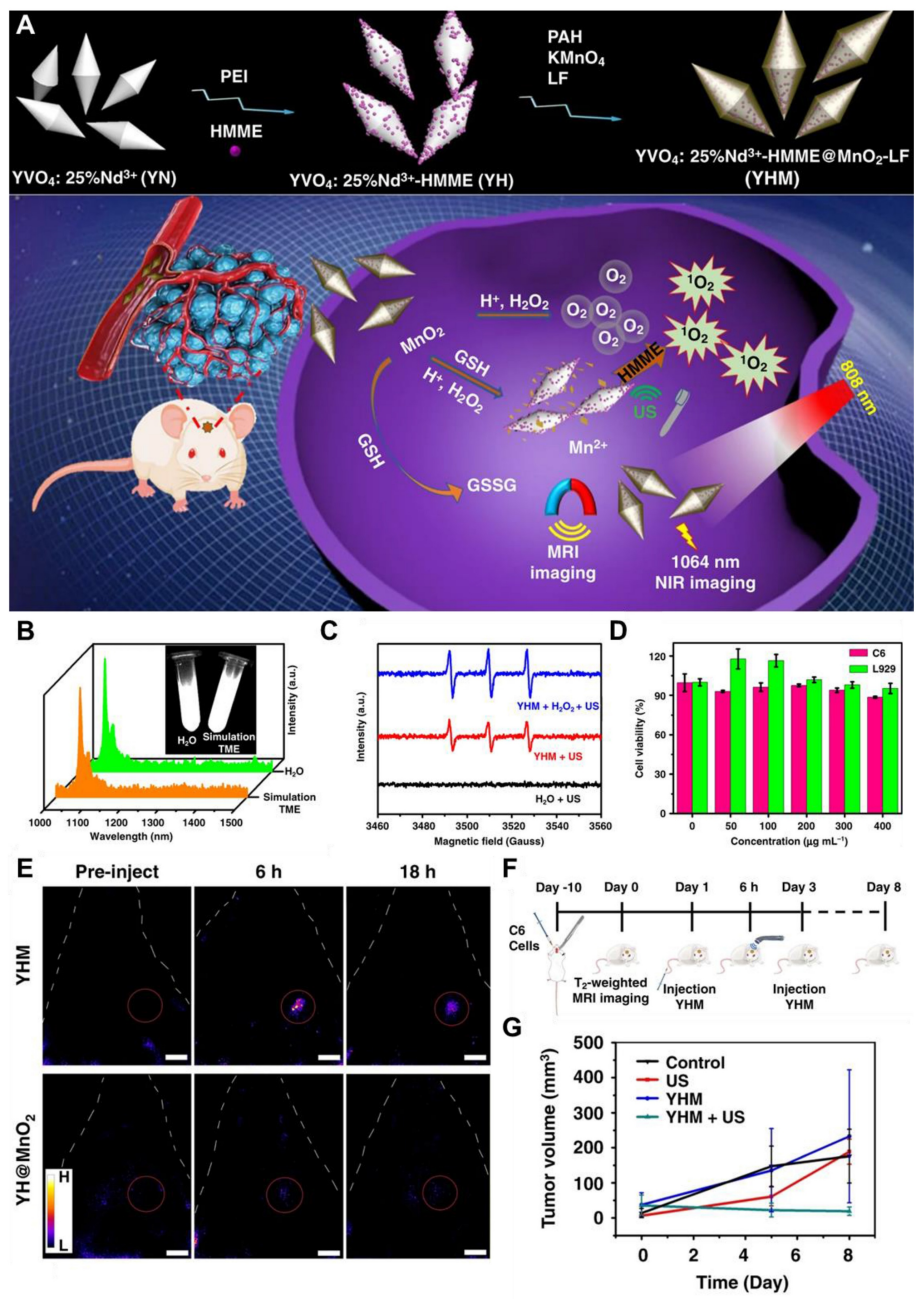
** (A)** Schematic illustration of multimodal imaging-guided TME self-enhanced SDT of GBM.** (B)** Demonstration of NIR-II FL spectra of the sample in H_2_O and simulated TME. **(C)** Demonstration of ^1^O_2_ generation detection in various conditions by ESR. **(D)** Cytotoxicity profile of the sample with different cell lines at different concentrations. **(E)** NIR-II FL imaging of orthotopic glioma-bearing mice after the tail vein injection of different samples, scale bar: 5 mm. **(F)** Demonstration of treatment and monitoring plan for tumor-bearing mice. **(G)** Demonstration of change in the tumor volume after treatment (n = 3). Adopted with permission from 34, Copyrights 2022 NATURE.

Moreover, Lv *et al*. developed a multifunctional nanotheranostic platform using **YVO_4_:Nd^3+^** nanoparticles as the core, serving as both the carrier for the sonosensitizer hematoporphyrin monomethyl ether and the foundation for a MnO_2_ nanosheet shell **(Figure 15A)** 34. This design enables NIR-II FL imaging, MRI, and effective SDT for orthotopic gliomas **(Figure 15E)**. To enhance targeting and BBB penetration, LF was added to the surface, leveraging the overexpression of lactoferrin receptors on the glioma cells and enhancing the biocompatibility of the sample **(Figure 15D)**. The resulting nanoplatforms emitted a strong FL peak at 1064 nm under 808 nm laser excitation, allowing for NIR-II FL imaging **(Figure 15B)**. Within the TME, the MnO_2_ shell catalyzes H_2_O_2_ decomposition to generate O_2_, which boosts ^1^O_2_ production under US stimulation **(Figure 15C)**, while also releasing Mn^2+^ for TME-responsive MRI. These properties highlighted the potential as powerful theranostic nanoagents for NIR-II FL imaging, MRI, and TME-enhanced SDT in orthotopic gliomas 34.

Besides all the mentioned advantages, SDT also has several limitations including low energy conversion efficiency and dependence on oxygen levels, making it less effective in hypoxic tumors. Additionally, some sonosensitizers have poor stability and bioavailability, and off-target effects may lead to unintended tissue damage and reduced therapeutic precision. Therefore, scientists should develop therapeutic modalities capable of activating the immunity of the body itself to avoid the use of external agents for solving toxicity problems.

### 4.7. NIR-II FL imaging-guided immunotherapy of GBM

**Figure 16:**
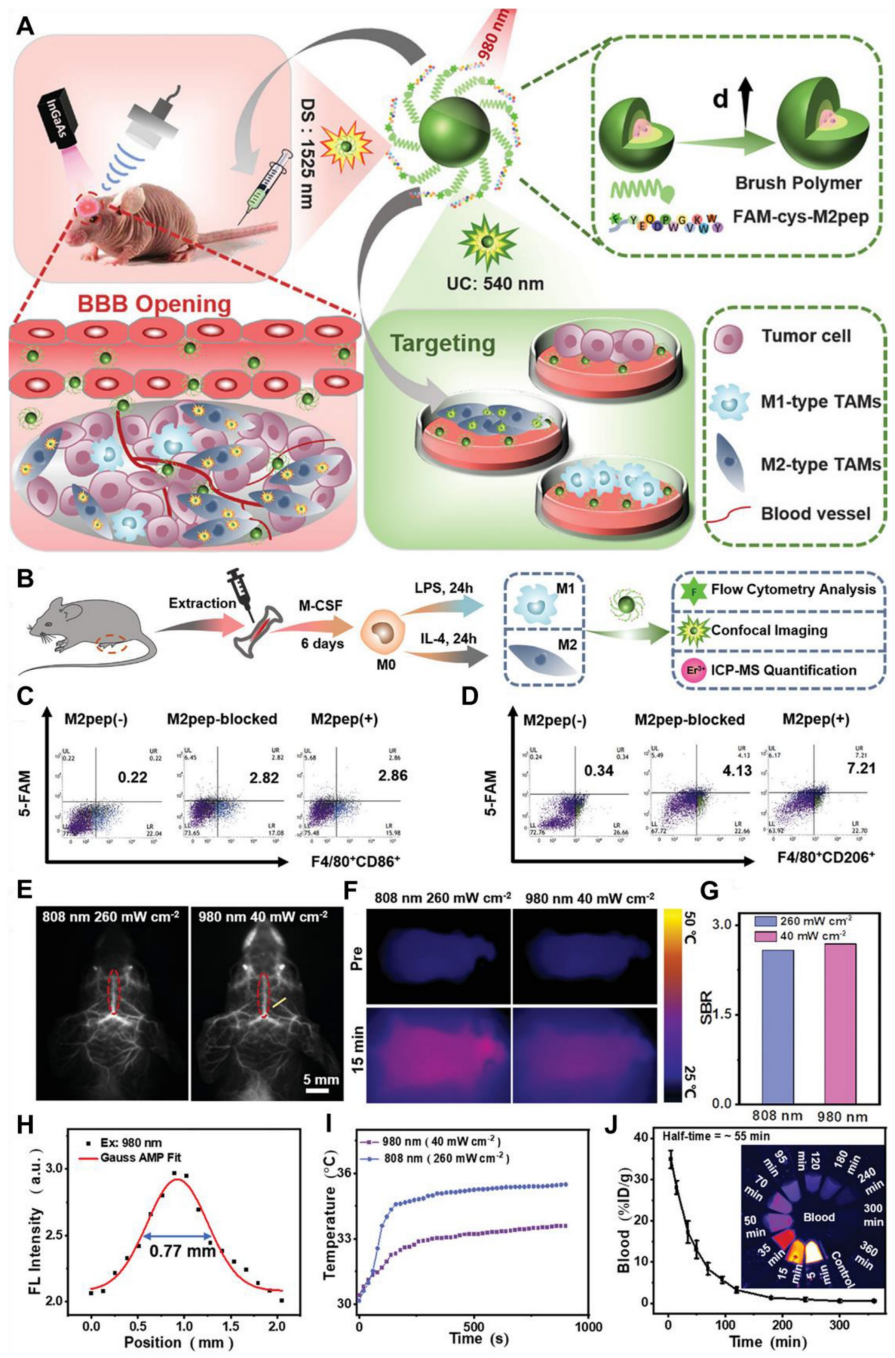
** (A)** Schematic representation of NIR-IIb FL imaging-guided targeting of M2 TAMs for *in vitro* and *in vivo* immunotherapy of orthotopic GBM. **(B)** Schematic representation of isolation, maturation, and polarization of the macrophages. **(C and D)** Demonstration of flow cytometry results of M1 and M2 macrophages when incubated with different nanoprobes with the help of F4/80^+^CD86^+^. **(E)** NIR-II FL images of brain vessels of normal mice after *i.v.* injection of the sample nanoparticles upon 808 nm and 980 nm laser excitation respectively. **(F)** Representation of infrared thermal pictures of nude mice upon 808 nm and 980 nm laser excitation for 15 minutes. **(G)** Demonstration of SBR of the NIR-IIb FL images of brain vessels.** (H)** Measurement of vessel width *via* NIR-II FL imaging.** (I)** Monitoring of temperature variation in the sample upon light irradiation.** (J)** Calculation of blood circulation half-life of the sample via NIR-II FL intensity of the sample (mean ± SD, n = 3). Adopted with permission from 169, Copyrights 2022 WILEY.

Immunotherapy has emerged as one of the most powerful anticancer strategies, extensively utilized to elicit robust and systemic antitumor immune responses. Its application in GBM treatment has attracted significant attention due to its ability to effectively eliminate cancer cells and induce immune memory, thereby reducing the chance of recurrence 23. A key approach within immunotherapy is immune checkpoint blockade (ICB) treatment, which uses monoclonal antibody-based immune checkpoint suppressors to proficiently restore the anticancer immunity cycle by rebooting cytotoxic T lymphocytes (CTLs). As regulators of the immune system, immune checkpoints maintain a balance between inhibitory and costimulatory signals. This equilibrium protects tissues from damage caused by excessive immune responses and prevents autoimmunity. Despite its promise, the efficacy of immunotherapy against GBM is significantly compromised by the immunosuppressive TME and the formidable barrier presented by BBB 31. GBM, as an immunologically cold tumor, exhibits a TME characterized by low numbers of tumor-infiltrating lymphocytes and high levels of immunosuppressive cells. Additionally, the activities of effector immune cells, including dendritic cells, CTLs, and antitumor macrophages, are suppressed within this environment 167. The effectiveness of immunotherapy largely depends on altering the delicate balance between effector T cells and immune suppressor cells. Although immunotherapy has the potential to produce remarkable results, the overall response rate remains relatively low, primarily due to the absence of T cell infiltration in tumors. The efficacy of immunomodulatory strategies is now widely acknowledged to depend on the presence of an initial immune response and the activation of pre-existing immunity 203. There is broad agreement on the crucial role of effector T cells in antitumor responses. Advances in technology, analytical methods, and immunological mechanisms have made it possible to identify patients who are more likely to benefit from immunotherapy. Cancer immunotherapy is based on three key principles: first, evidence of immune surveillance demonstrated through immune-deficient mouse models; second, the recognition that pre-existing immunity and naturally occurring intratumoral T cells play a vital role in human cancers; and third, the enhancement of this pre-existing immunity by blocking checkpoint receptors on T cells. NIR-II imaging has become a valuable tool for visualizing FL-labelled immune cells and monitoring immune responses using reflectance-based or microscopic imaging systems in cancer immunotherapy 168. Since cell-based treatments can be applied throughout the body, it is often necessary to visualize the macroscopic level of the area of interest to obtain exclusive and precise imaging. Zhu *et al*. explored a novel approach for targeted immunoimaging of tumor-associated macrophages (TAMs) in orthotopic GBM using NIR-IIb fluorescent nanoprobes **(Figure 16A)** 169. GBM, a highly malignant brain tumor, is difficult to treat due to its immunosuppressive TME, in which M2-type TAMs play a significant role in promoting tumor growth and resistance to therapy. They developed Er-based RENPs with M2pep polypeptide for precise imaging of M2-type TAMs *in vivo* **(Figure 16C and D)**. These nanoprobes emitted strong NIR-IIb FL beyond 1500 nm when excited by a low-power 980 nm laser, allowing deep-tissue imaging with minimal background interference **(Figure 16E)**. To enhance their imaging performance, the nanoprobes were optimized by modifying their core-shell structure and doping with rare-earth elements. *In vivo* experiments demonstrated that these functionalized nanoprobes effectively targeted M2-type TAMs, as confirmed by co-localization with immunofluorescence staining in tumor tissues after crossing the BBB. The FUS technique was applied to open the BBB for sample penetration and targeting the M2-type TAMs. The study also investigated the biodistribution and clearance of the nanoprobes, showing predominant accumulation in the tumor and gradual elimination *via* the liver and spleen. Overall, the study highlighted the potential of NIR-IIb fluorescent nanoprobes as a powerful tool for noninvasive, tumor-specific imaging and tracking of immune responses in GBM 169.

Moreover, immunotherapy can also work synergistically with other treatments, enhancing their effectiveness. It can be combined with chemotherapy, radiation, or targeted therapy to create a more comprehensive treatment approach. For example, Yalamandala *et al.* designed a self-cascade penetrating brain tumor immunotherapy mediated by NIR-II cell membrane-disrupting nano-flakes. The designed system consisted of a membrane-disrupting polymer (mPEG-b-C18) loaded with CuS nanoflakes, termed CuS nanospheres (CuS NBs). CuS NBs underwent charge conversion at the brain tumor site by using a convection-enhanced delivery (CED) system and making it easier for them to penetrate deep into the tumor by loosening cell-to-cell connections in the slightly acidic environment. Additionally, CuS also induced NanoEL at the tumor site. Then, CuS NB accumulated in the brain tumor and was distributed in a deep tumor area upon low-power NIR-II irradiation (0.8 W/cm^2^). At the tumor site, NIR-II hyperthermia through CuS NB promoted cancer cell apoptosis and the release of tumor-associated antigens (TAA). These TAAs are then captured by primary amine groups on NB, which also serve as a transporter of antigens for immunogenic cell death. The *in situ* capture system further promoted the retention of antigen release to achieve sustained immune stimulation and brain tumor suppression. The captured antigen further recruited more dendritic cells to enhance the immune response of CD4^+^ andCD8^+^ T cells. Therefore, the proposed antigen capture mechanism demonstrated great potential for application in enhancing cancer immunotherapy 176,204. Furthermore, some immunotherapies, like immune checkpoint inhibitors, can reprogram the TME, making "cold" tumors more responsive to treatment. Overall, immunotherapy represents a transformative approach to cancer treatment, offering specificity, durability, and improved survival outcomes, particularly for patients with aggressive or previously untreatable gliomas. Its ability to personalize treatment further enhances its clinical potential and it can be the ultimate therapeutic modality in the near future.

## 5. Challenges and Perspectives

The application of NIR-II FL-based imaging-guided therapy of GBM is a promising theranostic strategy, but it also has significant challenges. Therefore, there is a pressing need to work in scientific, regulatory, and clinical domains, requiring innovative solutions to bridge the gaps between laboratory research and clinical implementation for better healthcare. In this section, key hurdles and potential strategies have been discussed to offer a comprehensive perspective on the future of NIR-II fluorescent materials in imaging-guided therapy of GBM.

### 5.1. Achieving clinical translation: from bench to bedside

Clinical translation of NIR-II fluorescent nanoprobes is a complex journey that requires thorough scientific validation and strict regulatory requirements. Regulatory agencies like the FDA and EMA demand extensive preclinical and clinical data to confirm that these materials are safe, effective, and reliable for clinical translation.

Currently, NIR-II fluorescent nanoprobes are under clinical and preclinical trials for NIR-II imaging-guided therapy of GBM. For example, Shi *et al.* 2022 evaluated the capabilities and clinical efficacy of NIR-II fluorescence-guided surgery (FGS) for the removal of human glioma, using an in-house designed multispectral imaging setup and an FDA-approved **ICG** NIR-II fluorescent probe 182. The NIR-II FGS showed excellent biosafety, higher sensitivity, and real-time tumor localization, with the ability to detect deep-seated tumors due to reduced photon scattering and absorption. The study found a 100% detection rate for GBM patients and significantly higher complete resection rates. The FGS group also showed significantly prolonged progression-free survival and overall survival compared to the white light surgery group. Notably, NIR-II FGS outperformed traditional ICG-based NIR-I FGS, **5-ALA**, and FS-based FGS in complete resection rates and survival outcomes 143. The technique enabled more complete tumor resections, which is linked to improved survival rates.

**Figure 17:**
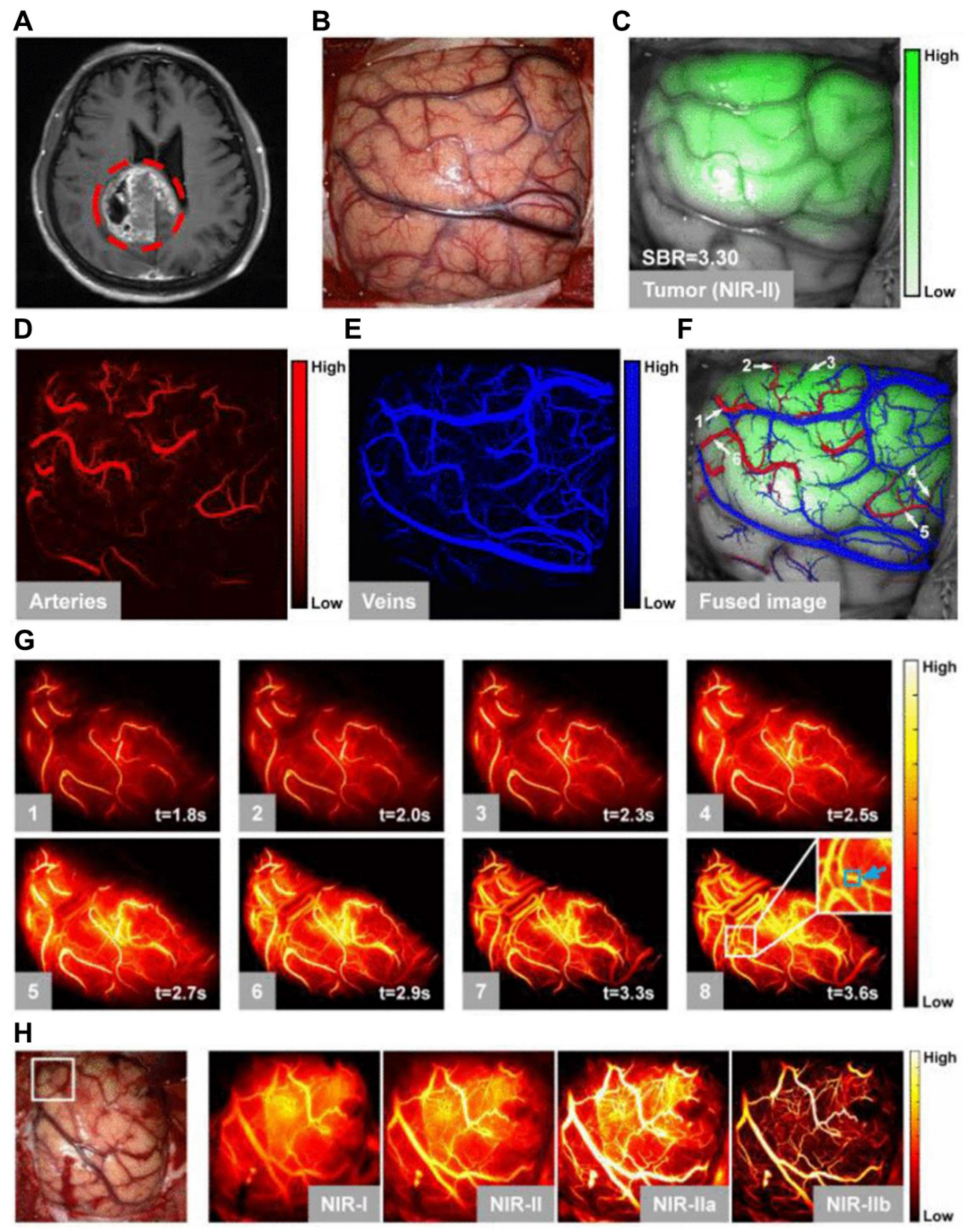
** (A)** MRI-based identification of the brain tumor; the red circle indicates a tumor lesion in the right parietal.** (B)** Visible light imaging of brain vasculature.** (C)** NIR-II FL imaging of the tumor site with an SBR of 3.30.** (D)** NIR-IIb FL imaging of the blood supplying arteries.** (E)** NIR-IIb FL imaging of venial system after injecting ICG.** (F)** Fused imaging-based demonstration of tumors, arteries, and veins. White arrows indicated the blood-suppling arteries of the tumor. **(G)** Demonstration of real-time NIR-II FL imaging-based blood flow visualization of a female patient. (H) Comparison of multispectral FL images of the cerebral vessels demonstrating the superior penetration ability of NIR-IIb FL imaging. Reused under Creative Commons Attribution License 183.

While the study demonstrated no significant toxicity at a reduced **ICG** dose (1 mg/kg), it has also acknowledged the limitation of non-specific FL uptake by adjacent tissues, suggesting the need for more targeted NIR-II probes in future studies. Moreover, Cao *et al.* in 2022 successfully demonstrated the use of NIR-IIa/IIb multispectral FL imaging in the resection of grade III/IV gliomas 183. By combining the FDA-approved **ICG** probe, the technology allows for precise visualization of tumor-feeding arteries and vascular boundaries during surgery **(Figure 17)**. Seven patients with glioma were enrolled, and all tumors were successfully resected without major bleeding, due to the ability to block tumor-feeding arteries precisely by clear visualization with the help of NIR-II FL. The volume of blood loss in the NIR-II FL imaging group was significantly smaller compared to the control group. Additionally, the NIR-II FL imaging provided superior spatial resolution, contrast, and vessel tracking compared to conventional NIR-I FL imaging. With a real-time imaging speed of 50 fps, the technique allowed for accurate monitoring of blood perfusion and vessel dilation. NIR-II FL imaging achieved a significantly higher tumor SBR than NIR-I, improving tumor delineation. The NIR-IIb spectrum, particularly for vascular imaging, showed the highest performance with a capillary resolution of 180 μm. The discussed approach in NIR-IIa/IIb FL imaging has offered an advanced, precise method for glioma surgery, enabling accurate tumor resection while minimizing damage to surrounding brain tissue 183. Moreover, the NIR-II fluorescent materials must also outperform existing imaging methods like MRI and CT by offering deeper tissue penetration, better resolution, and enhanced contrast. These advantages are necessary to justify their use in clinical practice instead of the already in-use medicines 205. Lastly, cost and accessibility are significant hurdles. The high expense of producing NIR-II fluorescent materials and the specialized equipment needed for imaging might limit their widespread use. Finding cost-effective ways to synthesize these materials and develop accessible imaging systems will be key to making these technologies available to a broader range of patients. However, the biggest challenge for the research community is designing a standardized, reproducible synthetic route for inorganic/hybrid NIR-II fluorescent materials like QDs, RENPs, SWCNTs, CDs, and MNCs. The batch-to-batch variation in the current synthetic technique can affect the consistency and performance of these materials during the diagnostic and therapeutic process. Researchers need to develop scalable, reproducible manufacturing processes that meet the high standards required for clinical use to improve patient healthcare.

**Figure 18:**
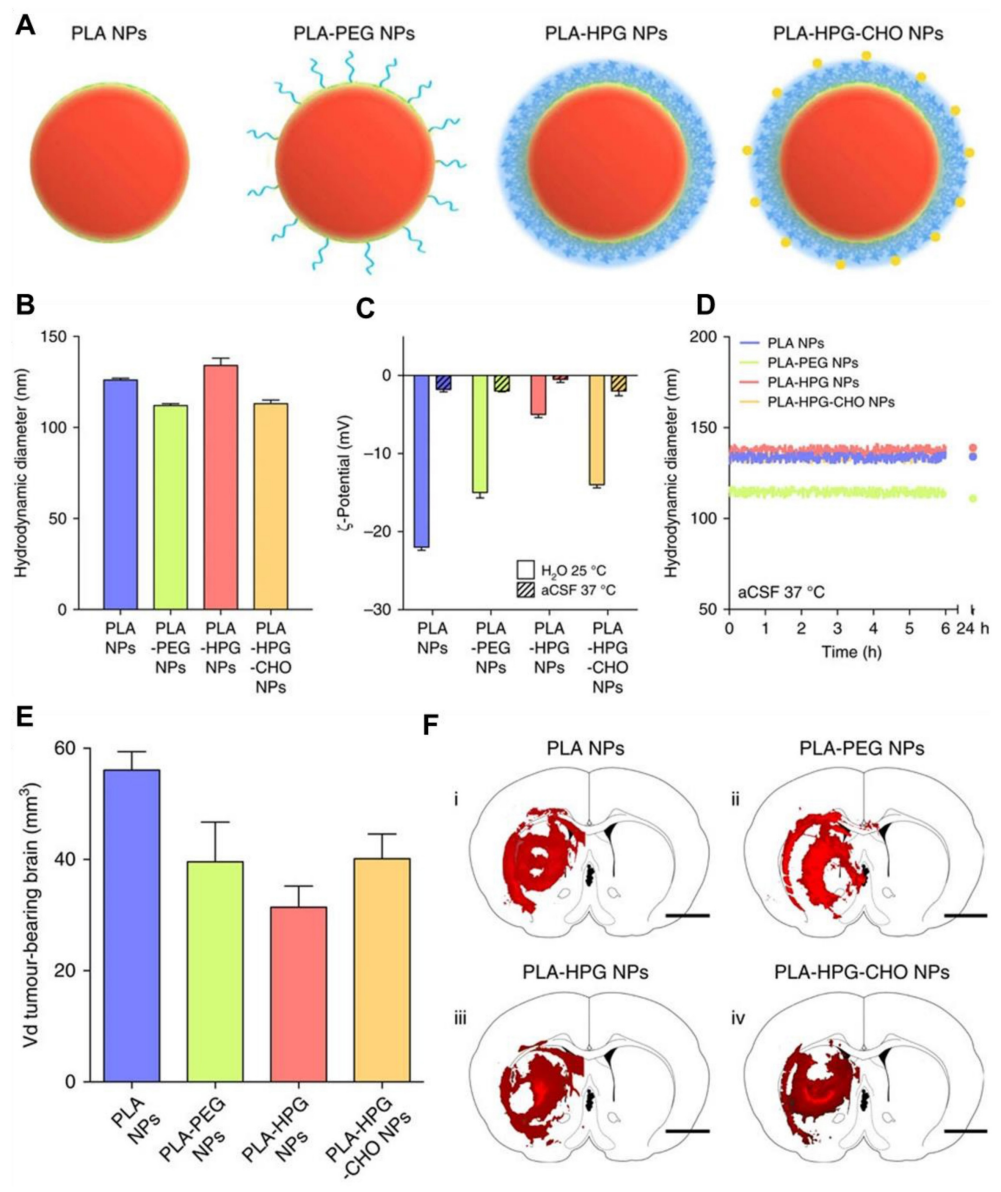
** (A)** Graphical representation of PLA-based nanoparticles with various surface coatings (PEG, HPG, and HPG-CHO).** (B)** Demonstration of size analysis of the nanoparticles coated with different coatings dispersed in water.** (C)** Measurement of zeta potential in water at 25 °C and in artificial cerebrospinal fluid at 37 °C demonstrated surface neutralization of the particles in artificial cerebrospinal fluid. **(D)** Demonstration of particle stabilization in CSF at 37 °C up to 24 hours without aggregation. **(E)** Nanoparticle distribution study in RG2 tumor-bearing mice. **(F)** Distribution pattern of the nanoparticles in tumor-bearing brains representing the effect of coating in BBB penetration. Adopted with permission from 184, Copyrights 2017 NATURE.

Furthermore, biocompatibility is another critical issue for inorganic/hybrid NIR-II fluorescent materials because of the presence of heavy metals like lead or cadmium, raising concerns about long-term toxicity. It is also essential to conduct thorough toxicology studies to assess the safety of these materials, including their behavior in the body, how they are processed, and whether they might accumulate in important organs. One strategy to achieve safe clearance is designing nanoparticles with optimal sizes and surface properties. Surface modifications, such as PEGylation or zwitterionic coatings, can enhance circulation time and reduce immune clearance, thereby minimizing toxicity. Moreover, biodegradable materials offer another promising approach. Carbon-based nanomaterials like CDs and SWCNTs naturally degrade over time, reducing the risk of long-term accumulation. However, materials like RENPs and MNCs may require additional engineering to enhance their degradation profiles. For example, enzyme-responsive or pH-sensitive nanoparticles can be designed to break down in specific physiological conditions, facilitating their safe metabolism and excretion. For example, Song *et al.* explored the impact of surface chemistry in the brain/tumor cellular microenvironment using PLA-based nanoparticles with varying coatings: PLA, PLA-PEG, PLA-HPG, and PLA-HPG-CHO **(Figure 18A)** 184. Among these, PLA-HPG-CHO exhibited the highest tumor tropism and cellular uptake. The bioadhesive aldehyde groups of PLA-HPG-CHO enhanced tumor cell interaction and absorption, with uptake percentages rising significantly after 24 hours **(Figure 18D)**. Comparatively, PLA-PEG and PLA-HPG nanoparticles displayed lower cellular internalization, while PLA-HPG-CHO nanoparticles showed preferential uptake by microglial cells, reducing interaction with neurons and astrocytes. Despite the advantages of PEG, its limitations include poor biodegradability and potential immune responses. HPG coatings showed improved stealth properties and tumor specificity over PEG. Cellular uptake studies revealed that PLA-HPG-CHO nanoparticles achieved the highest absorption levels, demonstrating superior efficacy for tumor-targeted drug delivery 184.

Therefore, comprehensive biodistribution studies are essential to understand how nanoparticles accumulate in different organs and tissues. Long-term toxicology assessments can identify potential risks and guide the design of safer materials. By addressing these factors, researchers can develop NIR-II fluorescent materials that are not only effective for imaging and therapy of GBM but also safe for long-term use in patients.

### 5.2. Overcoming the BBB: targeting and immunotherapy

**Figure 19:**
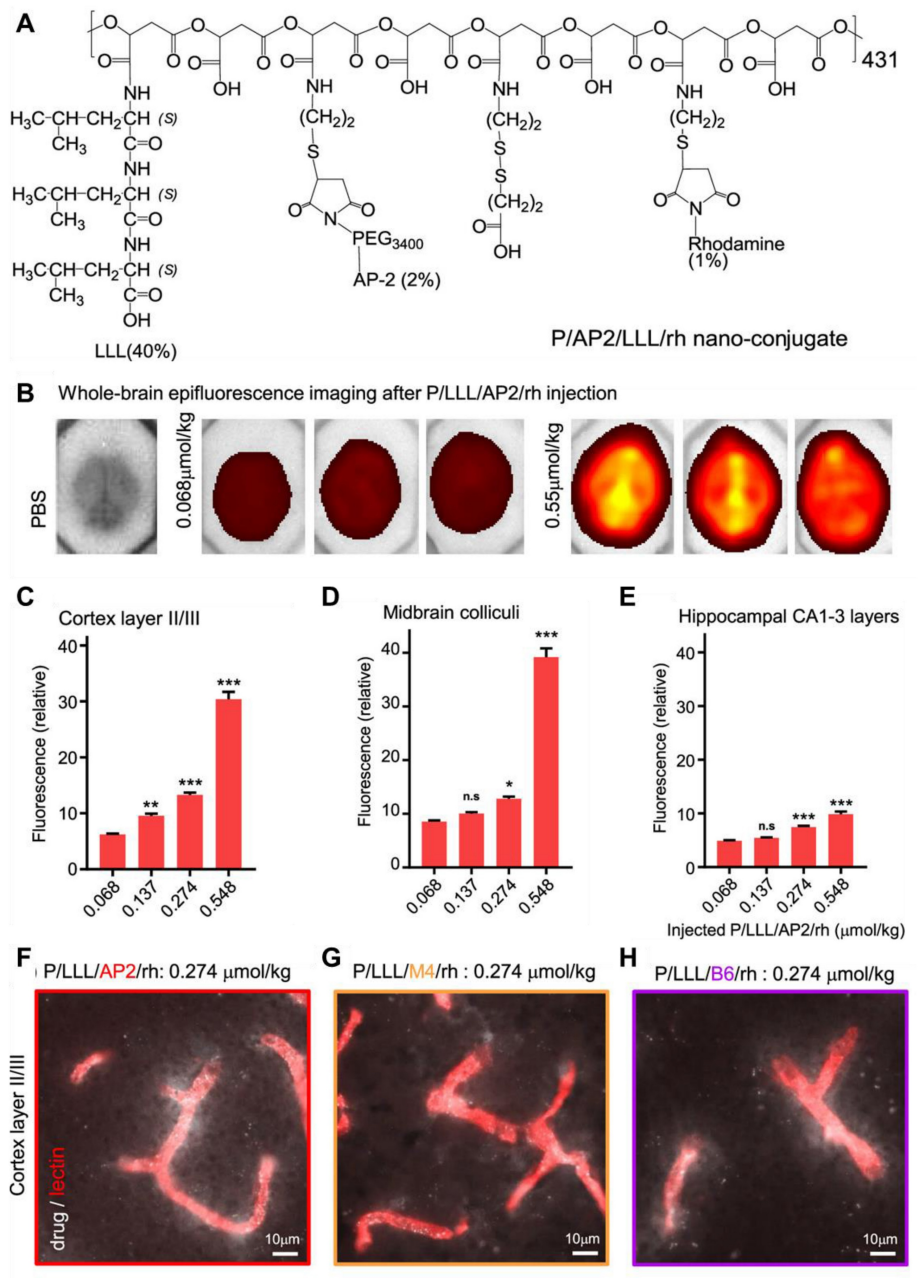
** (A)** Chemical composition of the sample nanoconjugate for BBB penetration.** (B)** Whole brain epifluorescence imaging by injecting different concentrations (PBS, 0.068, and 0.55 µmol/kg) of sample to demonstrate the concentration-dependent BBB penetration of the probe. **(C-D)** The average FL intensity of the nanoconjugate in the brain parenchyma was measured by injecting four different drug concentrations in μmol/kg. FL imaging exhibiting rh-labeled nanoconjugate penetration of the BBB by **P/LLL/rh** combined to AP2 **(F)**, M4 **(G)**, and B6 **(H)**. Adopted with permission from 188, Copyrights 2019 ACS PUBLICATIONS.

The BBB is a major obstacle in GBM treatment, limiting the delivery of therapeutic agents to the tumor site. Strategies to enhance BBB penetration and tumor targeting include biomimetic coatings and active targeting ligands. Biomimetic coatings, which involve cloaking nanoparticles with natural membranes derived from cancer cells, macrophages, or exosomes, can enhance immune evasion and BBB penetration. These coatings also improve targeting specificity, ensuring that nanoparticles reach the tumor site. Active targeting ligands, such as transferrin or integrin-binding peptides, can further enhance BBB penetration and tumor targeting. These ligands bind to receptors overexpressed on GBM cells, enabling precise delivery of therapeutic agents. Combining these strategies with T cell-based immunotherapies, such as CAR-T and NK cell therapies, can enhance both targeting and immune activation, offering a powerful approach to GBM treatment. For example, Israel *et al.* engineered a biocompatible and biodegradable β-poly(l-malic acid) (PMLA, also referred to as P) to serve as a platform for crossing the BBB 188. They chemically conjugated it with Angiopep-2 (AP2), MiniAp-4 (M4), transferrin receptor ligands (TfL), and B6 **(Figure 19F-G)**. Furthermore, a trileucine (LLL) endosome and rhodamine (rh) was conjugated to the PMLA backbone **(Figure 19A)**. The drug release profile, BBB penetration, and biological distribution of these nanoprobes were evaluated across various brain sections and different time frames using FL imaging. Among the tested formulations, the nanoconjugate **P/LLL/AP2/rh** exhibited strong FL in cortical layers II/III, the midbrain colliculi, and hippocampal CA1-3 cellular layers within 30 minutes of a single *i.v.* injection, with clearance occurring after 4 hours **(Figure 19C-E)**. In contrast, nanoconjugates lacking **AP2 (P/LLL/rh)** or **LLL (P/AP2/rh)** displayed significantly reduced BBB penetration. The LLL unit contributed to nanoconjugate stability, while AP2 enhanced BBB crossing. Additionally, nanoconjugates containing M4, cTfRL, or B6 demonstrated limited or inconsistent brain parenchyma penetration, likely due to decreased trans-BBB transport **(Figure 19F-H)**. The optimized nanoconjugate, **P/LLL/AP2/rh**, holds the potential for functionalization with intra-brain targeting and therapeutic agents for addressing molecular pathways involved in neurological disorders 188.

### 5.3. Pushing emission wavelengths beyond 1500 nm

**Figure 20:**
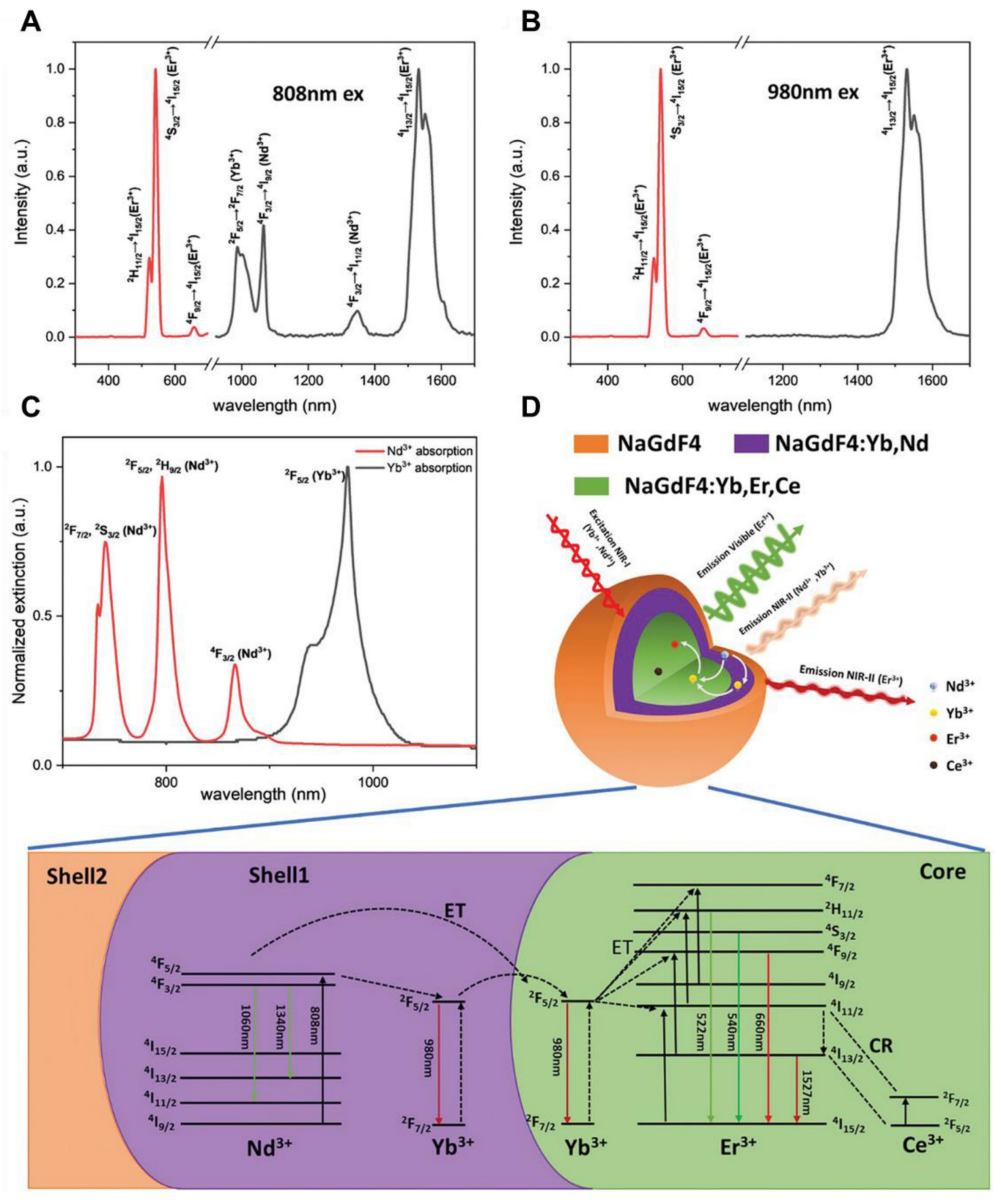
** (A, B)** Demonstration of FL spectra of the sample solution upon 808 and 980 nm laser irradiation.** (C)** Absorption spectra of aqueous solution of Nd^3+^ and Yb^3+^. **(D)** Schematic representation of energy transfer mechanisms and FL emission of the sample beyond 1500 nm upon NIR laser excitation. Adopted with permission from 185, Copyrights 2023 WILEY.

Achieving emission wavelengths above 1500 to 1700 nm is a tough but rewarding challenge for deep tissue imaging in the medical realm. Longer wavelengths help to reduce tissue scattering and autofluorescence, leading to better resolution and contrast in images. This can be achieved by modulating the electronic properties of the NIR-II fluorescent materials like RENPs. By doping RENPs with elements such as Er, Yb, or Nd, their emission can be shifted to the NIR-IIb range (1500-1700 nm). Moreover, the use of core-shell structures like **PbS/CdS** QDs can improve emission efficiency at these longer wavelengths by modulation of the bandgap. Furthermore, designing ultra-thin SWCNTs with carefully controlled lengths, allowing them to emit in the NIR-IIb range could be an efficient strategy. Surface passivation techniques can make these nanotubes brighter and more stable. The combination of NIR-II emitters with plasmonic nanomaterials or hybrid organic-inorganic structures could also be a promising approach. For example, pairing RENPs with Au nanoparticles can boost their FL, making it easier to penetrate tissues and detect tumors like GBM more accurately. Moreover, Xu *et al.* discussed the light amplification of RENPs-based core-shell-shell DCNPs in the NIR-II biological window *via* plasmonic nanostructures **(Figure 20)** 185. The FL from Yb^3+^, Nd^3+^, and Er^3+^ emissions was enhanced by 1.6, 1.7, and 2.2 times respectively, when the core-shell-shell DCNPs were joined with an Au hole-cap nanoarray under 808 nm laser excitation. Additionally, the Er^3+^ induced 1527 nm FL under 980 nm laser illumination experiences up to a 6 times enhancement. To explore the working mechanism behind the observed enhancement, the plasmonic variation of Er^3+^-induced NIR-II FL at 1550 nm under 980 nm illumination was analyzed through FDTD simulations and lifetime measurements. The results indicated that the FL enhancement was due to an amalgamation of enhanced illumination and an increased radiative decay rate. Therefore, the FY of the materials can be pushed beyond 1500 nm by adopting the mentioned strategy for better visualization results and deeper penetration into the biological tissues 185.

Advanced synthesis methods, such as hydrothermal or microwave-assisted techniques, allow researchers to fine-tune these materials to achieve the perfect emission wavelengths, pushing the limits of NIR-II FL imaging and offering new opportunities for GBM diagnosis and treatment.

### 5.4. Dual imaging and immunotherapy: a synergistic approach

The combination of NIR-II FL imaging with immunotherapy represents a powerful strategy for GBM treatment. Inorganic/hybrid nanomaterials doped with elements such as Cu, Zn, Mg, Mn, and Fe can serve as dual imaging contrast agents and immune modulators. These materials can offer unique opportunities to enhance both diagnostic accuracy and therapeutic efficacy. Mn- and Fe-doped inorganic/hybrid materials could be promising candidates for dual imaging and immunotherapy. These materials will not only enhance MRI contrast but also promote immunomodulation. By modulating the TME, these materials will enhance the efficacy of immunotherapies, such as immune checkpoint inhibitors and cytokine therapies. For example, Zhao *et al*. designed a targeted nanoplatform to modify the immunosuppressive TME with Au nanorods (GNRs), which were first coated with SiO_2_ and then MnO_2_, forming **GNRs@SiO_2_@MnO_2_ (GSM)** 186. The surface of GSM was further coated with a membrane from myeloid-derived suppressor cells (MDSCs), resulting in **GNRs@SiO_2_@MnO_2_@MDSCs (GSMM)**. The mentioned design allowed GSMM to inherit the active targeting properties of MDSCs towards the TME. The MnO_2_ coating enhanced the localized surface plasmon resonance of GNRs in the NIR-II window, enabling NIR-II photothermal imaging and photoacoustic imaging of GSMM. In addition, the release of Mn^2+^ in the acidic TME allowed for MR imaging. Inside cancer cells, Mn^2+^ catalyzed the conversion of H_2_O_2_ into •OH, triggering CDT and activating the cGAS-STING pathway. This also directly stimulated STING, leading to the secretion of type I interferons, pro-inflammatory cytokines, and chemokines. Furthermore, PTT and CDT-induced immunogenic cell death of tumor cells promoted anti-tumor immunity by exposing CRT, HMGB1, and ATP. Overall, the designed nanoplatforms offered multimodal cancer imaging and dual immunotherapy 186.

**Figure 21:**
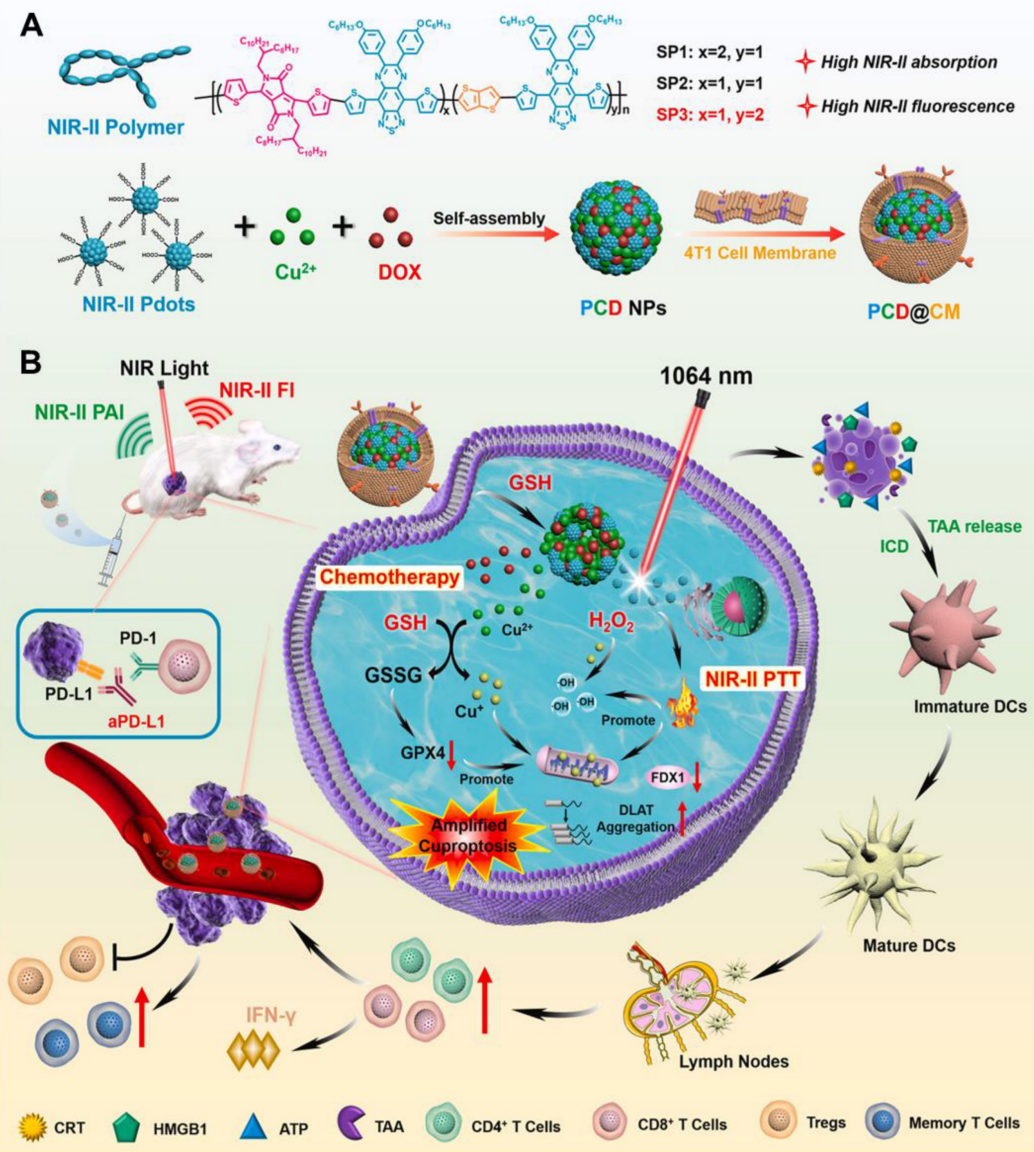
Schematic representation of the biocompatible self-assembly cuproptosis booster for synergistic cancer immunotherapy. Adopted with permission from 187, Copyrights 2024 ELSEVIER.

Moreover, Cu and Zn-based inorganic/hybrid materials can offer another avenue for imaging-guided immunotherapy. These materials can induce oxidative stress in tumor cells, triggering immunogenic cell death (ICD). By functionalizing these nanoparticles with immune checkpoint inhibitors, such as PD-L1 antibodies, researchers can further enhance their immunotherapeutic potential. For instance, Dai *et al.* synthesized an efficient NIR-II semiconducting polymer by using dual-donor engineering 187. This was followed by the creation of a biomimetic cuproptosis amplifier through Cu^2+^-based coordination self-assemblage of NIR-II fluorescent nanoscale polymer dots and DOX, which was then coated with cancer cell membranes for efficient targeting **(Figure 21)**. After targeted delivery to tumor cells, the enhanced levels of GSH within the TME trigger the disassembly of the amplifier, reducing Cu^2+^ to Cu^+^ and releasing the therapeutic agents. This enables NIR-II FL/photoacoustic imaging-guided PTT and chemotherapy. The excreted Cu^+^ initiates the accumulation of mitochondrial lipoproteins and the loss of Fe-S based proteins, resulting in high proteotoxic stress and ultimately causing cuproptosis 206. NIR-II FL triggered PTT and GSH deletion making the cancer cells extra vulnerable to cuproptosis. The enhanced cuproptosis activates immunity, leading to ICD and promoting T lymphocyte penetration, along with aPD-L1-facilitated immune checkpoint blockade. The mentioned study presented a novel approach to formulate cuproptosis-sensitizing systems activated by NIR-II phototheranostics, with homologous simultaneous targeting and anticancer immune action abilities.

Furthermore, Mg-base nanoparticles exhibit unique anti-inflammatory properties that can modulate the TME, making it more conducive to immune activation. These materials can be combined with other immunotherapies, such as CAR-T cell therapy, to enhance their effectiveness. The integration of imaging and immunotherapy into a single nanoplatform can offer several advantages real-time monitoring of therapeutic responses, allowing clinicians to adjust treatment protocols as needed, enhancing target specificity, minimizing off-target effects and reducing systemic toxicity, and creating a synergistic effect to achieve better outcomes 207. For example, NIR-II imaging can guide the delivery of immunotherapeutic nanoparticles to the tumor site, ensuring precise targeting and maximizing therapeutic efficacy. By combining imaging with immunotherapy, researchers can develop multi-functional nanoparticles that not only detect GBM but also actively combat it. This approach represents a paradigm shift in cancer treatment, offering new hope for patients with this devastating disease.

### 5.5. Smart NIR-II fluorescent inorganic/hybrids: AI-driven approaches

**Figure 22:**
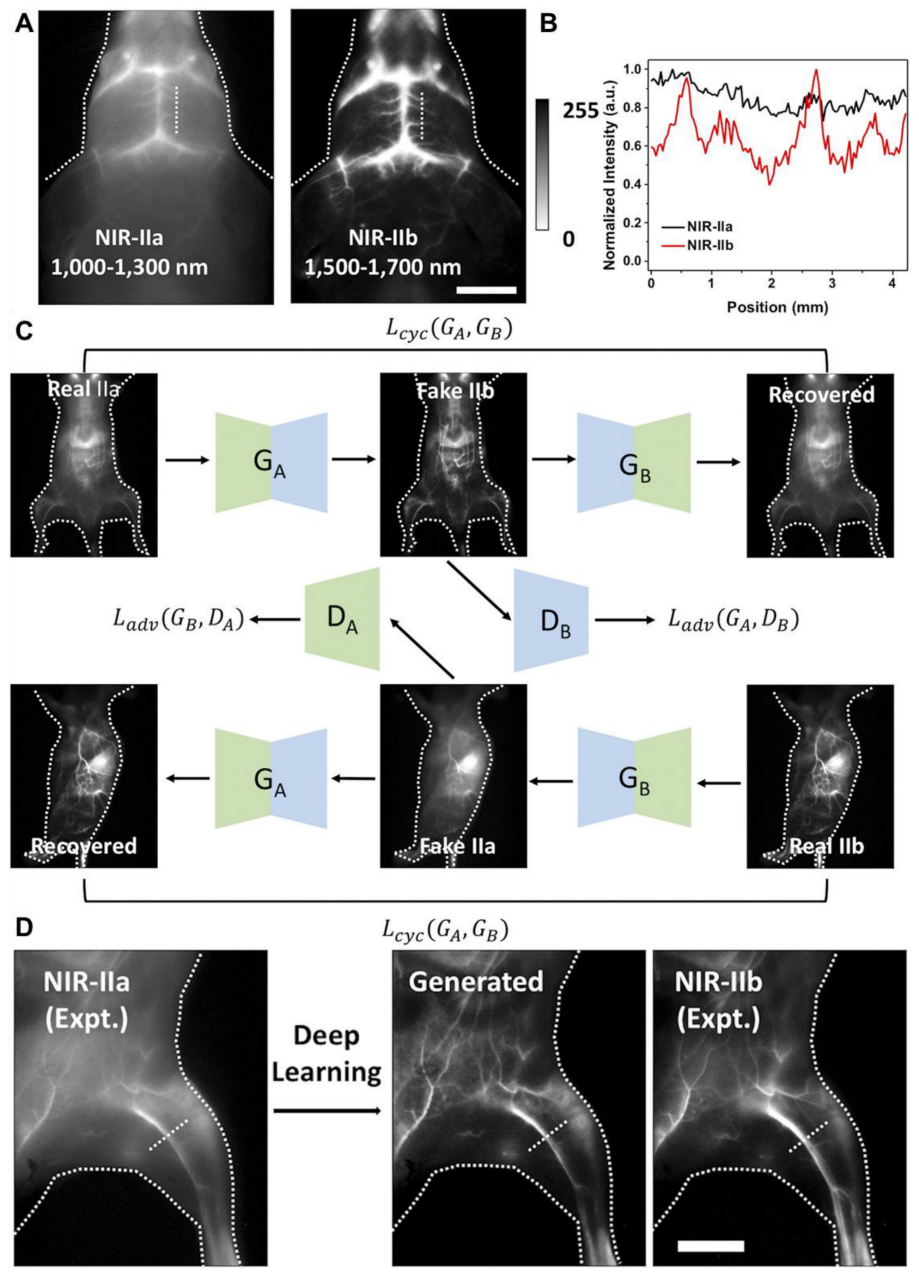
Demonstration of CycleGAN-based NIR-IIa to NIR-IIb image translation. **(A)** Comparison of NIR-IIa and NIR-IIb images in mice injected with p-FE and QDs upon 808-nm laser excitation (1000-nm long-pass and 1200-nm short-pass filters for NIR-IIa image, while 1500-nm long-pass filter for NIR-IIb image). **(B)** Cross-sectional intensity profiles of the images in both the NIR-IIa and NIR-IIb windows. **(C)** Demonstration of CycleGAN model training process. A random NIR-IIa image was processed by generator G_A_ to create an NIR-IIb image, which was then input to generator G_B_ to reconstruct the original NIR-IIa image. A discriminator D_B_ distinguished real from generated NIR-IIb images, with the overall loss being a weighted sum of adversarial and cycle consistency losses to ensure accurate image translation. **(D)** *In vivo* FL imaging of mice injected with p-FE and QDs, where the NIR-IIa image was processed by generator G_A_ to produce a contrast-enhanced image (Scale bar = 5 mm). Reused under Creative Commons Attribution License 193.

The integration of artificial intelligence (AI) with NIR-II fluorescent inorganic/hybrid materials has the potential to revolutionize the field of GBM theranostics by enabling enhanced precision, faster diagnosis, and more effective therapeutic strategies. AI can serve as a powerful tool in advancing the development and application of these materials, optimizing material design, improving imaging quality, and personalizing treatment approaches for GBM patients. AI algorithms can analyze large datasets from NIR-II FL imaging, identifying tumor regions with greater accuracy than traditional methods, and AI-driven predictive models can aid in real-time monitoring of tumor progression, allowing for dynamic adjustments in therapeutic strategies 189. In this context, deep learning, particularly deep neural networks (DNNs), has received significant attention for its efficacy in data analysis 208. DNNs also known as multilayer perceptrons, consist of neural networks with multiple hidden layers and nonsingle outputs, which interconnect through linear weight computations and the application of nonlinear activation functions such as ReLU, softmax, and sigmoid 190, 191. This multi-layered architecture enhances the expressive capacity of the model, enabling both low and high level representations. Low level representations, typically found in the shallow layers, capture basic features such as image edges, textures, and spatial information, while high level representations, derived from deeper layers, combine these fundamental features into more abstract and intricate concepts. This multi-level output, coupled with joint parameter optimization, makes DNNs highly effective for data analysis. Architectures like encoder-decoder models, U-Net, and generative adversarial networks (GANs) are extensively employed in biological image processing, further advancing the integration of AI with NIR-II fluorescent materials for GBM diagnosis and therapy 192. For example, Ma *et al.* highlighted that the main challenge in NIR *in vivo* imaging is the development of fluorescent probes with high biosafety, especially for achieving deep-penetration imaging in the NIR-IIb window using QDs like **PbS/CdS**.^193^ However, these QDs have not been approved by the FDA due to the presence of toxic heavy metals. In contrast, biocompatible dyes such as ICG, which are FDA-approved for shorter NIR-I or NIR-IIa regions, present a promising alternative. A natural approach to overcoming this issue is to link short-wavelength NIR images with long-wavelength NIR images through deep learning techniques. To address the challenge, they employed a deep learning network based on a GAN architecture, using a supervised image-to-image transformation algorithm. The adopted approach used training data from mice with p-FE (1000-1300 nm emission in NIR-IIa) and **PbS/CdS** QDs (1500-1700 nm emission in NIR-IIb) at the same location. The GAN iteratively minimized the loss function by using the output of the generator as input. This training process allowed the neural network to effectively reduce the cerebrovascular background signal in the NIR-IIa region, which was originally greater than 2 mm in depth, to produce results similar to those from authentic NIR-IIb data **(Figure 22)** 193.

Therefore, AI can improve the design of hybrid materials that integrate NIR-II FL with therapeutic properties such as PTT, chemotherapy, and gene therapy. By predicting how different inorganic/hybrid materials will interact with biological systems, AI can contribute to the creation of more efficient and targeted treatments. Furthermore, AI-guided simulations allow researchers to design nanomaterials with optimized properties, such as improved FL intensity, biocompatibility, and specific targeting capabilities for GBM 209. The fusion of NIR-II fluorescent materials with AI can open new avenues for the early detection and treatment of GBM, promising more personalized and effective therapeutic options for patients. By addressing these challenges, researchers can unlock the full potential of NIR-II fluorescent materials, revolutionizing GBM imaging and therapy. This multi-faceted approach offers new hope for patients, paving the way for more effective and personalized treatments.

## 6. Conclusion

The inorganic and hybrid materials for NIR-II FL imaging-guided therapies have represented a groundbreaking approach to GBM diagnosis and treatment. Inorganic nanomaterials, including QDs, RENPs, CDs, SWCNTs, MNCs, and hybrid materials have offered efficient deep-tissue imaging and targeted therapeutic capabilities. Their high photostability, tunable optical properties, and enhanced tissue penetration have enabled precise tumor visualization and improved therapeutic outcomes. When integrated with advanced therapies such as PTT, PDT, CDT, SDT, and immunotherapy, these nanoplatforms have enhanced tumor elimination efficiency while minimizing damage to healthy tissues. Additionally, combining NIR-II FL imaging with other imaging modalities like MRI has provided comprehensive diagnostic capabilities, ensuring accurate tumor detection and real-time monitoring of therapeutic interventions. Though, NIR-II fluorescent inorganic/hybrid materials have shown promise for GBM imaging-guided therapy but have also faced challenges in clinical translation. Issues like regulatory requirements, biocompatibility, long-term safety, and cost need to be addressed for better healthcare.

Clinical trials have highlighted the benefits such as improved tumor localization and enhanced survival outcomes, though non-specific tissue uptake remains a limitation. Strategies like surface modifications and biodegradable materials have ensured safe body clearance. Moreover, achieving longer emission wavelengths and combining NIR-II imaging with immunotherapy can enhance treatment efficacy. Overcoming the BBB with biomimetic coatings and targeting ligands can further improve tumor-specific delivery, offering hope for more effective, personalized GBM therapies. Furthermore, employing AI for analyzing results and diagnostic applications has the ability to precisely guide therapeutic interventions. AI also has the potential to design efficient smart materials with enhanced therapeutic outcomes for better public healthcare. The integration of modern therapeutic modalities with NIR-II FL imaging could be an ultimate theranostic approach in the near future by addressing all concerns from bench to bedside.

## Figures and Tables

**Table 1:** Comparison among different imaging modalities for GBM diagnosis.

Features	MRI	PET	Ultrasound Imaging	NIR-II FL Imaging
Resolution	1-2 mm 194	0.67-2 mm 195	50-150 μm 196	400-500 μm 197
Depth Penetration	5-7 cm 198	0.5-2 mm 195	2 cm 196	3-4 mm 197
Sensitivity	High	Very high	Moderate	High
Contrast Mechanism	T_1_/T_2_ relaxation times	Metabolic activity (e.g., glucose uptake)	Acoustic impedance	FL signal from NIR-II fluorescent probes
Real-time Imaging	No	No	Yes	Yes
Invasiveness	Minimally invasive	Invasive	Non-invasive	Minimally invasive
Radiation Exposure	None	Yes (ionizing radiation)	None	None
Tumor-Specificity	Limited	High (tumor-specific tracers)	Low	High (targeted NIR probes)
Cost and Accessibility	High and widely available	Very high and limited availability	Low and widely available	Moderate and still emerging as a promising.
Clinical Use for GBM	Standard diagnostic and follow-up tool	Used for metabolic imaging and recurrence detection	Mainly for intraoperative guidance	Emerging for real-time and high-resolution tumor imaging

**Table 2:** Different inorganic/hybrid NIR-II fluorescent probes for imaging-guided therapy of GBM.

NIR-II fluorophore	Type of Study	λ_Abs_/λ_Em_ (nm)	Cytotoxicity / Conc. (µg/mL)	BBB Crossing Mechanism	Application	Ref
NaErF_4_:Ce@NaAF_4_@NaLuF_4_	*In vivo*	808/1525	∼12% / 5000	RMT/FUS	Surgery	103
^64^Cu-DOTA-FA-ICG	*In vivo*	780/830	-- / 500	RMT	Surgery	143
Gd-Ag_2_S	*In vivo*	808/1200	-- / 1000	AMT	Surgery	142
CH4T@MOF-PEG-AE	*In vivo*	808/1050	<10% / 100	RMT	Surgery/PTT	170
DCNP@P(Se-DOX)@ANG	*In vivo*	980/1550	<10% / 300	RMT	Chemotherapy	41
PSY NPs	*In vivo*	750/1030	∼5% / ~100	RMT	Chemotherapy	171
Gd_2_O_3_:Nd^3+^ NDs	*In vivo*	808/1060	10% / 300	RMT	Radio/Chemotherapy	147
N-B-GQDs	*In vivo*	808/∼>1000	∼6% / 100	AMT	PTT	132
ApoE-Ph NPs	*In vivo*	808/1575	9.55% / 200	RMT	PTT	172
NaLnF_4_:Yb/Er@Cu_2-x_S	*In vivo*	980/1525	20% / 1000	AMT	PTT	151
BPNdDTK-cRGD	*In vivo*	808/1050	5% / 100	RMT	PDT	173
NaYF_4_:Yb,Er@NaGdF_4_:Yb@NaNdF_4_:Yb	*In vivo*	808/1064	∼18% / 1000	AMT	PDT	174
Au_1_Ag_2_NPs	*In vivo*	497/1100	3.4% / 500	RMT	PDT	175
UCNPs@RB@RGD@avidin	*In vivo*	808/>1000	10% / 250	RMT	PDT/Surgery	157
YOF:Nd^3+^@MnO_2_-ICG-GOx-LF	*In vivo*	808/1340	>10% / 400	RMT	PDT/ST	155
NaYF_4_:Yb/Tm@NaYF_4_:Nd	*In vivo*	808/1340	<20% / 200	FUS	Gas Therapy	177
PEG/LDNPs@CMSNs	*In vivo*	980/1525	~10% / 200	AMT	CDT/PDT	156
Ag_2_S-Ag@PEG	*In vivo*	808/1270	>20% / 400	AMT	CDT/PTT	162
D/UCNP@_cg_AuNCs	*In vivo*	808/1060, 1550	~10% / ~1250	AMT	Chemo/PDT	148
YVO_4_:Nd^3+^-HMME@MnO_2_-LF	*In vivo*	808/1064	5-3% / 400	RMT	SDT	34
DSNPs@MOF	*In vivo*	980/1525	10% / 500	RMT	SDT	166
EDBM-8.4 NPs	*In vivo*	980/1525	<25% / 400	FUS	Immunotherapy	169
NaGdF_4_:Yb/Er/Ce@NaYF_4_:Nd@NaGdF_4_	*In vivo*	808/1525	<15% / 200	RMT	Imaging	178
Mn^2+^-Apo-Lf-PEG	*In vivo*	600-900/∼1020	∼2% / 2000	RMT	Imaging	179
TB1-RGD dots	*In vivo*	740/975	<10% / 100	RMT	Imaging	180
NaNdF_4_@NaLuF_4_/IR-808@DSPE-PEG_5000_	*In vivo*	808/1340	<20% / 200	RMT	Imaging	24
4C Au nanohybrids	*In vivo*	808/1100	-- / 40	AMT	Imaging	137
Au_25_ cluster	*In vivo*	808/1120	-- / 5300	RMT	Imaging	114

Receptor Mediated Transport (RMT); Absorptive Mediated Transport (AMT)
